# Sustainable Design of New Ionic Forms of Vitamin B_3_ and Their Utilization as Plant Protection Agents

**DOI:** 10.1021/acs.jafc.2c01807

**Published:** 2022-06-29

**Authors:** Witold Stachowiak, Damian Krystian Kaczmarek, Tomasz Rzemieniecki, Michał Niemczak

**Affiliations:** Department of Chemical Technology, Poznan University of Technology, Berdychowo 4, Poznan 60-965, Poland

**Keywords:** phytotoxicity, sustainable chemistry, synthesis design, surface activity, volatility

## Abstract

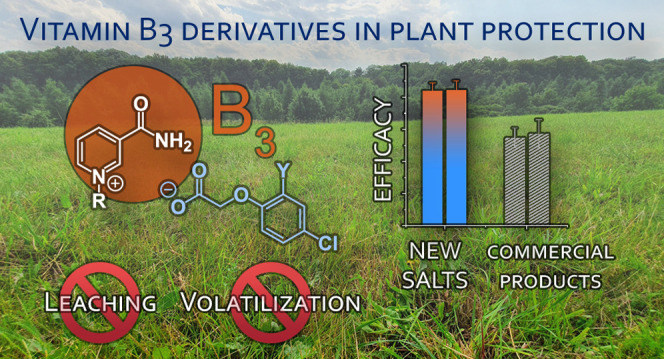

This study demonstrates the utilization of naturally occurring nicotinamide (vitamin B_3_) in the sustainable synthesis of organic salts with application potential as environmentally friendly agrochemicals. The designed ionic pairs, obtained with high yields, consisted of *N*-alkylnicotinamide cation and commercially available herbicidal anions: 2,4-dichlorophenoxyacetate (2,4-D) and 4-chloro-2-methylphenoxyacetate (MCPA). The study confirmed the strong influence of the length of alkyl chain in products on the physicochemical properties as well as the development of cornflower and oil-seed rape. The majority of tested salts showed significantly better herbicidal activity (by approx. 30–50%) compared to the reference herbicide. Furthermore, *N*-hexadecylnicotinamide 4-chloro-2-methylphenoxyacetate was significantly more effective than the commercial formulation in the dose–response test. Their negligible vaporization, multiple times lower than that of commonly used dimethylammonium salts, eliminates one of the greatest threats of currently applied plant protection agents. Additionally, the risk of product migration or bioaccumulation in the environment was assessed as extremely low.

## Introduction

Many currently used biologically active chemical compounds, including antibiotics, fungicides, pesticides, and pharmaceuticals, are characterized by numerous traits that are considered undesirable from an environmental point of view. These can be the result of very low efficacy, toxicity to nontarget organisms, and low biodegradability or biocompatibility.^1^ Therefore, the search for alternative chemicals with similar effects that can be manufactured and subsequently utilized in a safer manner becomes a necessity. Since new compounds should be designed to avoid chemical hazards,^2^ it is essential to consider both the high efficiency of the designed products and their low toxicity with simultaneous high biocompatibility.

Thus, the use of substances of natural origin as substrates has become a common strategy in recent years. Natural compounds that do not adversely affect human health, such as terpenes,^3,4^ glycosides,^5−7^ essential oil components,^8,9^ or vitamins,^10^ are considered favorable raw materials in sustainable chemical processes. It should also be noted that the newly developed chemicals must also be obtained in an efficient and environmentally friendly manner. This indicates, among other things, the use of nontoxic reaction media,^11,12^ reduction of energy consumption, and the appropriate management of the resulting waste.^2^ Skillful application of the approach described above can result in the development of more effective chemicals with significantly reduced risks associated with their use.

This strategy includes derivatization of nicotinamide (NA), commonly known as a form of vitamin B_3_. NA, widely present in food, is often used as a dietary supplement and medication to prevent and treat pellagra,^13^ neurodegenerative diseases (i.e., Alzheimer’s and Parkinson’s diseases),^14,15^ and psychological disorders.^13^ NA is also present in plants, especially mature cereal grains such as corn and wheat, where it is bound to sugar molecules in the form of glycosides.^16^ NA is a fully biocompatible and low-toxic substance; its safe daily dose reaches up to 1000 mg kg^–1^ body weight.^13^ One of the methods of derivatization of NA is the alkylation of the aromatic nitrogen atom present in its structure; this results in the transformation of the amide into a cationic form and allows it to combine with the appropriate counter ion conditioning a specific type of biological activity.^17^

Transformation of known, nontoxic chemicals into ionic forms is an effective strategy for the synthesis of new biologically active substances with tuned physicochemical properties. This approach is used, among others, in the synthesis of new plant protection agents: systemic resistance inducers^1,18^ and herbicides.^19^ In such substances, one of the ions is characterized by high biological activity, and the other one acts as an effective adjuvant or active substance with complementary activity. The use of such products allows for a strong, selective biological response without the use of auxiliary chemicals such as surfactants. This is all the more important since numerous adjuvants to crop protection products can exhibit increased detrimental effects compared to the active ingredient itself.^19^

The strategy described above can be realized in a particularly effective way by combining the previously designed cation with an anion derived from an herbicidal synthetic auxin, such as 2,4-dichlorophenoxyacetic acid (2,4-D) and 4-chloro-2-methylphenoxyacetic acid (MCPA). Both of these herbicides are popular, effective, and readily biodegradable in the environment. In addition, it has been repeatedly confirmed that their transformation to ionic forms can result in a significant increase in their biological activity.^19^ Moreover, innovative “green” methods allowing combining the herbicide anion with an appropriate cation have already been developed,^10,20^ and an appropriate selection of the cation chemical structure may positively influence a number of physicochemical and biological parameters of the active substance, especially important from the environmental protection point of view, i.e., toxicity, biodegradability, and mobility in soil.^19^ Interestingly, the introduction of a quaternary ammonium cation derived from natural resources can result in a highly beneficial modification of the above-mentioned parameters. It should also be noted that from the point of view of sustainability, such compounds should be obtained in a highly efficient manner and the designed synthesis method should include the principles of green chemistry to minimize any potential negative impact associated with their production.

The goal of the study was to synthesize new bioinspired salts that contain vitamin B_3_ as the cation and two anions known as popular agrochemicals (2,4-D and MCPA). A literature survey on the derivatization of nicotinamide indicates that there are scarce data demonstrating the biological activity of this type of compound, including its phytotoxic effect on dicotyledonous plants. Furthermore, it was hypothesized that the presence of the long alkyl chain in the cation in the designed ionic pairs should bring exceptional benefits, such as the possibility of adjusting physicochemical properties (e.g., melting point, solubility in water) or surface activity, which are known to be essential in applications aimed at protecting crops from various pests.

## Experimental Section

### Materials

1-Bromodecane (98%), 1-bromododecane (97%), 1-bromotetradecane (97%), 1-bromohexadecane (97%), 1-bromooctadecane (97%), and 3-pyridinecarboxamide (nicotinamide, 98%) were purchased from Sigma-Aldrich (Saint Louis, Missouri). 2,4-Dichlorophenoxyacetic acid (2,4-D, 98%) and 4-chloro-2-methylphenoxyacetic acid (MCPA, 96%) were obtained from PESTINOVA (Jaworzno, Poland). Both herbicidal acids were purified prior to use by recrystallization from toluene followed by treatment with activated carbon (powder—100 particle size (mesh), Sigma-Aldrich, Saint Louis, Missouri). All solvents (methanol (99.8%), dimethyl sulfoxide (DMSO) (98%), acetonitrile (99%), acetone (99%), isopropanol (98%), *n*-propanol (99.5%), ethyl acetate (99%), chloroform (98%), toluene (99.5%), hexane (99.5%)) and potassium hydroxide (85%) were delivered by Avantor (Gliwice, Poland) and used without further purification. Deionized water with a conductivity < 0.1 μS cm^–1^, from the HLP Smart 1000 demineralizer (Hydrolab, Poland), was used.

### Methods

#### Spectral Analysis

UV spectra were recorded in the region from 200 to 400 nm at 25 °C with a Rayleigh UV-1601 spectrophotometer (Beijing Beifen-Ruili Analytical, China) using 1 cm path length quartz cuvettes. ^1^H NMR spectra were recorded on a VNMR-S spectrometer (Varian) operating at 400 MHz with tetramethylsilane (TMS) as the internal standard. ^13^C NMR spectra were obtained with the same instrument at 100 MHz. Deuterated chloroform or DMSO was used as a solvent for NMR analysis. IR spectra were collected using a semiautomated EasyMax 102 system (Mettler Toledo, Switzerland) connected to the ReactIR iC15 probe equipped with an MCT detector and a 9.5 mm AgX probe with a diamond tip. Neat samples of compounds were used for analysis. The data were sampled from 3000 to 650 cm^–1^ with 8 cm^–1^ resolution and processed by the iCIR 4.3 software.

#### Melting Point

The melting points of the compounds obtained were analyzed *via* an MP 90 melting point system (Mettler Toledo, Switzerland). The precision of the measurements was ensured by calibration of the apparatus using certified reference substances.

#### Water Content

The water content in all obtained products was measured with a TitroLine 7500 KF trace apparatus (SI Analytics, Germany) using the Karl Fischer titration method. First, each compound was dissolved in dehydrated methanol. The water content was determined in the pure methanol as well as in the obtained methanolic solutions. On the basis of the collected results, the water content in pure products was calculated.

#### Cationic Surfactant Content

The cationic surfactant content was assayed by a direct two-phase titration technique according to EN ISO 2871-1:2010. The method is based on the titration in the biphasic (water–chloroform) system (or water–methanol–chloroform in the case of compounds with limited solubility in water) of the solution of the amphiphilic ammonium salt by a standard solution of sodium dodecylsulfate(VI) in the presence of the mixed indicator: dimidium bromide indicator (CAS: 518-67-2)—for the determination of the cationic active moiety and sulfan blue indicator (CAS: 129-17-9)—for the determination of the anionic active moiety.

#### Solubilities

The solubility of the prepared ionic liquids (ILs) in 10 representative solvents was determined according to the protocols available in Vogel’s Textbook of Practical Organic Chemistry.^21^ The solvents chosen for this study were organized in order of decreasing value of their Snyder polarity index: water—9.0, methanol—6.6, DMSO—6.5, acetonitrile—6.2, acetone—5.1, chloroform—4.4, ethyl acetate—4.3, isopropanol—4.3, toluene—2.3, and hexane—0.0.^22^ A 0.10 g sample of each compound was added to a specific volume of a solvent, and the samples were thermostated in Water Bath MEMMERT Model WNB 7 at 25 °C. Based on the volume of solvent used, the appropriate class of solubility was typed: “ready solubility” refers to compounds that dissolved in 1 cm^3^ of solvent (>10% m/v), “limited solubility” refers to compounds that dissolved in 3 cm^3^ of solvent (3.3–10% m/v), and “low solubility” refers to the compounds that did not dissolve in 3 cm^3^ of solvent (<3.3% m/v).

#### Octanol–Water Partition Coefficients

The octanol–water partition coefficients (*K*_OW_) of the synthesized products, as well as the potassium salt of 2,4-D and MCPA were estimated by the shake-flask method according to the OECD guidelines.^23^ Measurements of *K*_OW_ values were performed using mutually saturated distilled water and *n*-octanol in a glass vial containing a magnetic stir bar. First, the synthesized products, as well as potassium salts of 2,4-D and MCPA (**[K][2,4-D]** and **[K][MCPA]**) were dissolved in 4 cm^3^ of distilled water at concentrations corresponding to the dose applied in greenhouse experiments (10 mM), and then 4 cm^3^ of octanol was added. Subsequently, two duplicate runs were carried out with different solvent ratios: 4 cm^3^ of octanol and 2 cm^3^ of water (2:1), and 2 cm^3^ of octanol and 4 cm^3^ of water (1:2). All vials have been shaken at a constant temperature of 25 °C. After 15 min., all samples were centrifuged and the aqueous and octanolic phases were collected by a syringe. The concentrations of compounds in water were determined spectrophotometrically using a Rayleigh UV-1601 spectrophotometer (Beijing Beifen-Ruili Analytical, China) (based on calibration curves made previously (at λ_max_ = 265 nm in the case of bromides and 2,4-D and at λ_max_ = 264–279 nm in the case of MCPA) vs concentration for each substance). Two repetitions of each measurement were performed in a specific solvent ratio (1:1, 1:2, and 2:1). Finally, the log *K*_OW_ was calculated as the average of six results collected for each compound.

#### Volatility

The scheme demonstrating a developed installation for experiments regarding the volatility of the obtained products is presented in Figure S57, Supporting Information (SI). First, appropriate solutions containing herbicidal substances at a concentration of 2.0 g of active ingredient per liter (which corresponds to 400 g per hectare used in the greenhouse experiment) were obtained by the dissolution of products **6**, **10**, **11**, **15**, and dimethylammonium salts of 2,4-D and MCPA in water (due to the extremely low solubility of free acids of 2,4-D and MCPA in water, these compounds were dissolved in a mixture of water and isopropanol in a ratio of 5:1 (v:v)). Then, 60 cm^3^ of the selected solution was poured into a glass bottle that was thermostated at 40 °C. A glass bottle was tightly connected to the receiving scrubber that collected the air that passed over the solution of herbicide. The installed pump provided constant airflow, whereas the valve enabled adjustment of the flow rate (which amounted to 106.6 cm^3^ min^–1^) and maintenance of the equivalent conditions for all tested solutions. The air was passed through the system for 4 h, and then the solution-receiving scrubber was analyzed *via* UV spectroscopy (Rayleigh UV-1601 spectrophotometer (Beijing Beifen-Ruili Analytical, China)). To determine the limit of detection (LOD) for the UV spectrometer, 10 independent measurements were made for a series of blank samples. The LOD, determined from the following formula
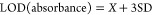
1where *X* is the mean of 10 measurements and SD is the standard deviation, was 0.0018.

Subsequently, based on the elaborated curves for 2,4-D and MCPA, presented in Figure S58 (SI), and the absorbance in the receiving scrubber at 280 nm, the LOD of herbicidal acids (in mg per liter), as well as the precise concentration of the herbicidal acid in the receiving scrubber were calculated.

### Greenhouse Experiments

#### Preliminary Test

Cornflower (*Centaurea cyanus* L.) and oil-seed rape (*Brassica napus* L.) plants were used to test the biological activity of the examined compounds. The seeds of the selected plants were seeded in plastic pots (1.0 dm^3^, with a diameter of 15 cm) containing a peat-based substrate. The plants were grown in a greenhouse with a photoperiod of 16 h day/8 h night. The temperature was maintained at 25 ± 2 °C during the day and at 20 ± 2 °C during the night. Relative air humidity was approximately 60–80%. Soil moisture was maintained at 65–75% of the soil water capacity. The seedlings were thinned 2 weeks after emergence to six uniform plants per pot. The greenhouse trial was designed as a complete randomized complete block with four replications. Herbicides were applied when the plants were in the four-leaf stage using a laboratory sprayer using TeeJet 1102 nozzles (TeeJet Technologies, Germany) delivering 200 dm^3^ ha^–1^ at 0.2 MPa. All tested products (**6**–**15**) were dissolved in water at amounts that correspond to a dose of 400 g a.i. (active ingredient) per hectare. The commercial products: Chwastox Extra 300 SL (containing sodium-potassium salt of MCPA at a concentration of 300 g dm^–3^; Ciech, Poland) and Aminopielik Standard 600 SL (containing dimethylammonium salt of 2,4-D at a concentration of 600 g dm^–3^; ADAMA Manufacturing Poland, Poland) were used at the same dose of the active ingredient. After the experiments, the plants were weighted to assess the percentage of reduction in fresh weight reduction in comparison to the control (plants treated with placebo). The weed control was evaluated 21 days after treatment (DAT) using a scale of 0 (no effect) to 100% (completely destroyed plant). Each error margin range represents the standard error of the mean (SEM). The SEM values were calculated according to the following equation
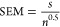
2where SEM is the standard error of the mean, *s* is the sample standard deviation, *n* is the number of samples.

#### Dose–Response Test

In the next stage of the experiment, the most active compound *N*-hexadecylnicotinamide 4-chloro-2-methylphenoxyacetate (**14**) and the commercial preparation containing the same active ingredient (Chwastox Extra 300 SL) were applied at various doses equal to: 0.5 n; 1.0 n; 1.5 n; 2.0 n; and 2.5 n (where: *n* = 400 g of active ingredient (MCPA) per ha). The plants of cornflower (*C. cyanus* L.) as well as oil-seed rape (*B. napus* L.) were used for the experiments. The general conditions of the experiment as well as the equipment were analogous to those in the case of preliminary experiment. Twenty-one days after treatment, the plants were weighted to determine the percentage of reduction in fresh weight reduction in comparison to the control. For both tests (preliminary and dose–response), data were subjected to analysis of variance (ANOVA) followed by Tukey’s protected least significant difference (LSD) test at the probability level of 0.05.

#### Surface Activity

Measurements of surface tension and contact angle were carried out using a DSA 100E analyzer (Krüss, Germany, accuracy ± 0.01 mN m^–1^) at 25 °C, following a methodology described recently.^24^ Surface tension was determined by the drop shape method. The method depends on the formation of an axisymmetric drop at the tip of the needle of a syringe, after which the image of the drop is acquired with a USB 3 uEye CP camera (IDS Imaging Development Systems GmbH, Obersulm, Germany) and digitized. The surface tension (γ in mN m^–1^) was calculated based on an automatic analysis of the drop profile according to the Laplace equation. The determination of the contact angle was based on the sessile drop method. The principle of this method is to deposit drops of liquid on a solid hydrophobic surface (paraffin, the hydrophobic surface that serves as the model surface of leaves).

#### Preparation of *N*-Alkylnicotinamide Bromides (**1**–**5**)

*N*-alkylnicotinamide bromides (**1**–**5**) were synthesized using an EasyMax reactor (Mettler Toledo, Switzerland) *via* the quaternization reaction of nicotinamide using an appropriate linear 1-bromoalkane (from C_10_H_21_Br to C_18_H_37_Br). Nicotinamide (0.20 mol) and 1-bromoalkane (0.21 mol) were mixed with 20 cm^3^ of *n*-propanol, and the obtained mixture was heated at 97 °C under reflux for 12 h. After the solution was cooled, the precipitate was filtered off and washed three times with 10 cm^3^ portions of acetone (1-bromoalkanes present in the postreaction mixture are soluble in acetone while the obtained bromides are insoluble). Finally, the product was dried under vacuum (5–10 mbar) at 50 °C for 24 h.

#### Preparation of *N*-Alkylnicotinamide Phenoxyacetates (**6**–**15**)

All reactions were conducted using an EasyMax reactor (Mettler Toledo, Switzerland) equipped with a pH meter, because at pH > 7, the nicotinamide moiety undergoes rapid decomposition leading to the formation of red-brown impurities.^25^ Initially, the selected herbicidal acids (2,4-D and MCPA) were neutralized with stoichiometric amounts of potassium hydroxide in methanol. Then, the solvent was evaporated using a rotary vacuum evaporator. The obtained potassium salts of the selected acids (**[K][2,4-D]** and **[K][MCPA]**) were dried in a vacuum oven at 40 °C for 48 h. Next, the appropriate *N*-alkylnicotinamide bromide (0.01 mol) was dissolved in 10 cm^3^ of methanol in a 100 cm^3^ reaction vessel equipped with a mechanical stirrer. Next, a 2% molar excess (0.0102 mol) of **[K][2,4-D]** and **[K][MCPA]**, dissolved in 10 cm^3^ of methanol, was added to perform the ion exchange reaction. The reaction mixture was stirred at 50 °C for 15 min and then cooled to 0 °C. As a result of anion exchange, a sediment of potassium bromide precipitated from the postreaction mixture. Subsequently, the inorganic salt was filtered off and the solvent was evaporated from the filtrate. The obtained products were additionally purified by the addition of a small portion (10–15 cm^3^) of a mixture of acetone and isopropanol 2:1 (v:v), which allowed us to isolate the traces of inorganic impurities and excess reactant through vacuum filtration. After evaporation of solvents, the obtained products were dried at 50 °C for 24 h under reduced pressure (1–2 mbar). All synthesized salts were stored in a vacuum desiccator with a drying agent (P_4_O_10_).

## Results and Discussion

### Synthetic Strategy for *N*-Alkylnicotinamide Phenoxyacetates

The constantly growing interest in developing environmentally friendly plant protection agents originates from the desire to seek safer alternatives to commercial forms that may pose a threat to living organisms (e.g., by volatilization or leaching from the soil). This fact encouraged us to focus attention on the design and synthesis of more sustainable and green ionic forms of selected popular herbicides (2,4-D and MCPA) and subsequent analysis of their physicochemical properties, herbicidal activity, and safety. The widespread occurrence of NA in the environment, combined with its beneficial influence on human health and low toxicity (therapeutic doses can reach even 3000 mg day^–1^), allows us to classify it as an extremely promising substance for utilization as an agrochemical, such as an herbicidally active substance. However, to achieve this goal in this particular application, the structure of NA had to be subjected to two appropriate modifications: (1) knowing that the active substance requires the use of additional adjuvants responsible for surface activity and good wetting properties,^19^ NA was substituted with long hydrophobic alkyl chains to obtain cations exhibiting amphiphilic properties; (2) the subsequent combination of these cations with well-known, commercial selective herbicides from the group of phenoxy acids, such as 2,4-D or MCPA. This approach has resulted in the formation of novel agrochemicals that do not require activity enhancers and should simultaneously possess favorable physicochemical characteristics, such as negligible vapor pressure and low risk of bioaccumulation in the environment or groundwater contamination.

### Synthesis

**Figure 1:**
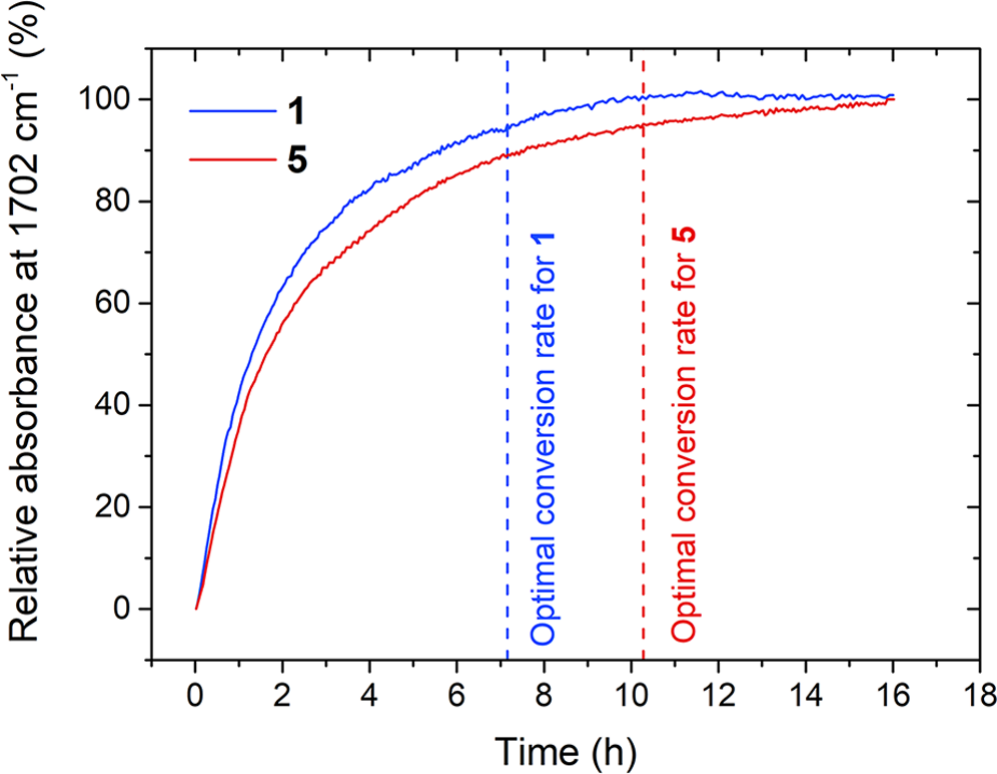
Changes in relative absorbance at ν = 1702 cm^–1^ for quaternizations conducted at 97 °C in *n*-propanol at a concentration of 2 M. Optimal conversion time refers to 95% conversion of reactants.

**Scheme 1:**
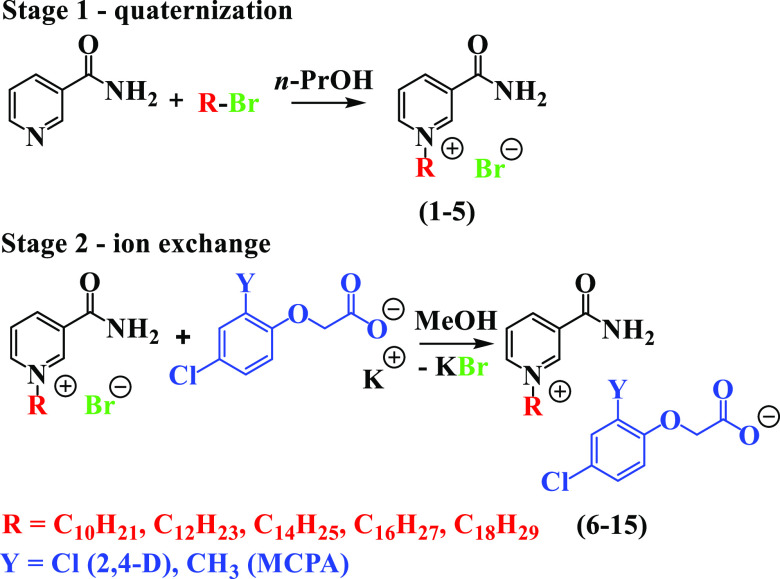
Synthesis of *N*-Alkylnicotinamide Phenoxyacetates

During the first step, a homologous series of *N*-alkylnicotinamide bromides comprising an alkyl chain varying from decyl (**1**) to octadecyl (**5**) was synthesized *via* the quaternization reaction of nicotinamide with an appropriate bromoalkane (Scheme 1). Salts **1**–**4** have been synthesized and characterized in a previous study, in which they exhibited the ability to reduce the surface tension of aqueous solution and have been classified as cationic surfactants.^26^ However, known methods of their synthesis require the use of solvents that are currently considered problematic or hazardous,^12,27^ such as dimethylformamide, tetrahydrofuran, or xylene.^26,28^ In accordance with the concepts of green chemistry and sustainable development, we focused our attention on the design of a highly efficient process that utilizes inexpensive, fully renewable, biodegradable, and safe solvents.^27^ During the initial tests, *n*-propanol (*n*-PrOH) was selected as the most promising reaction medium (see Table S1, SI). Subsequently, the optimal time of the reactions carried out in this solvent was determined with the use of an EasyMax reactor equipped with a Fourier transform infrared (FT-IR) spectroscopy probe. As shown in Figure 1, the developed new, sustainable method of synthesis of *N*-alkylnicotinamide bromides (**1**–**5**) allows us to achieve yields ranging from 80 to 99% (depending on the length of the alkyl chain) in less than 10 h.

The second step involved a metathesis (ion exchange) reaction carried out in an EasyMax reactor in methanol (MeOH), in which the bromide anion in **1**–**5** was replaced with one of the following herbicidal ions: 2,4-D (**6**–**10**) or MCPA (**11**–**15**). The pH of all reaction mixtures had to be carefully maintained (slightly below the value of 7) because the nicotinamide moiety undergoes rapid decomposition in the basic environment.^25^ After a two-stage purification from inorganic residues, the obtained products were dried in vacuum and stored over P_4_O_10_ (details of the syntheses are provided in the Experimental Section). Eventually, all salts (**6**–**15**) were obtained in high yields greater than 85% (see Table 1).

**Table 1:** Synthesized Salts Comprising *N*-Alkylnicotinamide as the Cation and Bromide (**1**–**5**), 2,4-D (**6**–**10**), or MCPA (**11**–**15**) as the Anion

salt	R	anion	yield (%)	melting point (°C)	water content (%)
**1**	C_10_H_21_	Br	80	205^c^	0.613
**2**	C_12_H_25_		84	207^c^	0.971
**3**	C_14_H_29_		89	206^c^	0.484
**4**	C_16_H_33_		96	209^c^	1.077
**5**	C_18_H_37_		99	198^c^	0.900
**6**	C_10_H_21_	2,4-D^a^	90	126–128	1.818
**7**	C_12_H_25_		97	143–146	2.410
**8**	C_14_H_29_		89	126–128	2.967
**9**	C_16_H_33_		96	119–121	2.743
**10**	C_18_H_37_		92	60–62	1.411
**11**	C_10_H_21_	MCPA^b^	92	95–97	1.797
**12**	C_12_H_25_		86	103–105	1.686
**13**	C_14_H_29_		87	95–97	2.627
**14**	C_16_H_33_		95	92–95	2.230
**15**	C_18_H_37_		87	100–102	2.157

a 2,4-Dichlorophenoxyacetate.

b 4-Chloro-2-methylphenoxyacetate.

c Decomposition.

According to the data in Table 1, *N*-alkylnicotinamide bromides (**1**–**5**) were white solids with a melting point ranging from approx. 198 °C (for a salt **5**) to approx. 209 °C (for a salt **4**). It should be noted that their melting was accompanied by simultaneous rapid degradation to red-brown decomposition products, independent of the length of the alkyl substituent. Furthermore, all synthesized salts with phenoxyacetate anions (**6**–**15**) turned out to be solids at room temperature with melting points varying from approx. 60 to 146 °C for 2,4-D-based salts (**6**–**10**) or from approx. 92 to 105 °C for MCPA-based salts (**11**–**15**). We also noted that the replacement of halogen (bromide) for structurally more complex organic anions (2,4-D or MCPA) resulted in a substantial decrease in the melting points of the obtained salts. A similar trend has been repeatedly reported for other salts comprising various tetraalkylammonium, piperidinium, or imidazolium cations and can be explained by the fact that the precisely chosen combination of anions and cations can destabilize the solid phase of the formed crystal.^29^ Moreover, the melting points of salts **10**, **11**, **13**, and **14** were below 100 °C, allowing them to be classified as ionic liquids (ILs).

Examination of the water content in the obtained salts showed that *N*-alkylnicotinamide bromides (**1**–**5**) contain approx. 0.5–1.0% water. Interestingly, the water content in salts with herbicidal anions (**6**–**15**) was multiple times greater and varied from approx. 1.5% to approximately 3%. It was also noted that **6**–**15** showed negligible hygroscopicity in the access of air. Thus, it can be concluded that water is not absorbed on the surface of the synthesized compounds, but rather bound to their crystal lattice mainly in the form of hemihydrate (∼1.5% of water) or hydrate (∼3% of water). This hypothesis is also consistent with the sharp melting points observed in the obtained products.

According to a previous report,^30^ nicotinamide derivatives substituted with the 1-alkylthiomethyl group exhibited poor stability in aqueous solution at elevated temperature (<70 °C), resulting in the cleavage of the alkyl chain from the nitrogen atom. In effect, the stability of the 10 mM aqueous solutions of the obtained products (**6**–**15**) was thoroughly examined. However, after 3 h of heating the samples at 80 °C, no signs of decomposition were observed using the direct two-phase titration technique. This means that the absence of a heteroatom in the carbon chain effectively prevents this type of degradation.

### Spectral Analysis of Synthesized Salts

The structures of the synthesized products were confirmed by UV, FT-IR, ^1^H, and ^13^C NMR spectroscopy. All spectra for salts **1**–**15** as well as their descriptions are provided in the Supporting Information (Figures S1–S56 and Tables S2–S27).

**Figure 2:**
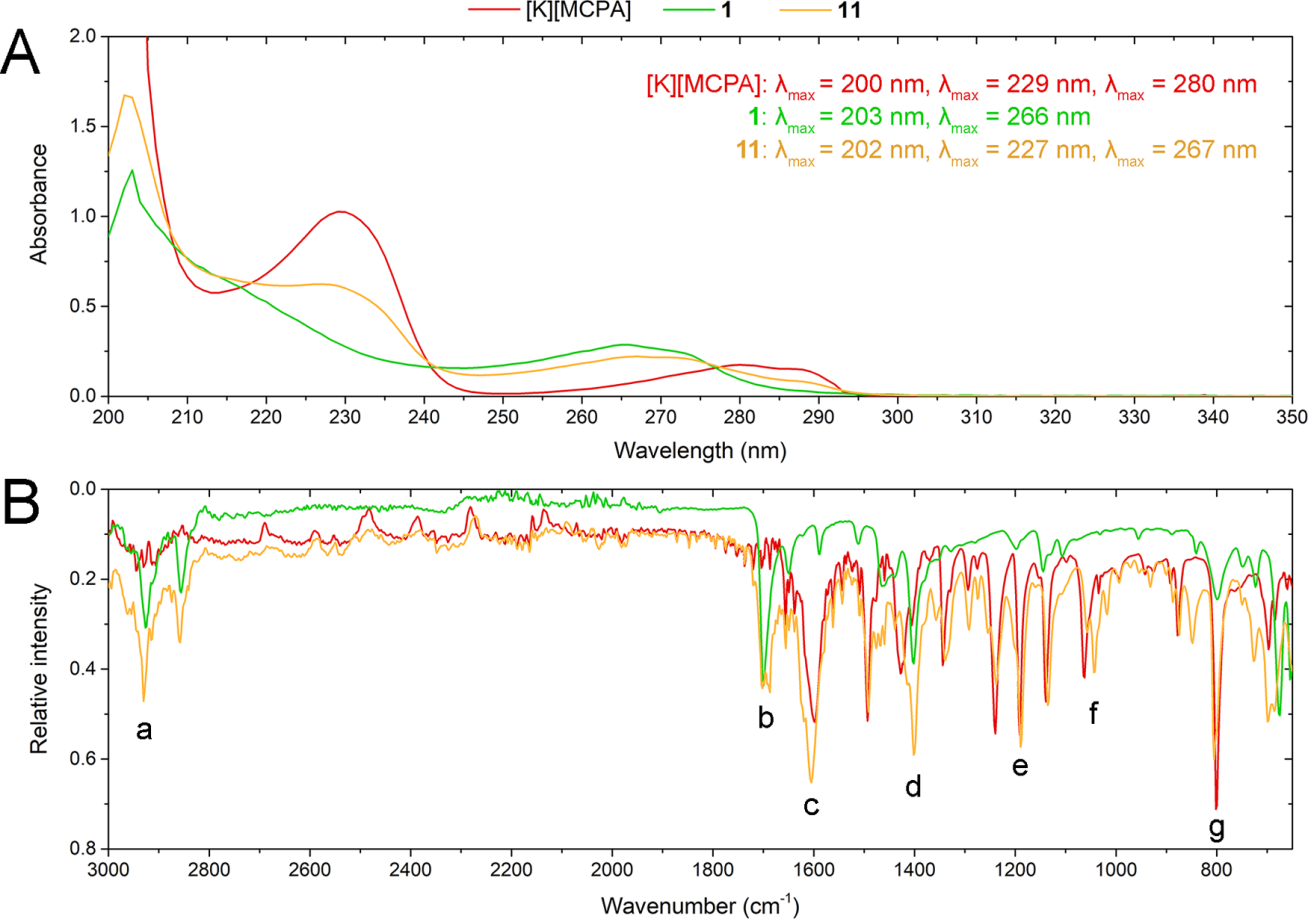
Comparison of UV (A) and FT-IR (B) spectra of MCPA potassium salt (**[K][MCPA]**), *N*-decylnicotinamide bromide (**1**), and their final product (**11**).

Benzene exhibits two intense absorption maxima at 180 nm (E_1_ band) and 204 nm (E_2_ band) and one weak absorption band at 256 nm (B band). The presence of auxochromic substituents such as halogen or ether causes a bathochromic shift in these bands.^31^ In effect, the utilized substrates—potassium salts of phenoxy acids (**[K][2,4-D]** and **[K][MCPA]**)—possessed three absorption maxima at approximately 200, 230, and 280 nm (Table S2, SI). On the other hand, the pyridinium cation has one absorption maximum above the wavelength of 200 nm (255 nm, π → π* B band).^32^ The absorption maxima of the tested *N*-alkylnicotinamide bromides (**1**–**5**) occurred at approx. 202 nm (K band) and 266 nm (B band). The spectra of the tested products with herbicidal anions (**6**–**15**) were found to be similar to those of the reactants utilized for the second stage of synthesis (see Figure 2A and Table S2, SI). However, in the case of salts with 2,4-D (**6**–**10**), the absorption maximum of the E band occurs below a detection limit (<200 nm). Moreover, the peak of the K band at approximately 230 nm was not determined because no distinctive maximum was observed in this region. The calculated molar absorptivities for all obtained salts are provided in Table S2 (SI).

Analysis of the FT-IR spectra of the obtained products **1**–**15** is summarized in Table S3 (SI). An exemplary comparison between the spectra of the product comprising the MCPA anion (**11**) with bromide (**1**) as well as **[K][MCPA]** is presented in Figure 2B. In the spectra, we can distinguish two characteristic bands originating from C–H stretching as well as bending vibrations of the alkyl substituent that occurred at 2800–3000 cm^–1^ (a) and 1400 cm^–1^ (d), accordingly. The analysis also confirmed the presence of a signal from stretching vibrations of the C=O bond from the amide at 1700 cm^–1^ (b). Moreover, the spectra of both **[K][MCPA]** and product **11** possessed a signal derived from stretching vibrations (c) (1600 cm^–1^) of the C=O bond of the carboxylate group. There are also bands derived from the asymmetric (e) (1200 cm^–1^) and symmetric (f) (1050 cm^–1^) vibrations of the C–O ether group present in the MCPA anion. Another intensive peak from the stretching vibrations of the C–Cl bond was observed at 800 cm^–1^ (g). It should also be emphasized that a direct comparison of the FT-IR spectra of designed homologous series **1**–**5**, **6**–**10**, and **11**–**15** (see Figures S55 and S56, SI) revealed a successive increase in the intensity of the bands at 2800–3000 cm^–1^ as the length of the alkyl group in the cation increases, which complies with previous reports.^33,34^

The NMR spectra confirmed the presence of the *N*-alkylnicotinamide cation in the obtained products, and in the case of salts **6**–**15**, the presence of the appropriate herbicidal anions. A detailed analysis of the spectra of **1**, **3**, and **6**–**15** is provided in Tables S3–S26 (SI). In the collected ^1^H NMR spectra, characteristic signals from the *N*-alkylnicotinamide cation occurred at the following chemical shifts: 0.9 ppm (triplet from the CH_3_ group present in the alkyl), 4.7 ppm (triplet from the CH_2_ group attached to the nitrogen), and 7.4–10.0 ppm (two singlets from two protons of the amide group). The main difference in the collected spectra for salts **1**–**5**, **6**–**10**, and **11**–**15** was the observed increase in the integration of the signal at approximately 1.2 ppm as a result of the extension of the chain length in the homologous series. It is also worth mentioning that the protons from the amide group were present in the spectra as two separate singlets. This phenomenon can be explained by the fact that the structure of the nicotinamide moiety exhibits a flat spatial orientation.^35^ In effect, one of the protons in the amide group is oriented toward the aromatic ring, while the second is oriented in the opposite direction. Moreover, in the ^1^H NMR spectra of products **6**–**15**, the following signals originating from the herbicidal anions could be observed: 2.1 ppm (singlet from the CH_3_ group in the MCPA anion, present only in products **11**–**15**), 4.5 ppm (singlet from the CH_2_ group), and 6.7–7.5 (doublets originating from protons in the aromatic ring).

The ^13^C NMR spectra of bromides (**1**, **3**) revealed multiple peaks in a range of approx. 14 to 61 ppm, which can be attributed to carbon atoms from the alkyl substituent. Another five signals from approx. 128 to 146 ppm originated from carbon atoms present in the aromatic ring. The carbon atom from the amide group exhibited the greatest value and appeared at approx. 163 ppm. In the case of products **6**–**15**, it is possible to confirm the presence of the appropriate herbicidal anion based on the additional peaks noted at 16 ppm (from the carbon atom in the CH_3_ group of the MCPA anion), 68–69 ppm (from the carbon atom in the CH_2_ group), 112–156 ppm (six signals from the carbon atoms in the aromatic ring), and 171–173 ppm (signal from the carbon atom in the carboxylate group). In conclusion, the collected results of the spectral analysis performed *via* multiple spectral techniques are irrefutable proof that compounds characterized by a designed strategy were obtained successfully.

### Solubility

Data regarding the affinity of new chemical compounds with different solvents can be particularly useful in the case of experiments aimed at discovering directions of their effective applications, including agricultural purposes. Therefore, the solubilities of the obtained salts comprising bromide anions (**1**–**5**) as well as herbicidal anions (**6**–**15**) were determined in 10 representative solvents exhibiting various polarity indices. As shown in Table S28 (SI), the synthesized bromides (**1**–**5**) exhibited good solubility only in organic polar solvents, such as methanol and DMSO. A thorough comparison of the solubilities of bromides (**1**–**5**) with salts comprising herbicidal anions (**6**–**15**) allowed us to elucidate the impact of ion exchange on this parameter. The incorporation of the phenoxyacetate anion was found to increase compounds’ affinity for semipolar solvents, such as chloroform and isopropanol. Generally, according to previous reports, the replacement of halogen for organic, structurally more complex anions leads to a substantial decrease in water solubility.^33,36^ However, in the case of the obtained products (**6**–**15**), the opposite effect was observed, and their affinity to water was greater than that of bromides (**1**–**5**). Additionally, it was also found that synthesized salts with longer alkyls (**3**–**5**, **10**, **14**, and **15**) exhibit notably lower solubility in this solvent and that *N*-hexadecylnicotinamide 2,4-chlorophenoxyacetate (**9**) was readily soluble in water unlike its MCPA counterpart (**14**). This means that the effect of the anion becomes apparent only with a sufficiently long alkyl chain. Interestingly, the structure of the anion did not influence solubility in the other semipolar (acetonitrile and acetone) or less polar (ethyl acetate, toluene, and hexane) organic solvents—the obtained products (**1**–**15**) were not miscible with any of them.

### Octanol–Water Partition Coefficient

After treatment, plant protection agents not only accumulate in crops but can also be transported through air, soil, and water over long distances, constituting a major source of pollution in ecosystems. The latest recommendations in agrochemistry require an assessment of the hydrophilicity of pesticides since this parameter can be of great importance for their transfer to the environment. Among many, the logarithm of the octanol–water partition coefficient (log *K*_OW_) is the most popular factor that allows us to describe the tendency of the distribution of a solute from the aqueous phase into organic constituents of environmental compartments.^1,19,37^ In effect, highly hydrophilic compounds, characterized by log *K*_OW_ lower than 0, exhibit high hydrophilicity and are known to easily permeate through the soil, which poses a threat to groundwater pollution. In contrast, highly lipophilic substances, characterized by log *K*_OW_ greater than 3, are likely to have optimal parameters for bioaccumulation in marine and terrestrial food chains.^37^

**Figure 3:**
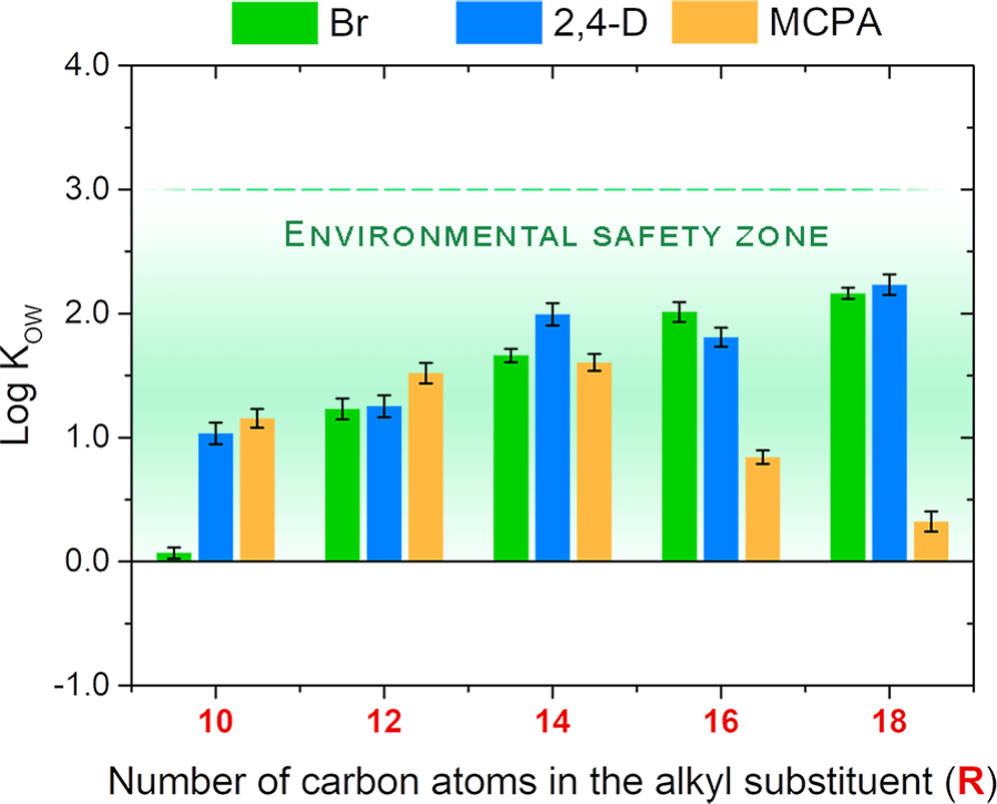
Influence of the length of the alkyl chain in the cation on the logarithm of the octanol–water partition coefficient (log *K*_OW_) of the products with bromide (**1**–**5**), 2,4-D (**6**–**10**), or MCPA (**11**–**15**) anions.

The log *K*_OW_ of the synthesized products (**1**–**15**) was assessed at a concentration equal to that of greenhouse experiments (preliminary test—400 g of the a.i. per ha). The results, presented in Figure 3 (and in Table S29, SI), confirmed that log *K*_OW_ values of the synthesized salt with herbicidal anions (**6**–**15**) occur in a range between 0 and 3, established as an “environmental safety zone.” The values noted for 2,4-D-based salts occurred in a range of 1.03 for **6** to 2.23 for **10**, while for salts with MCPA anions, they varied from 0.32 for **15** to 1.61 for **13**. Therefore, we can assume that the risk of their migration into groundwater after application is significantly reduced compared to potassium/sodium salts of phenoxy acids that are their most popular commercial forms with log *K*_OW_ amounting to approximately −1.5.^38^

According to Figure 3, in the case of products with bromide (**1**–**5**) and 2,4-D anion (**6**–**10**), the elongation of the alkyl group led to an increase in the log *K*_OW_. This phenomenon can be explained by the increasing hydrophobicity of the molecule as the share of the nonpolar part of the molecule increases.^39^ However, this relationship is less pronounced for compounds with longer chains.^40^ In effect, within the two homologous series (**1**–**5** and **6**–**10**), the extension of alkyl from C_14_ (tetradecyl) to C_18_ (octadecyl) had an almost inconsiderable impact on log *K*_OW_ values. Interestingly, in the case of products comprising MCPA as anions (**11**–**15**), the elongation of the alkyl above C_14_ (tetradecyl) resulted in the enhancement of their affinity for the polar phase. Two salts comprising the MCPA anion (**14** and **15**) possessed lower log *K*_OW_ values (0.84 and 0.32, respectively) than salt **13** (1.61). We can assume that the presence of MCPA as the anion uniquely affects the intramolecular/intermolecular interactions in these molecules, which alters their affinity for polar and nonpolar phases. However, further experiments are required to fully resolve the nature of this unexpected behavior.

Compounds with very high lipophilicity (particularly with log *K*_OW_ higher than 5), due to negligible solubility in water, are very persistent in the environment and have long lifetimes of biodegradation. For example, dichlorodiphenyltrichloroethane (DDT), widely applied as an insecticide between the 40 and 80 s of the XX century, possesses a log *K*_OW_ amounting to approx. 6.5 and, in effect, is still being detected in various agricultural soils around the world.^41^ However, the gathered data indicate that the possibility of bioaccumulation of the obtained derivatives of phenoxy acids (**6**–**15**) in the environment is also very low—their log *K*_OW_ values are much lower and do not exceed the value of 2.3. Furthermore, it is also extremely beneficial that the utilized reactants (nicotinamide, 2,4-D, and MCPA) are known to be susceptible to biodegradation; however, depending on the various conditions, biodegradation may occur within a few days or even months.^42,43^

### Volatility

The off-site movement of herbicides, occurring due to their volatilization after application, is known as the major issue that poses a serious risk of poisoning to living organisms and may also cause significant damage to nontolerant crops or other plants.^44^ According to reports from 2017, the southern states of the United States had to struggle with the drift of a new herbicidal formulation that was advertised as “nonvolatile”, which caused substantial losses in neighboring cultivated plants.^45^

Research aimed at the evaluation of the volatility of new ionic forms of herbicidally active substances was taken into consideration from a few different perspectives. Initially, the authors provided thermal stability data acquired *via* thermogravimetric analysis, such as the temperature of decomposition of 5% of the tested compound.^39^ However, it was quickly noted that there is no relation between the volatility of a substance and this parameter, which is more likely associated with the degradation of the particular functional groups to volatile byproducts. In effect, the second method, based on the determination of the weight loss of the sample after heating the sample at 75 °C for 12 h, was proposed.^46^ Nonetheless, recent reports revealed that the following approach is misleading and highly inaccurate because, in addition to the tested compound, other substances, such as impurities, solvents, or absorbed water, can also be responsible for the mass loss. Furthermore, it has been demonstrated that some herbicides, such as sulfonylureas, are highly unstable upon heating at 75 °C and decompose almost entirely with minimal mass loss (ca. 1–4%).^19^

Therefore, in the framework of this study, we developed a new method that allows for a more precise assessment of the potential of the obtained compound for volatilization. In the utilized approach, air is passed over the surface of an aqueous solution of a given herbicide (thermostated at 40 °C) and then introduced into the receiving scrubber. Analysis of the solutions in the receiving scrubber *via* UV spectrometry enabled the determination of the content of 2,4-D and MCPA herbicides that evaporated from the initial solution (see Figure S59 in the SI).

The obtained results, provided in Table 2, indicate that 2,4-D and MCPA have a potential for volatilization, and their concentrations in the receiving scrubber were equal to 1363.8 and 730.7 ppb, respectively. In contrast, the analyzed products **6**, **10**, **11**, and **15** exhibited negligible volatility, and for all of them, the herbicide content in the receiving scrubber was below the limit of detection (LOD ≈ 5 ppb). Because dimethylammonium cations are widely used as one of the most common organic counterions in many commercial preparations,^47^ dimethylammonium salts of 2,4-D and MCPA were also tested.

**Table 2:** Volatility of Selected Products (**6**, **10**, **11**, and **15**) from the Spray Solutions Compared to Phenoxy Acids in the Form of Free Acid and Dimethylammonium Salt

salt	anion	volatility (ppb)
**6**	2,4-D	<LOD^a^
**10**		<LOD^a^
dimethylammonium salt of 2,4-D		18.8
2,4-D in the form of free acid		1363.8
**11**	MCPA	<LOD^b^
**15**		<LOD^b^
dimethylammonium salt of MCPA		80.8
MCPA in the form of free acid		730.7

a LOD_2,4-D_ = 5.05 ppb.

b LOD_MCPA_ = 5.38 ppb.

**Scheme 2:**
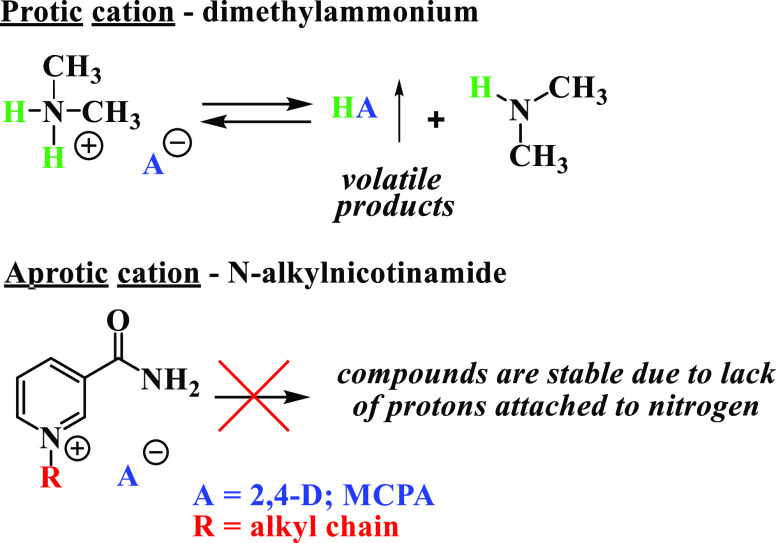
Differences in the Potential for Volatility between Quaternary Salts Comprising Protic and Aprotic Cations

As shown in Table 2, these two salts exhibited volatility multiple-fold lower than that of the parent phenoxy acids. However, due to the protic nature of the dimethylammonium cation (as demonstrated in Scheme 2), the obtained values reached 18.8 and 80.8 ppb for 2,4-D and MCPA salts, respectively. This means that the protonated salts of both herbicidal acids have volatility at least 3.7-fold higher (2,4-D) or even 15-fold higher (MCPA) compared to the corresponding *N*-alkylnicotinamide cation derivatives with the same anions. Such a difference can significantly affect the adverse effects of their application.

Thus, we can conclude that the benefits of the utilization of *N*-alkylnicotinamide cations are based on the fact that, in the case of quaternary ammonium cations, there is no risk of deprotonation and subsequent volatilization of either the amine or the acid.^48^ Therefore, synthesized salts do not pose a risk of spreading through the air, which constitutes a significant advantage over formulations containing protic ammonium cations (such as dimethylammonium) or free acids.

### Herbicidal Activity

#### Preliminary Test

**Figure 4:**
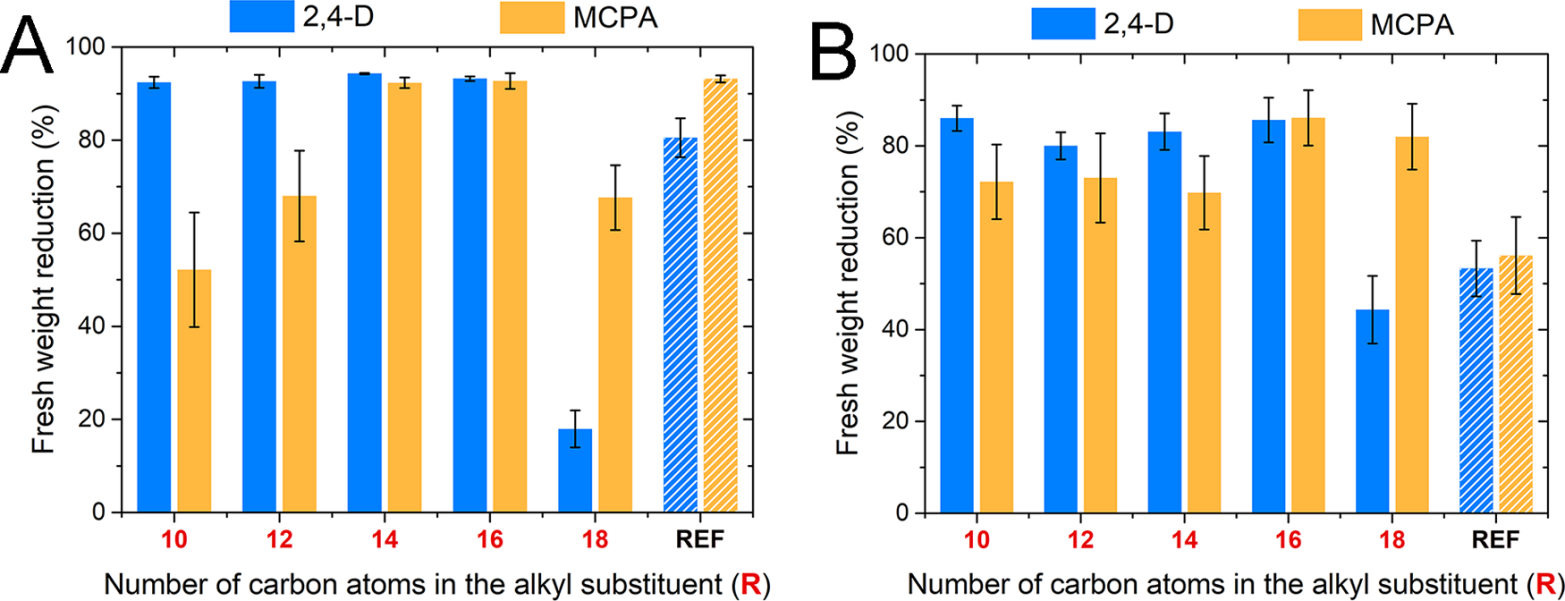
Influence of the alkyl chain length in the cation on the herbicidal efficacy of the obtained salts (**6**–**15**) toward cornflower (A) and oil-seed rape (B) compared to reference herbicides (**REF**).

The obtained products containing 2,4-D (**6**–**10**) and MCPA (**11**–**15**) anions were subjected to experiments aimed at determining their efficacy in inhibiting the development of unwanted vegetation. Cornflower and oil-seed rape, as examples of the most common weeds present in cultivated crops, were selected as test plants. In the preliminary test, all salts and reference (commercial) herbicides were applied at a dose corresponding to 400 g of active ingredient per hectare. The results demonstrated in Figure 4 (results with statistical analysis are provided in Table S30, SI) revealed that all of the synthesized products preserved the biological activity of the utilized anions. Moreover, due to the presence of a long alkyl chain in the cation (responsible for surface-active properties), the herbicidal efficacy of the majority of products toward both plants was similar to or greater than that of the reference herbicides (**REF**). In the case of cornflower (Figure 4A), no significant differences in the reduction in the fresh weight of plants were observed between commercial formulations and salts **6**–**9**, **13**, and **14**, allowing them to be described as equally effective. In the case of this plant, the effect of the length of the alkyl group was established to be significant only for salts with the MCPA anion. However, the exceptionally beneficial influence of the utilized cations was noted in tests conducted on plants of oil-seed rape (Figure 4B), where the majority of salts (**6**–**9**, **14**, and **15**) were found to be much more effective (72–86% of fresh weight reduction) than the reference preparations (53 and 56%). All of these differences were statistically significant. Interestingly, the data in Figure 4 indicate that salts comprising the longest substituent (C_18_) exhibit worse biological activity than salts with C_14_ or C_16_ groups. It should be noted that a similar trend was observed in the case of antimicrobial activity experiments, in which quaternary ammonium salts exhibited the optimal activity for C_12_–C_14_ alkyls. Further elongation of alkyl led to a substantial decrease in activity, which was later described as a “cutoff” effect and explained as the result of too great hydrophobicity or limited solubility in water.^49^ Nevertheless, on the basis of the provided data, products with C_14_ (**8**, **13**) and C_16_ (**9**, **14**) alkyl groups exhibited excellent herbicidal efficiency toward both plants (≥82%); in the case of oil-seed rape, the level of activity was approximately 1.5-fold higher compared to the currently used herbicide formulations (**REF**). Therefore, C_14_–C_16_ can be established as the optimal length of the alkyl chain. Generally, phenoxy acid-based formulations require the use of adjuvants, which, due to different registration procedures, may be much more toxic than the active substance itself.^19^ Hence, the solution provided in the framework of this study allows us to reduce the amount of chemicals that can become a source of potential pollution to the environment.

Subsequently, to evaluate the effect of surface activity on herbicidal activity, the values of surface tension and contact angle of the spray solutions utilized in the preliminary test were determined (data are shown in Figure S60 in the SI). The mentioned parameters were not measured for **10** and **15** because these salts partially precipitated out of solution during analysis (which did not allow us to collect reliable data). Therefore, due to excessively high hydrophobicity, these salts fail to improve the permeation of the active ingredient through the biological membranes of the plant and meet the requirements of the elaborated research hypothesis. Based on the available literature, it can also be concluded that the synthesized salts were applied at concentrations exceeding the critical micellization concentration (CMC). No statistically significant differences were observed for the measured values of contact angle and surface tension, which occurred in a range of 45–50° and 33.9–37.4 mN m^–1^, respectively. It was also established that the structural differences between 2,4-D and MCPA exhibited an insignificant influence on both mentioned parameters. Nonetheless, it should be stressed that spray solutions of reference herbicides containing 2,4-D or MCPA were characterized by surface activity similar to pure water (approx. 72 mN m^–1^), which means that the *N*-alkylnicotinamide cation can act effectively as an adjuvant and clearly confirms the assumed research hypothesis. Moreover, the results collected for oil-seed rape revealed that the improvement in the wettability of the hydrophobic surface can lead to substantial enhancement in the herbicidal efficacy toward plants exhibiting lower sensitivity to the applied active ingredients, such as MCPA. In effect, the synthesized salts can be significantly more effective than commercial forms.^50^

#### Dose–Response Test

In the second experiment, *N*-hexadecylnicotinamide 4-chloro-2-methylphenoxyacetate (**14**) was selected as a representative of the group of the most effective salts to elucidate its dose–response relationship in comparison with the reference herbicide. In this study, the same plant species were utilized, while the dose of the active ingredient varied from 200 to 1000 g ha^–1^ (generally, phenoxy acids are applied in crop fields at doses corresponding to 600–900 g ha^–1^).

**Figure 5:**
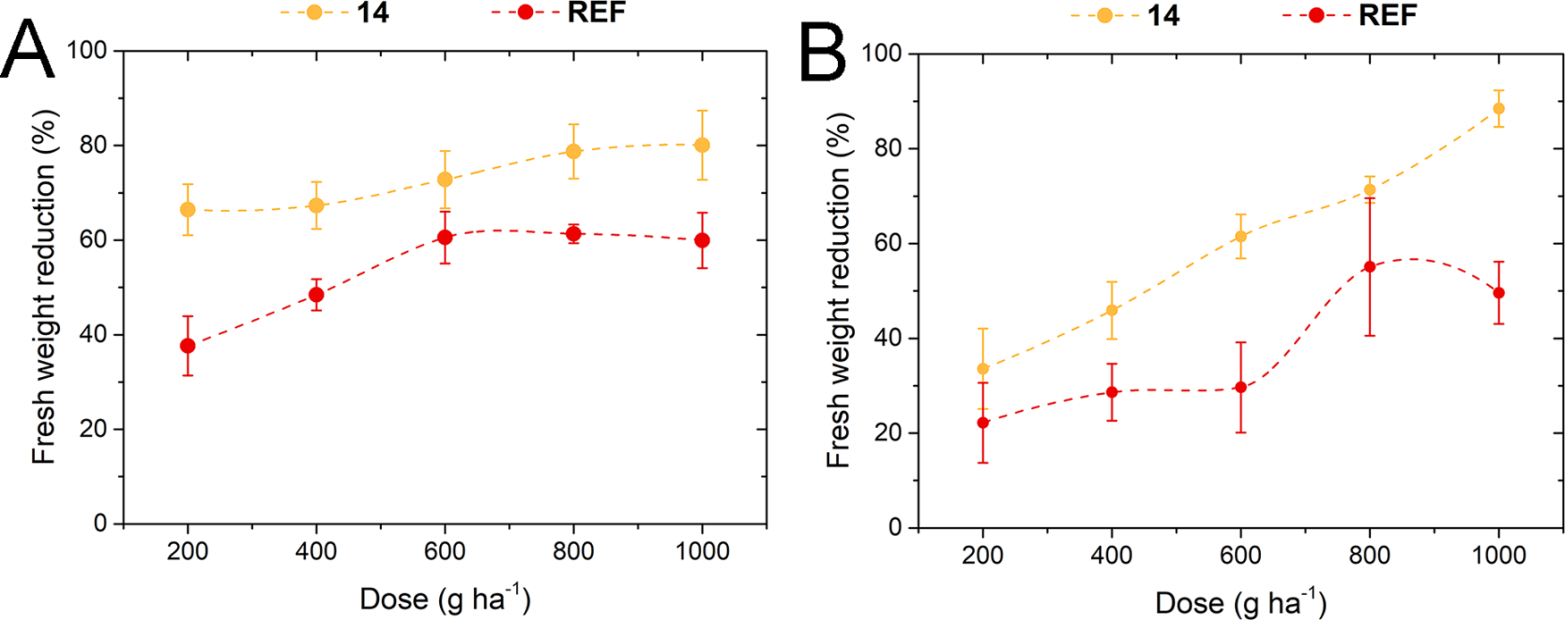
Relationship between applied dose and herbicidal activity for *N*-hexadecylnicotinamide cation and MCPA anion (**14**) and commercial formulation (**REF**) cornflower (A) and oil-seed rape (B).

The results, provided in Figure 5 (and in Table S31 in the SI), indicate that salt **14** was more effective toward both plants than the commercial formulation at almost all applied doses. The differences in efficacy between salt **14** and the reference herbicide (**REF**) toward cornflower and oil-seed rape varied from 12 to 29% and from 11 to 39%, respectively. The obtained results clearly indicate that, due to the introduction of *N*-hexadecylnicotinamide cation, it is possible to significantly reduce the amount of active ingredient without compromising the herbicidal activity of the formulation. In the test conducted on oil-seed rape plants, salt **14** applied at a dose of 400 g ha^–1^ showed similar activity to that of a commercial preparation at a dose twice as high (800 g ha^–1^), while in the test on cornflower plants, compound **14** at the lowest tested dose of 200 g ha^–1^ exhibited better effect than the reference herbicide used at the highest tested dose, i.e., 1000 g ha^–1^. In addition, the increase in the dose of salt **14** caused more significant damage to oil-seed rape than to cornflower. As presented in Figure 5B, the efficiency of **14** rose from approx. 34% for 200 g ha^–1^ to 89% for 1000 g ha^–1^. This test clearly confirmed the beneficial influence of the utilized amphiphilic cations on the improvement of compound performance and showed that they are good candidates for field trials, which will be conducted in the near future.

## Supporting Information Available

Impact of the solvent selection on the quaternization; UV, FT-IR, and NMR spectra of synthesized products; absorption maxima and molar absorptivities in UV spectra; absorption maxima in FT-IR spectra; analysis of ^1^H NMR spectra; solubility in 10 representative solvents; values of logarithm of octanol–water partition coefficient; scheme of an installation for determining the volatility; curves for determination of limit of detection; UV spectra of the solutions in the scrubber after the experiments; herbicidal activity; surface tension and contact angles of the tank mixes; and dose response (PDF)
