# Supplementary material for: Sustainable Design of New Ionic Forms of Vitamin B3 and Their Utilization as Plant Protection Agents
Source: J Agric Food Chem. 2022 Jun 29;70(27):8222–32. doi: 10.1021/acs.jafc.2c01807 (PMC9284545; doi:10.1021/acs.jafc.2c01807)
Supplement: Supplementary file 1 — jf2c01807_si_001.pdf [file jf2c01807_si_001.pdf]

ELECTRONIC SUPPORTING INFORMATION

Sustainable design of new ionic forms of vitamin B_3 and their utilization as plant protection agents

Witold Stachowiak, Damian Krystian Kaczmarek, Tomasz Rzemieniecki, Michał Niemczak*

Department of Chemical Technology, Poznan University of Technology, Poznan 60-965, Poland

* Corresponding author at: Poznan University of Technology, Berdychowo 4, 61-131 Poznan, Poland; Tel.: +48 616653681. E-mail: michal.niemczak@put.poznan.pl

Table S1. The impact of the solvent selection on the yield of nicotinamide quaternization and quality of the obtained product (**1**).

	**Dimethylformamide (DMF)**	**Tetrahydrofurane (THF)**	**Xylene**	***n*-propanol**	**Acetone**
**Classification according to CHEM21 selection guide**[a]	**Hazardous**	**Problematic**	**Problematic**	**Recommended**	**Recommended**
**Temperature [°C]**	80	66	139	97	56
**Reaction time [h]**	8	24	20	20	42
**Solvent volume [cm^3]**	10	20	20	20	20
**Mass of product [g]**	5.49	3.45	6.45	4.91	2.94
**Yield [%]**	80.0	49.7	94.0	71.6	42.9
**At the start, is the mixture homogeneous?**	Yes	No	No	Yes	No
**Benefits from replacement of DMF**		**Low**	**Moderate**	**High**	**Low**
**Justification**	The reaction rate is good, however, DMF is hazardous and reusing the solvent (B. P. 153 °C) requires a lot of energy.	The reaction rate is too low, the product contains significant amount of unreacted nicotinamide.	The reaction rate is good. Time of mixing can be shortened. High temperature causes formation of colored impurities.	The reaction rate is satisfactory. Moreover, the process can be shortened by using higher concentration of substrates.	The reaction rate is too low. The product consists mainly of unreacted nicotinamide.

[a] D. Prat, A.Wells, J. Hayler, H. Sneddon, C. R. McElroy, S. Abou-Shehadad, P. J. Dunn, *Green Chem.*, **2016**, 18, 288–296

GENERAL PROCEDURE: Initially, 20 mmol of nicotinamide (2.44 g) and 21 mmol of 1-bromodecane (5% molar excess, 4.64 g) were mixed (600 rpm) with selected solvent (table S1) at elevated temperature over a given period of time. Dimethylformamide, tetrahydrofurane and xylene were selected due to the fact that their plausible use as a quaternization medium was reported in literature. However, in order to increase the safety during the process (in the light of safety, health & environmental criteria) an attempt was made to utilize additionally non-expensive, fully renewable, easy biodegradable and safe solvents, like acetone or *n*-propanol. After mixing of reactants, the contents of the reactor were cooled, and the sediment was subsequently filtered off and washed three times with ethyl acetate or acetone. The product was finally dried under vacuum (5–10 mbar) at 50 °C for 24 h.

NOTE: After initial trials, *n*-propanol was chosen as the most beneficial replacement for dimethylformamide (DMF) due to its “greeness”, low cost and easy recovery *via* distillation. However, S_N2 type reactions run slower in polar protic solvents than in aprotic ones due to hydrogen bonding between hydrogen and nucleophile. In effect, the concentration of substrates was raised 2 times to 2M, which allowed for the reduction of the reaction time to less than 10 h, which is comparable to the reaction time reported in literature for the process carried out in DMF.

Figure S1. *UV spectrum of N-decylonicotinamide bromide (**1**).*

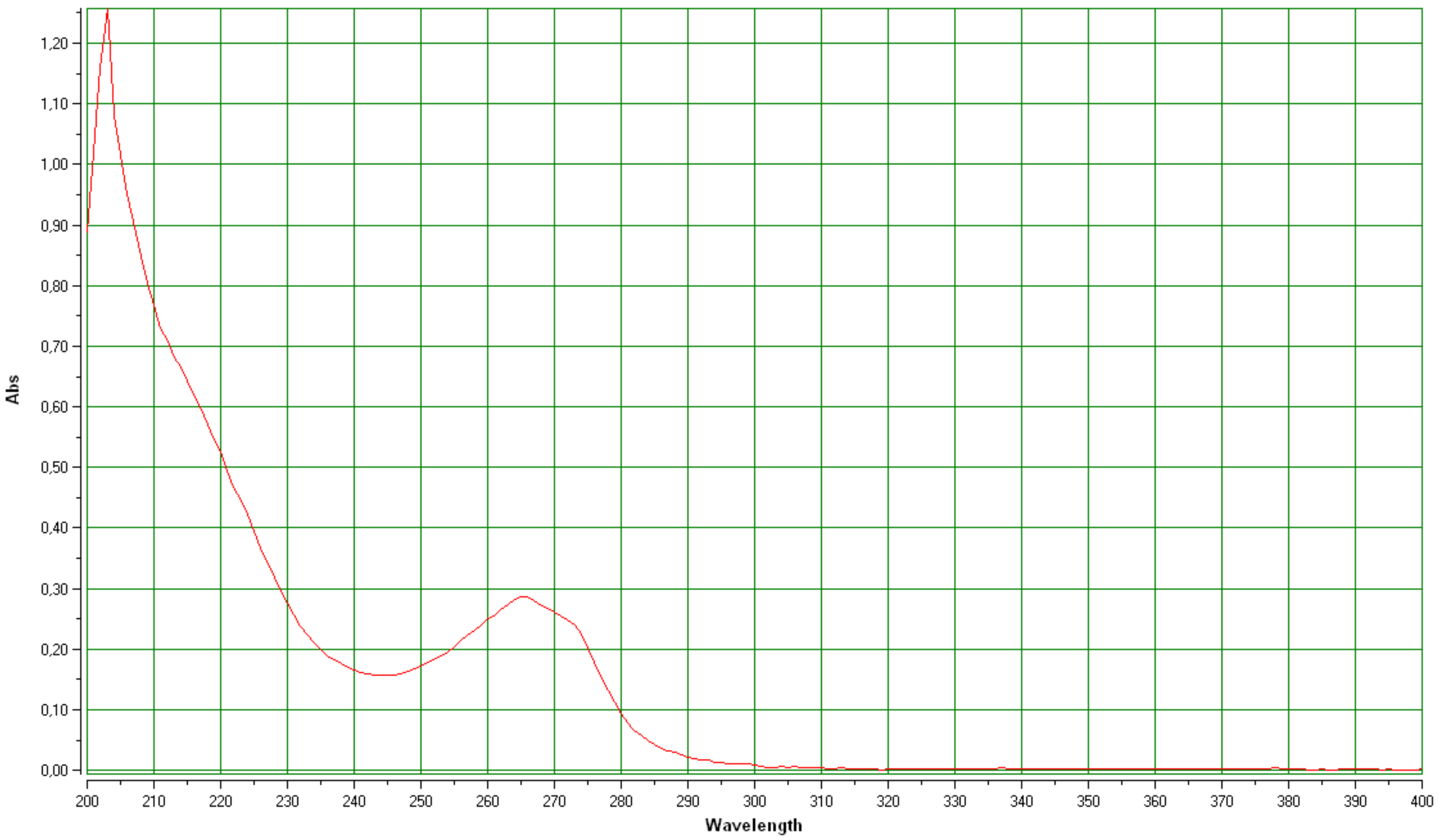

***Figure S2**. FT-IR spectrum of N-decylonicotinamide bromide (**1**).*

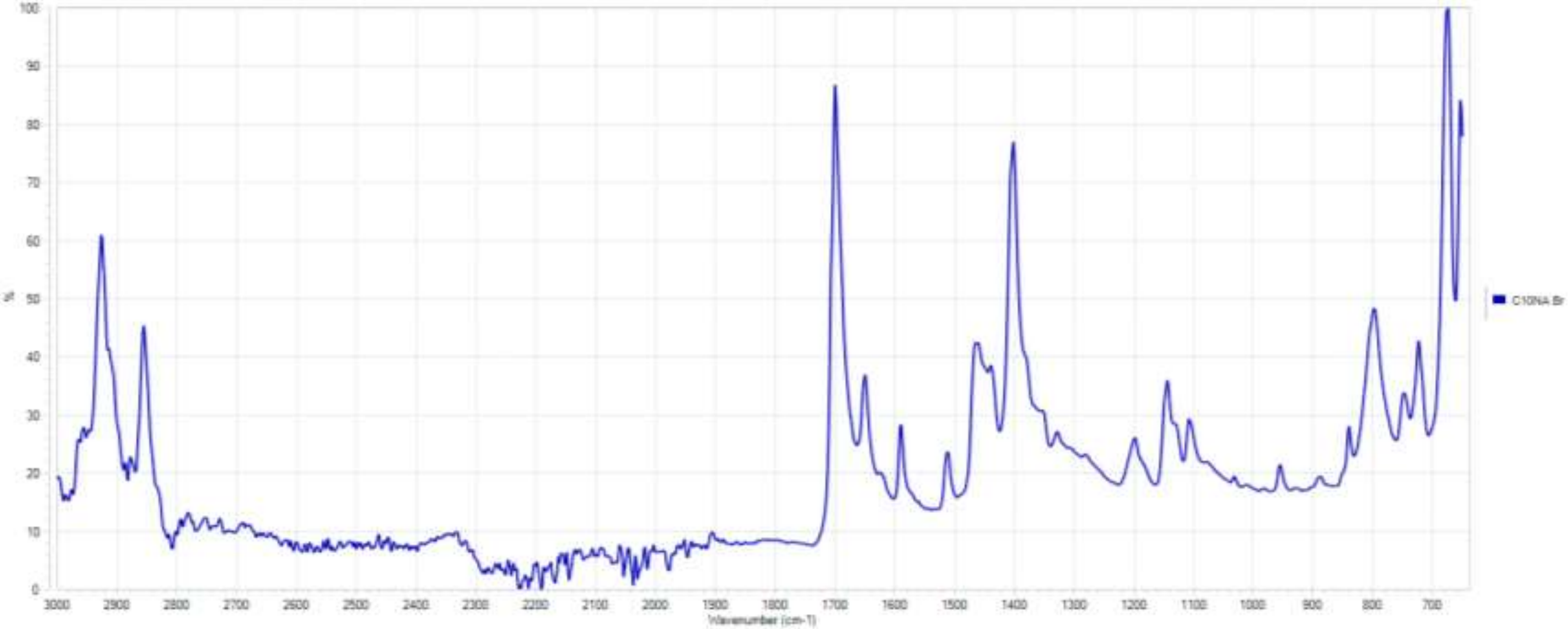

Figure S3. *^{1}H NMR spectrum of N-decylonicotinamide bromide (**1**).*

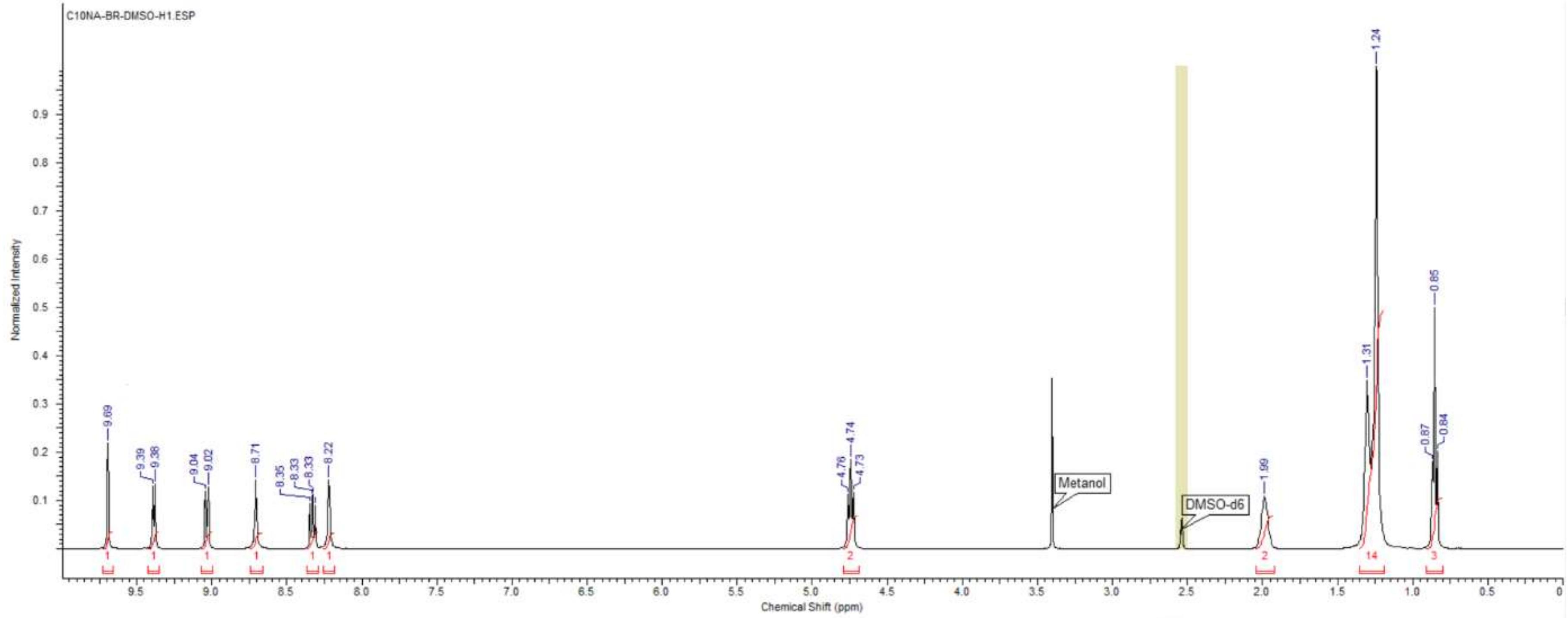

Figure S4. *^{13}C NMR spectrum of N-decylonicotinamide bromide (**1**).*

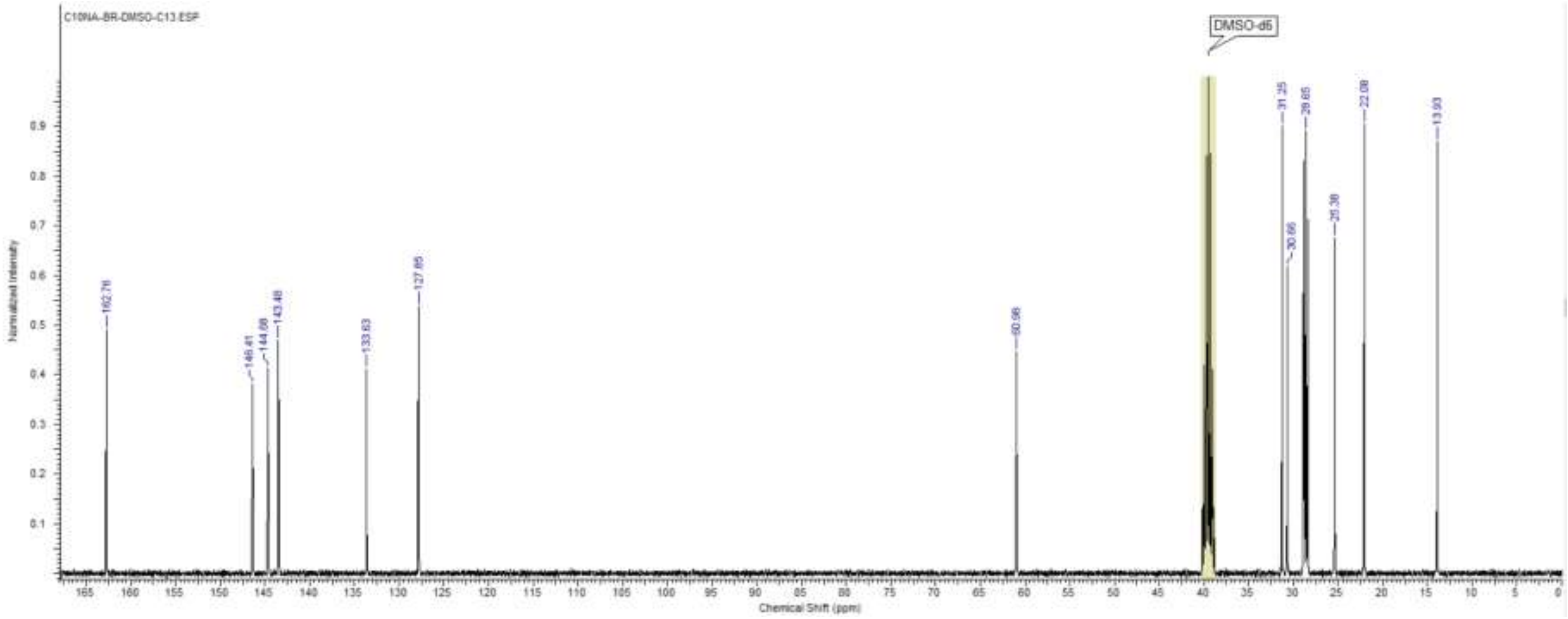

__Figure S5__. UV spectrum of N-dodecylonicotinamide bromide (__2__).

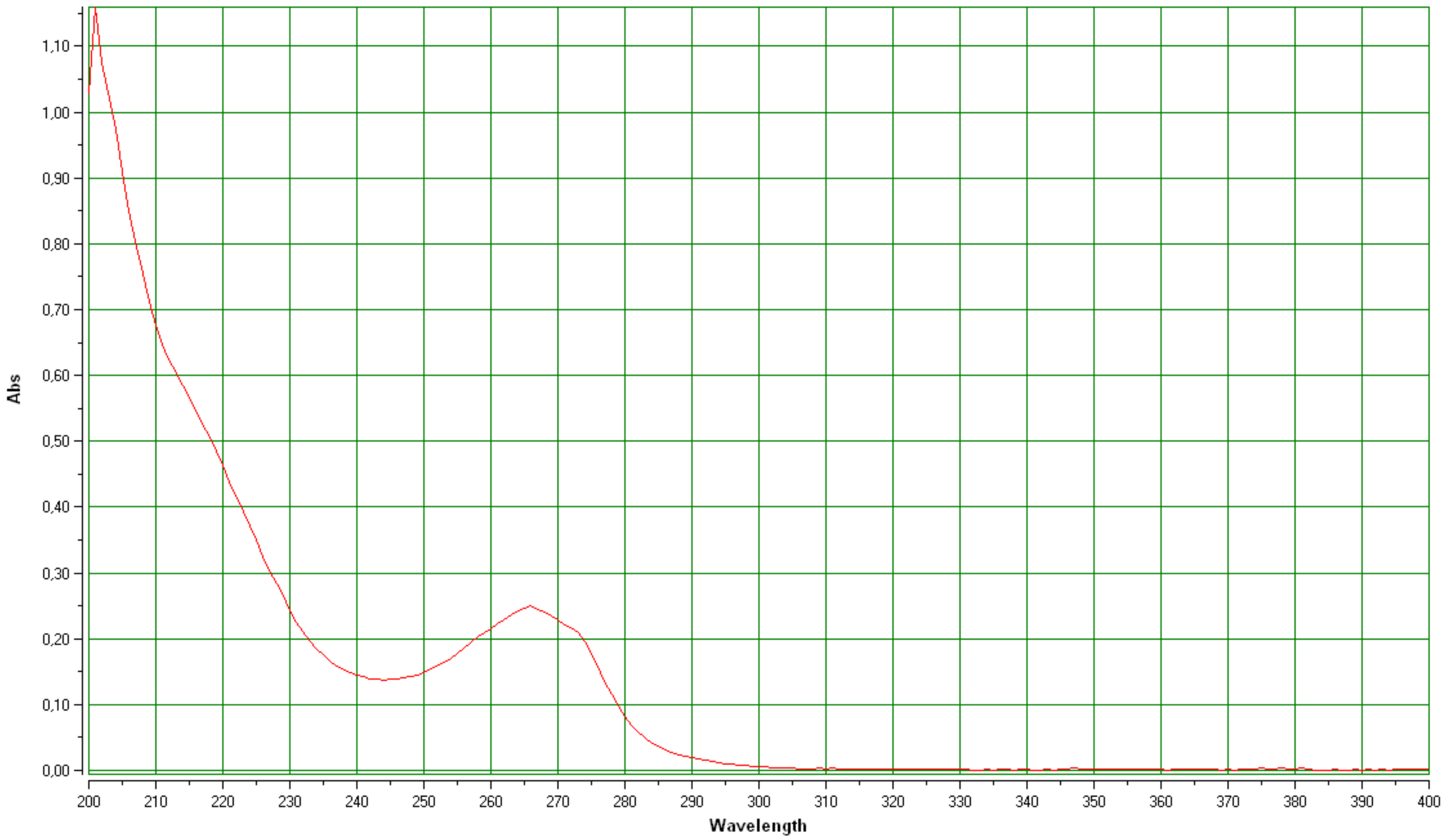

Figure S6. *FT-IR spectrum of N-dodecylonicotinamide bromide (**2**).*

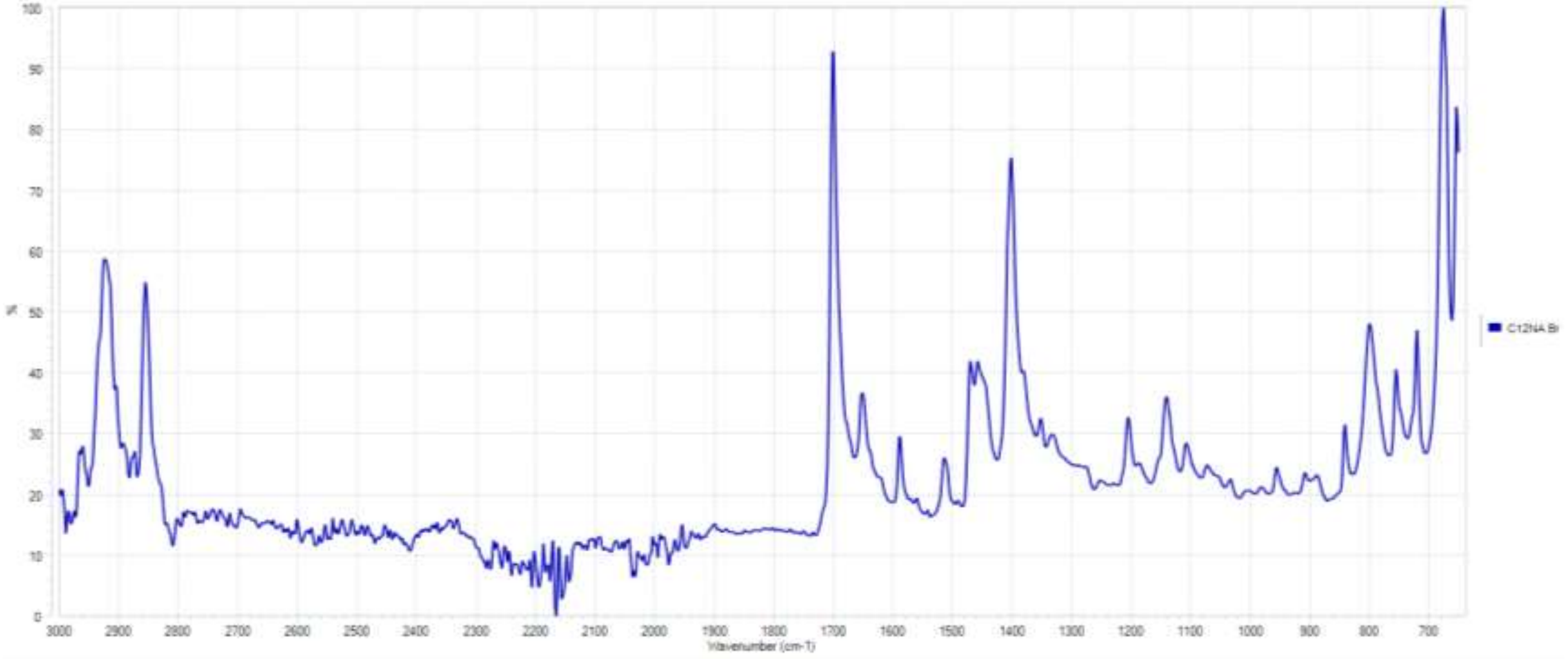

***Figure S7**. UV spectrum of N-tetradecylonicotinamide bromide (**3**).*

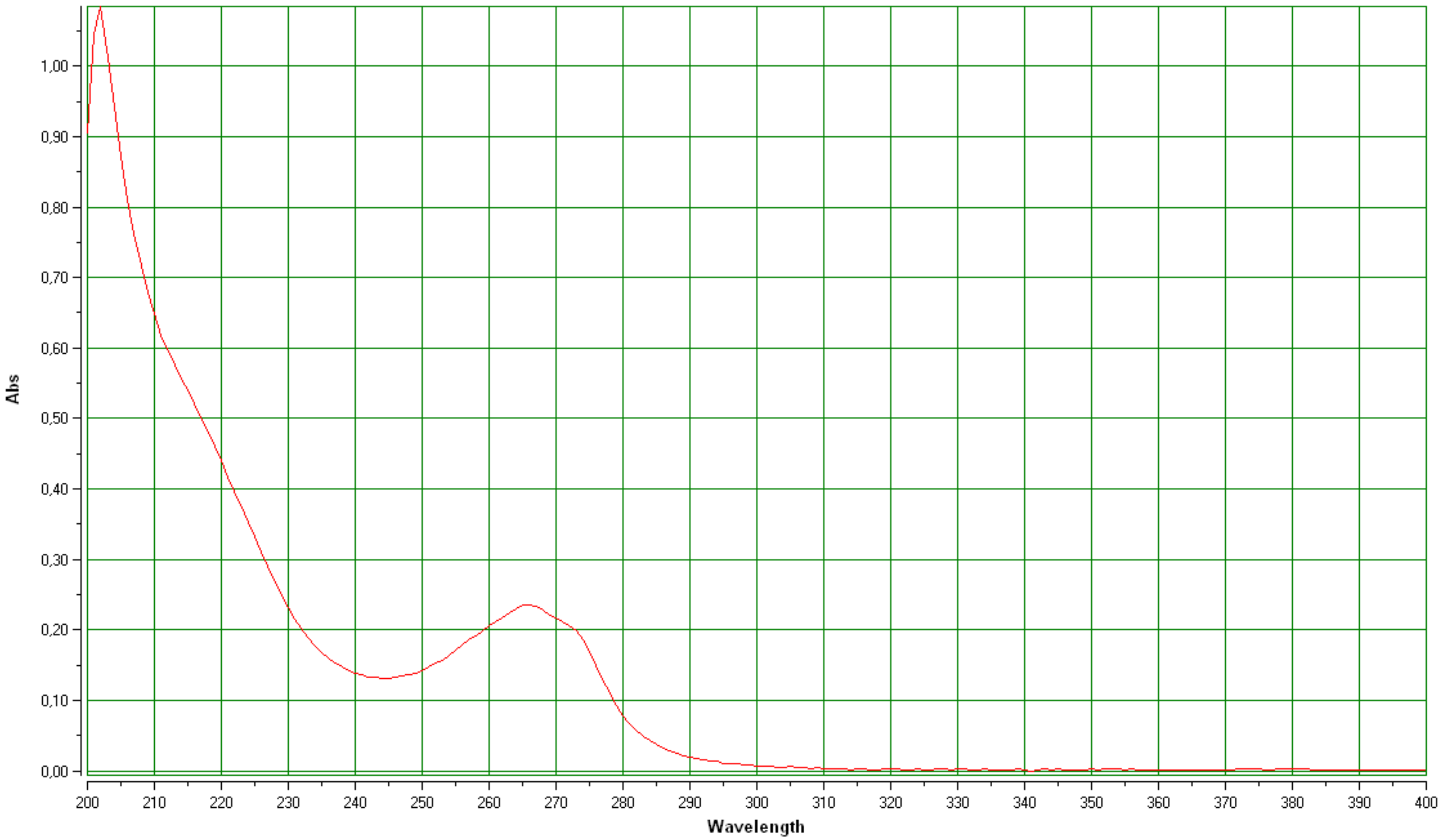

Figure S8. *FT-IR spectrum of N-tetradecylonicotinamide bromide (**3**).*

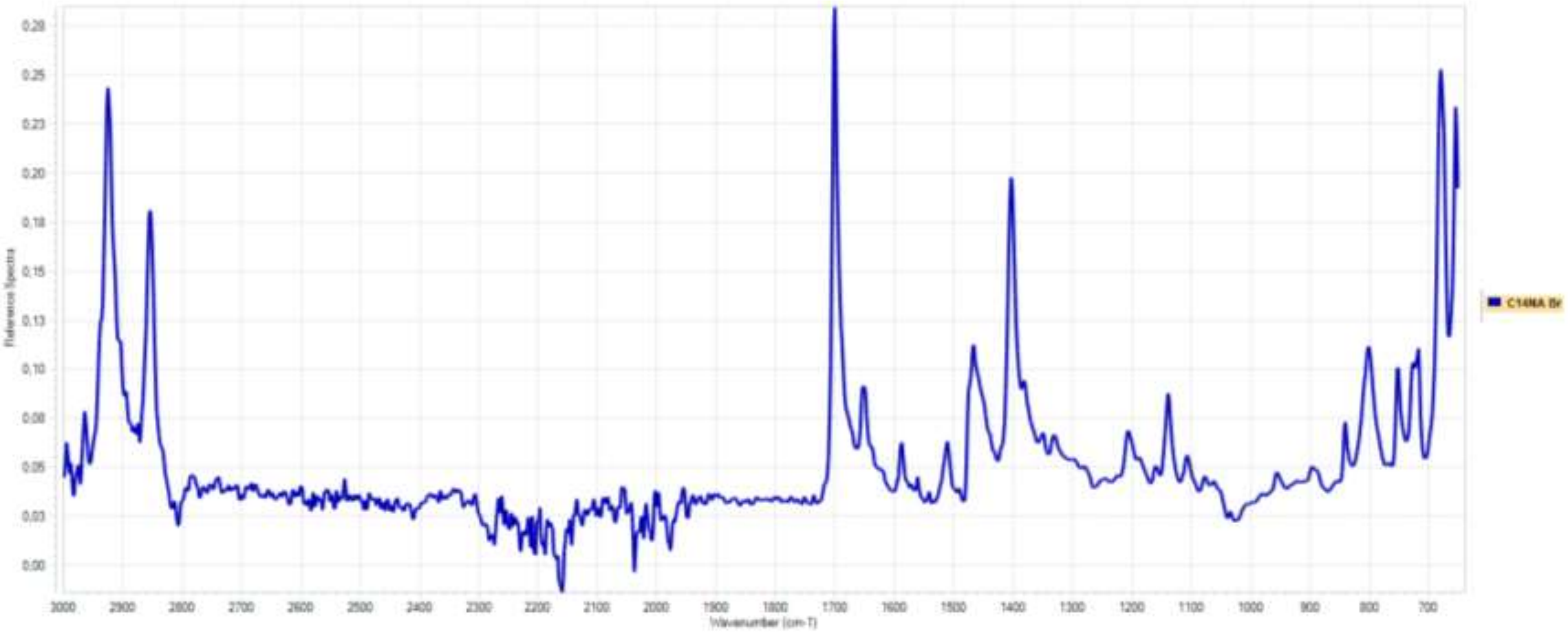

Figure S9. *^{1}H NMR spectrum of N-tetradecylonicotinamide bromide (**3**).*

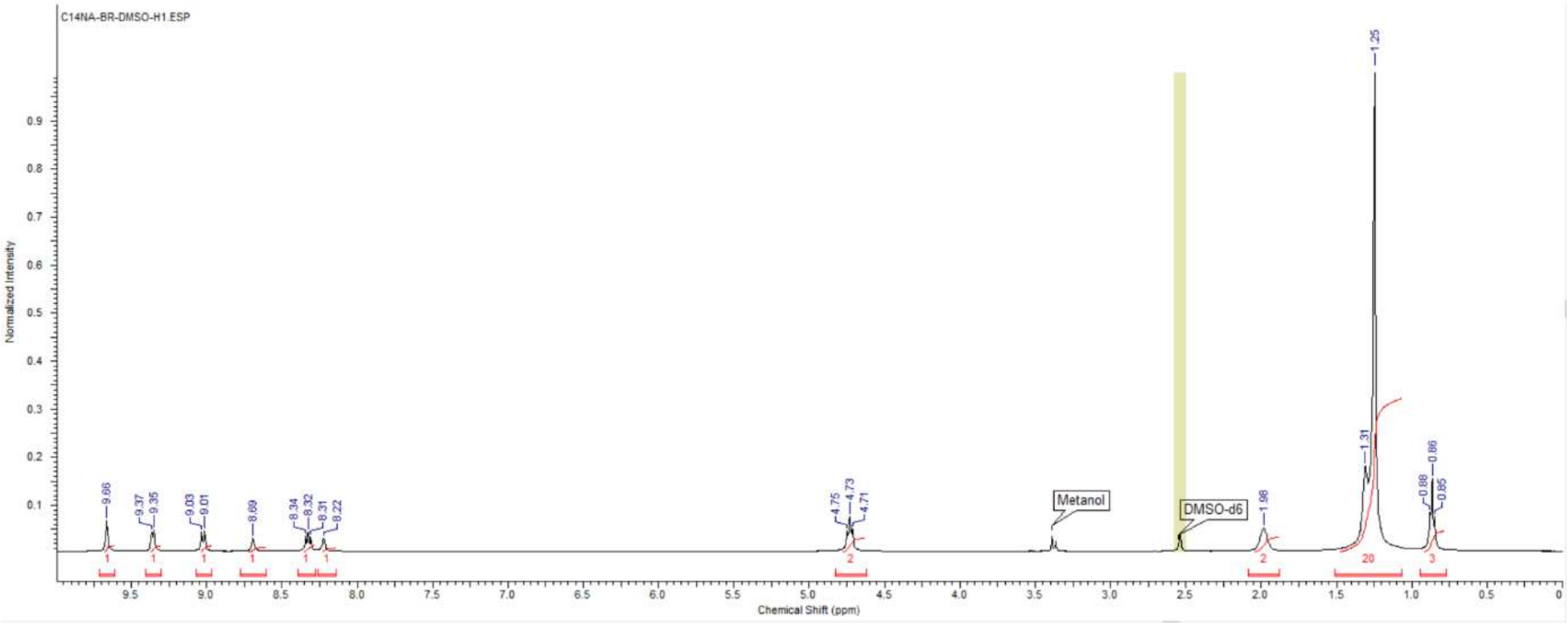

Figure S10. ^{13}C NMR spectrum of N-tetradecylonicotinamide bromide (**3**).

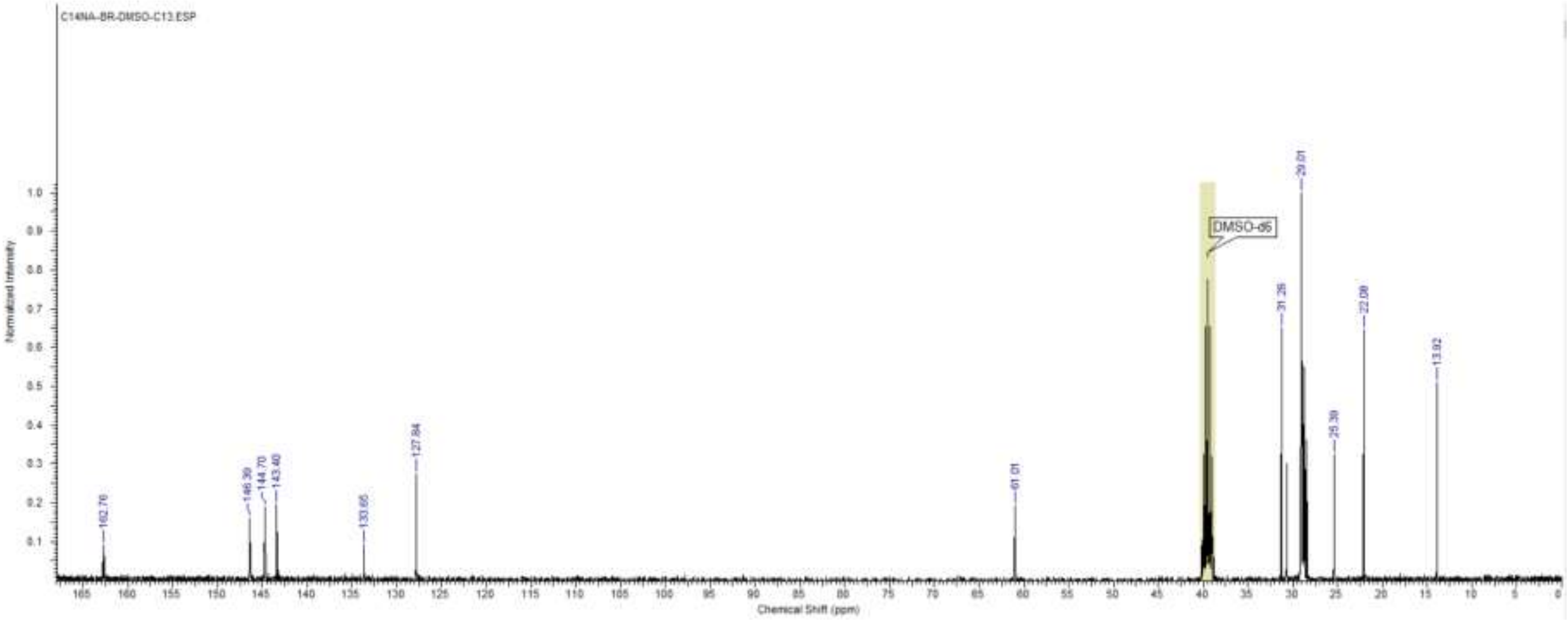

Figure S11. *UV spectrum of N-hexadecylonicotinamide bromide (**4**).*

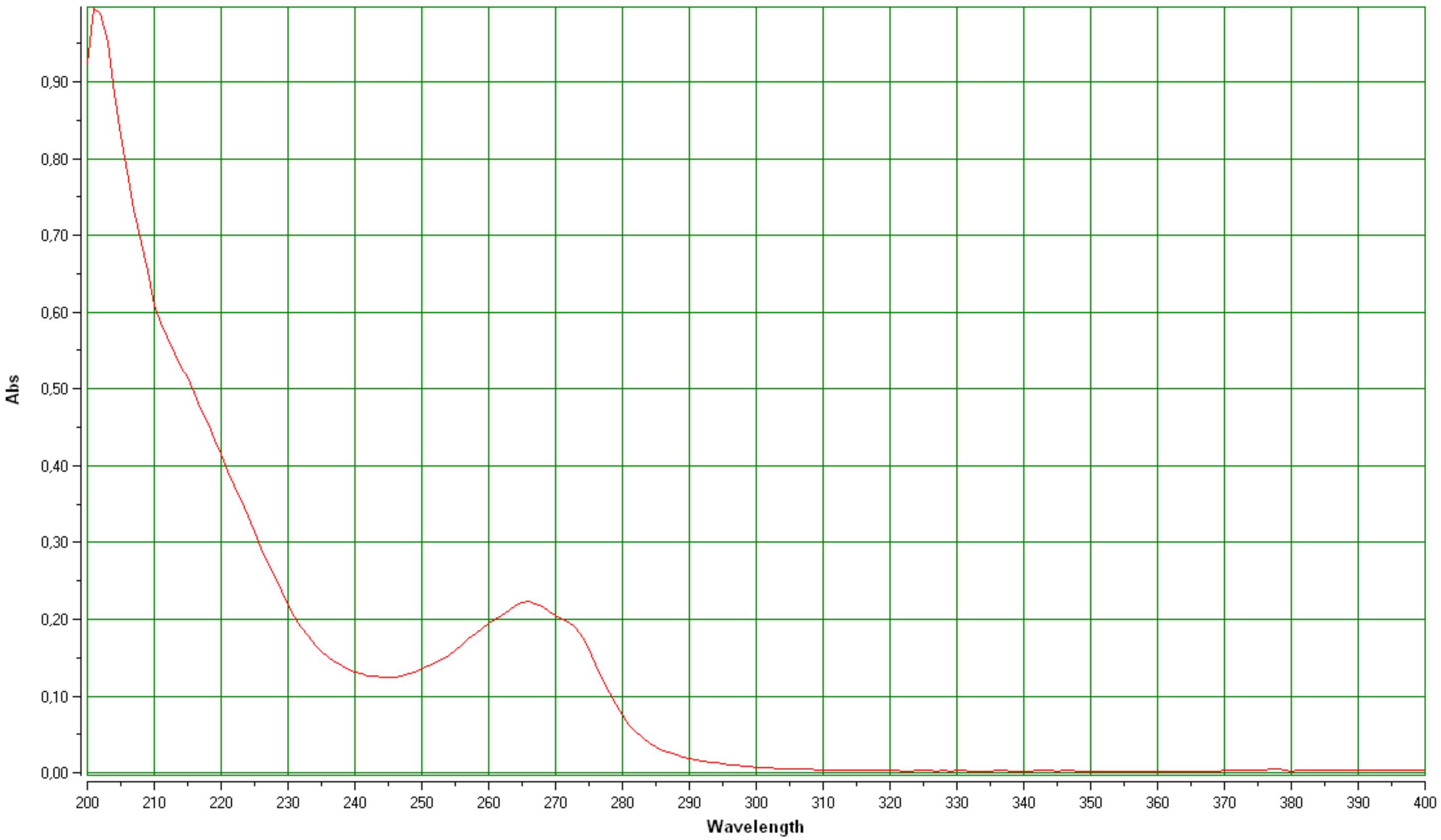

Figure S12. *FT-IR spectrum of N-hexadecylonicotinamide bromide (**4**).*

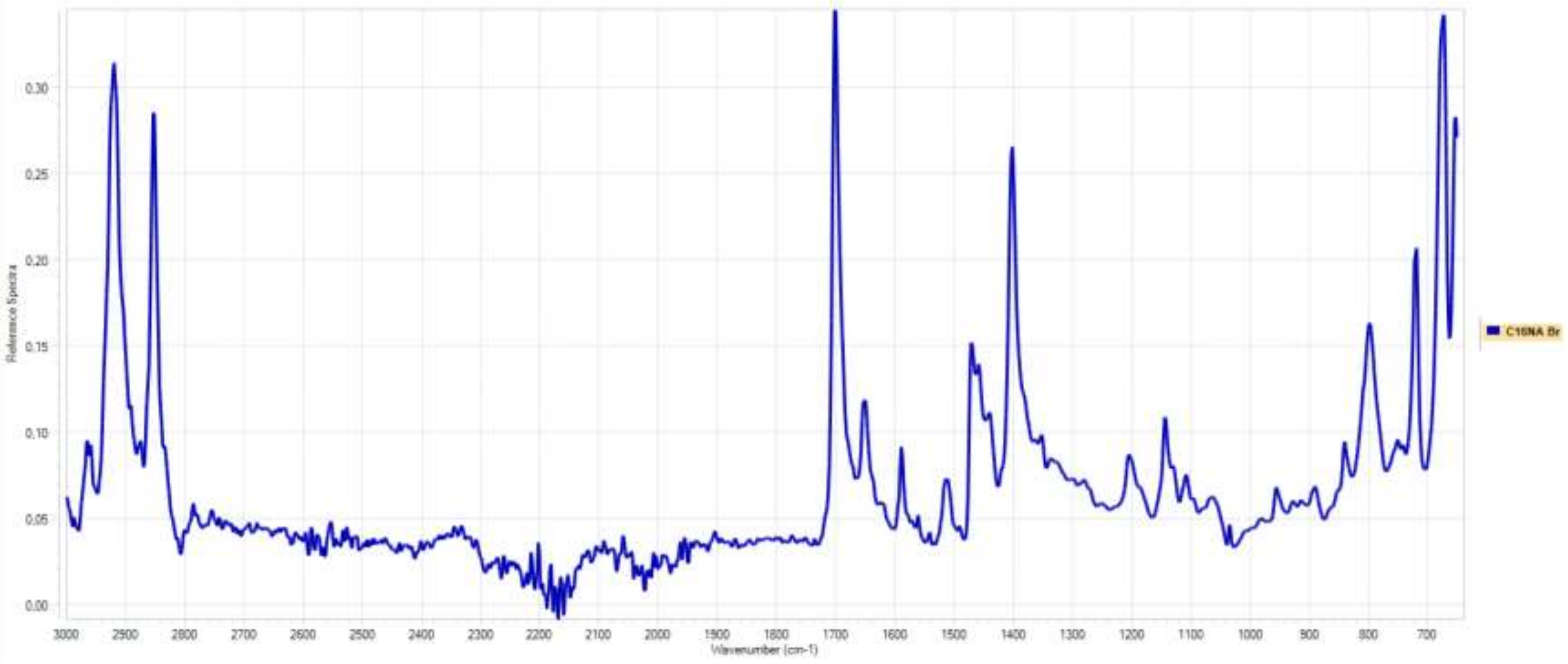

Figure S13. *UV spectrum of N-octadecylonicotinamide bromide (**5**).*

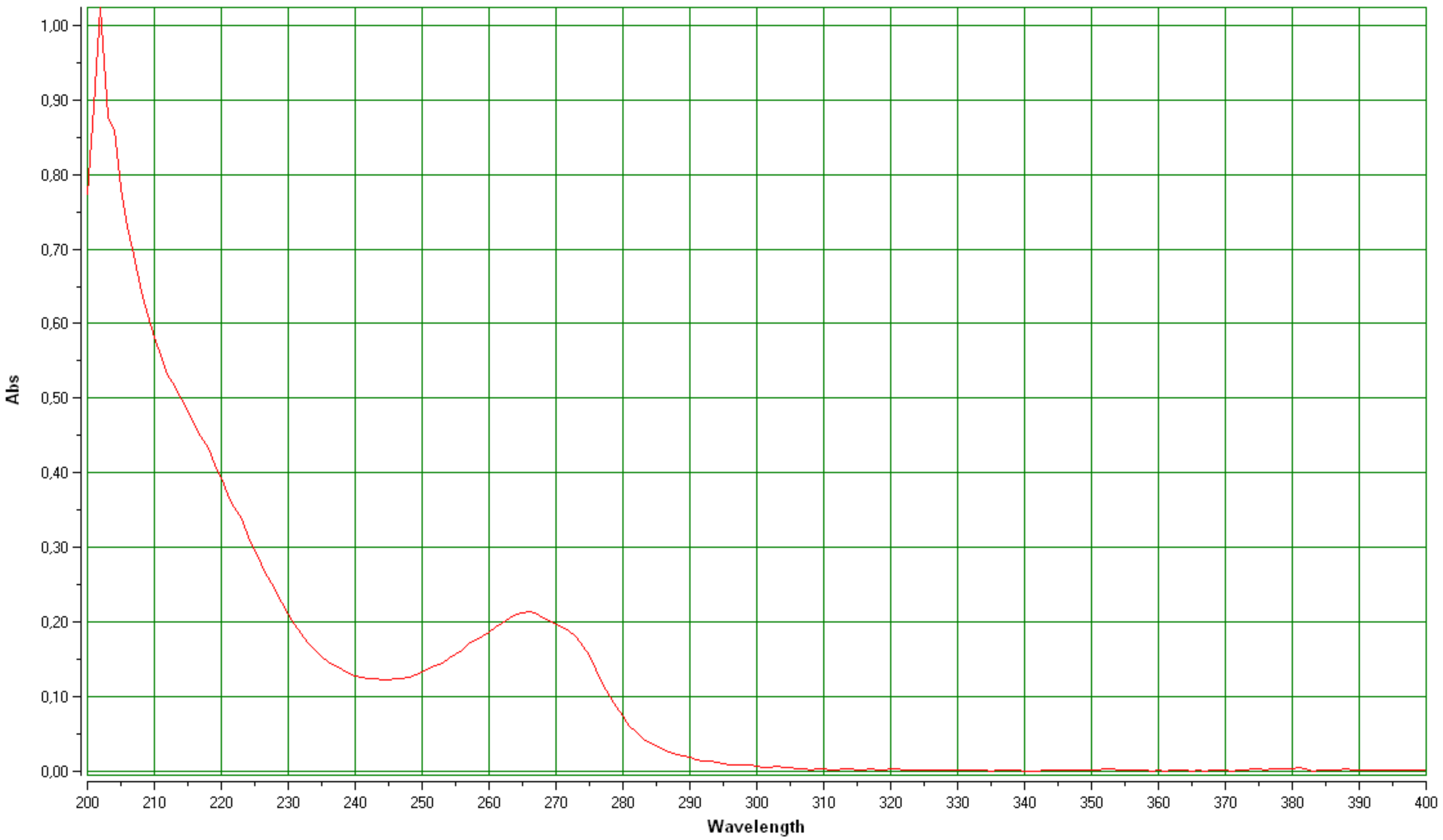

Figure S14. *FT-IR spectrum of N-octadecylonicotinamide bromide (**5**).*

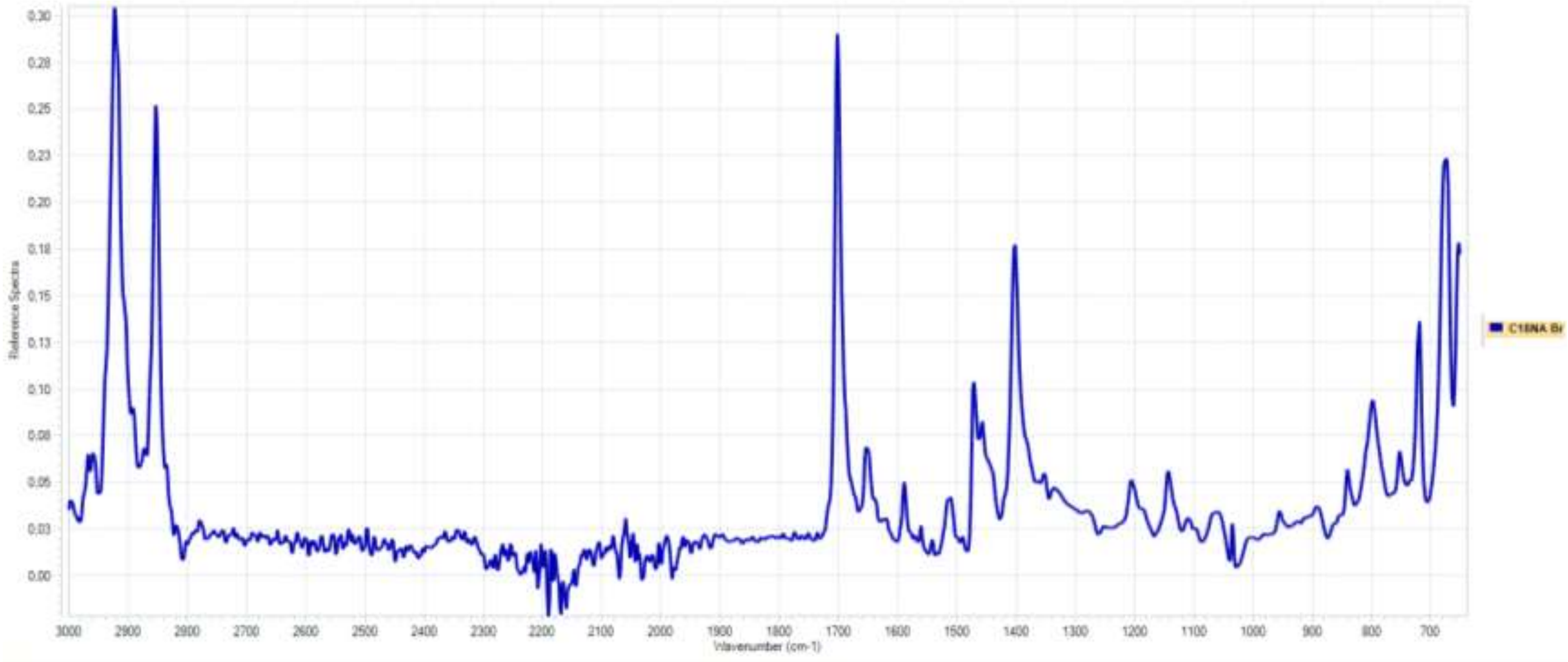

Figure S15. *UV spectrum of N-decylonicotinamide 2,4-dichlorophenoxyacetate* (***6***).

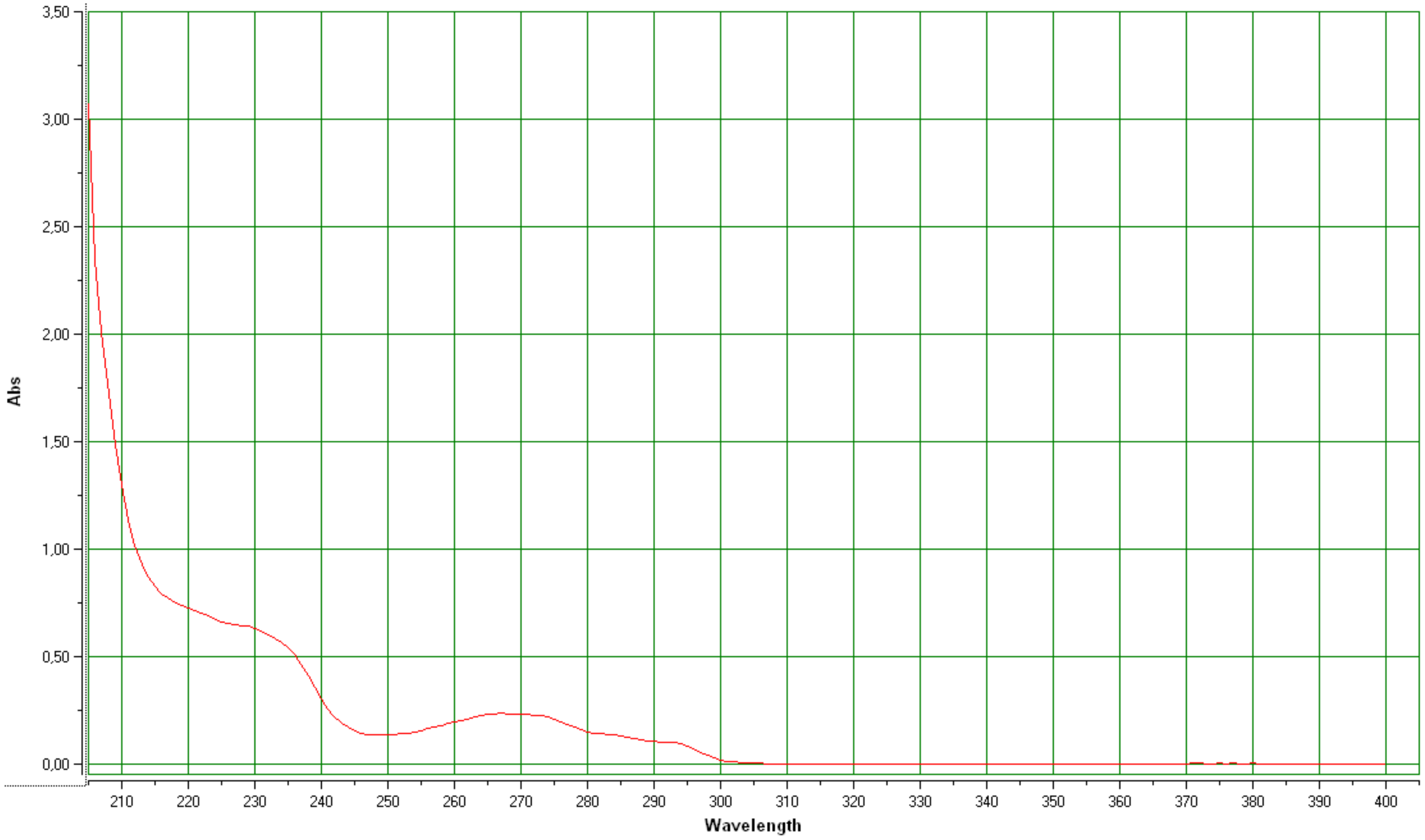

Figure S16. *FT-IR spectrum of N-decylonicotinamide 2,4-dichlorophenoxyacetate (6).*

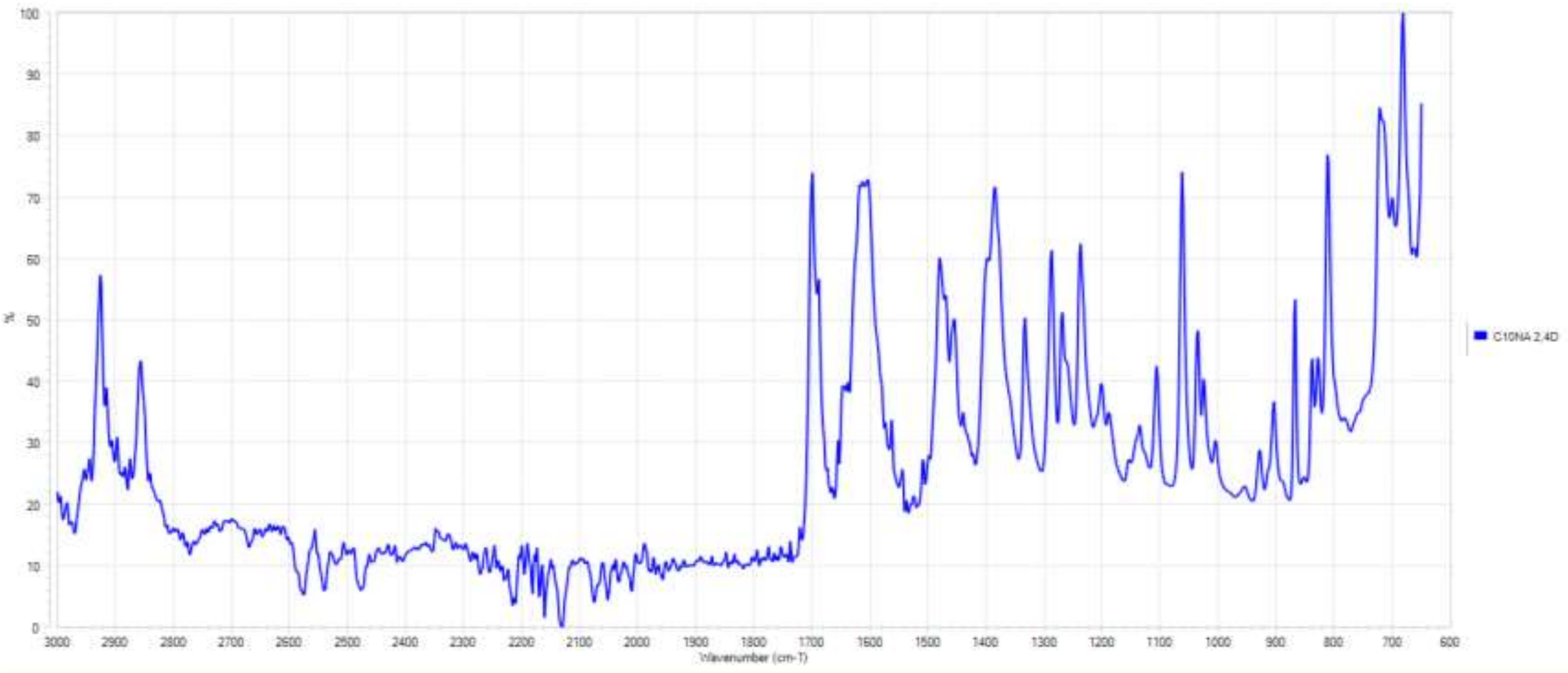

Figure S17. *^{1}H NMR spectrum of N-decylonicotinamide 2,4-dichlorophenoxyacetate (**6**).*

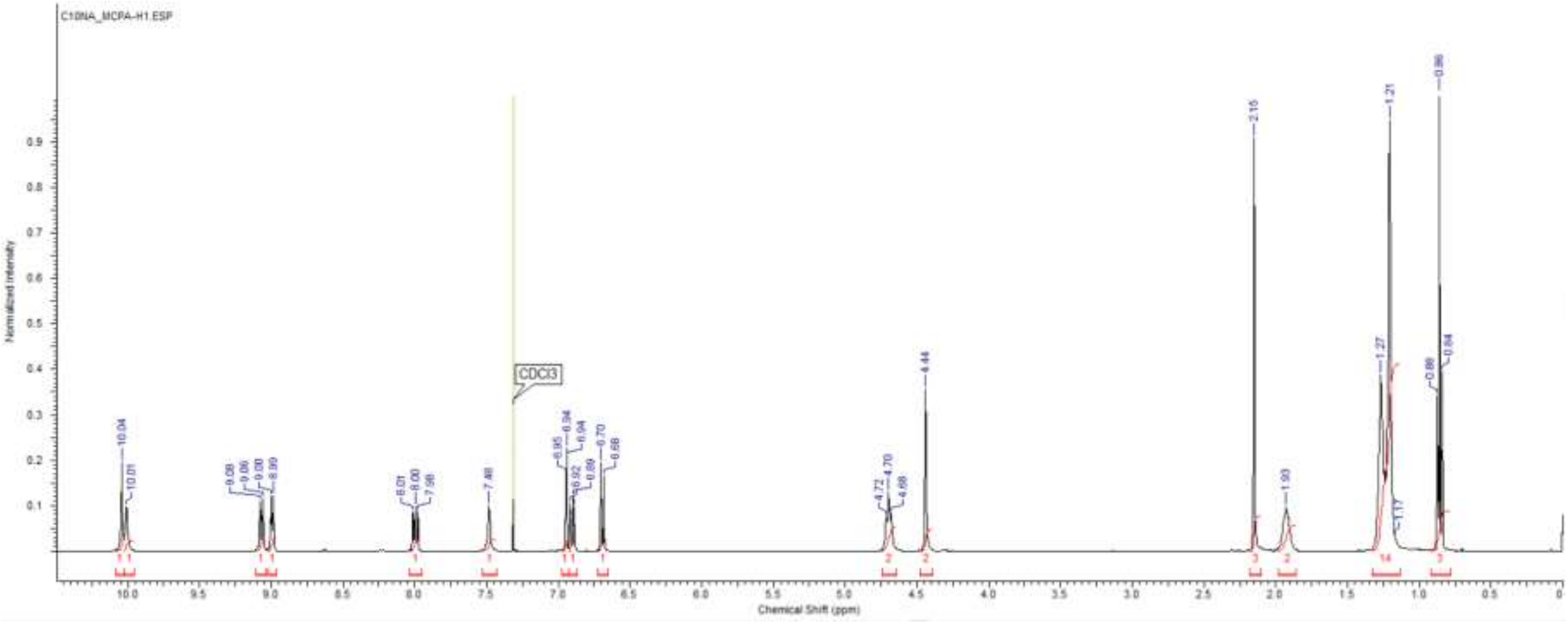

Figure S18. *^{13}C NMR spectrum of N-decylonicotinamide 2,4-dichlorophenoxyacetate (**6**).*

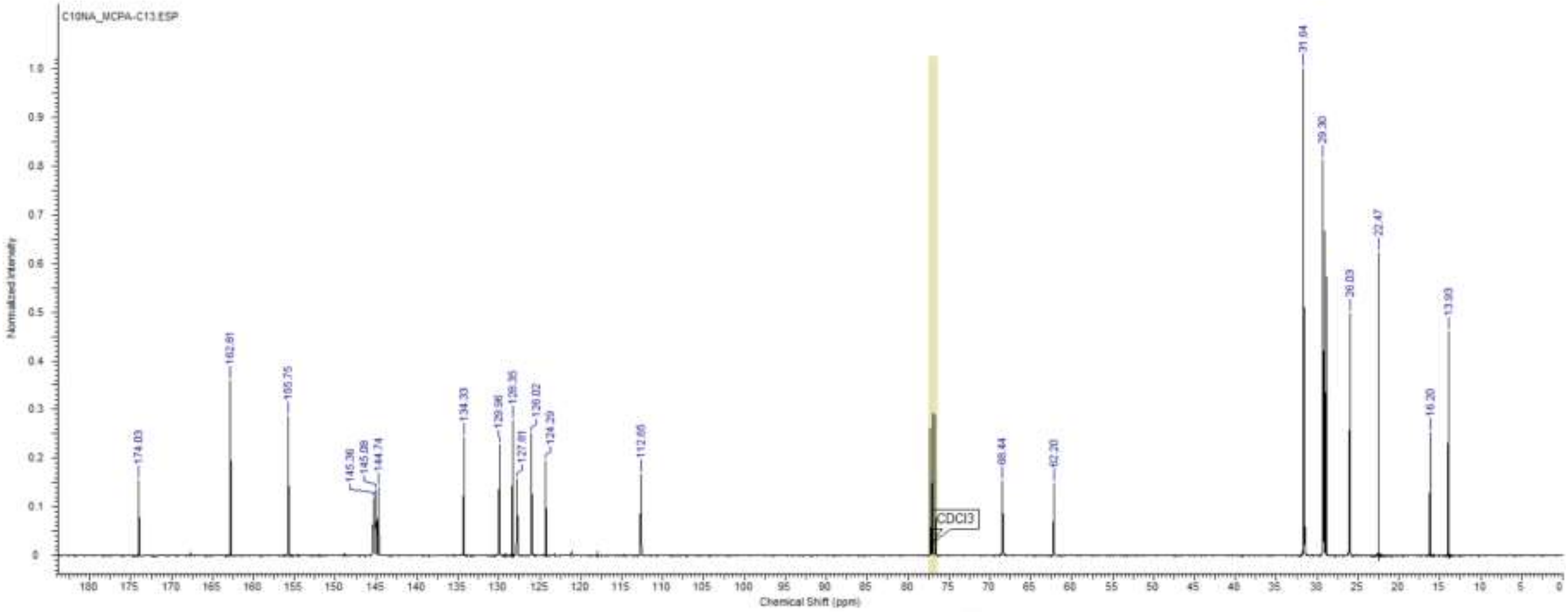

Figure S19. *UV spectrum of N-dodecylonicotinamide 2,4-dichlorophenoxyacetate (**7**).*

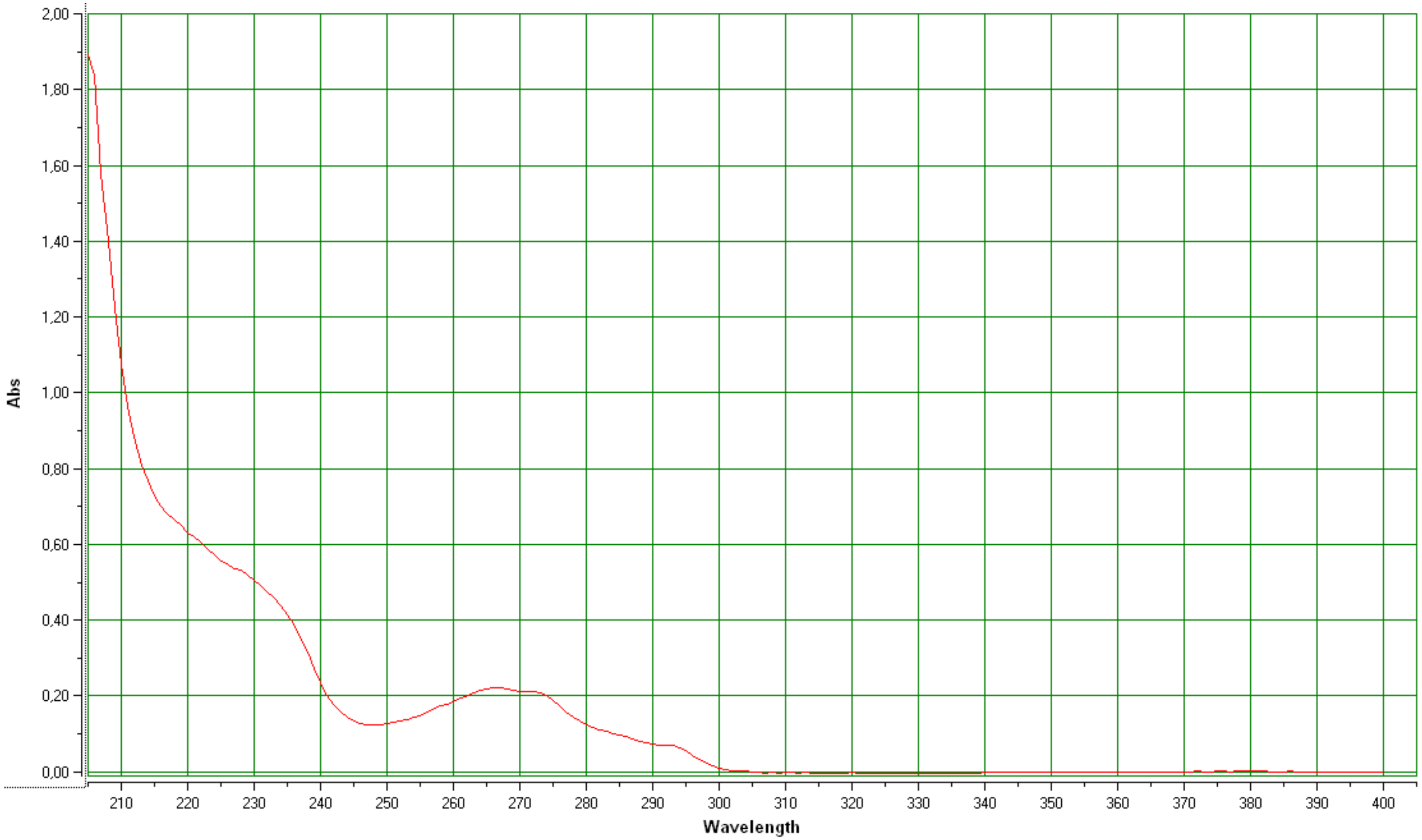

Figure S20. *FT-IR spectrum of N-dodecylonicotinamide 2,4-dichlorophenoxyacetate (**7**).*

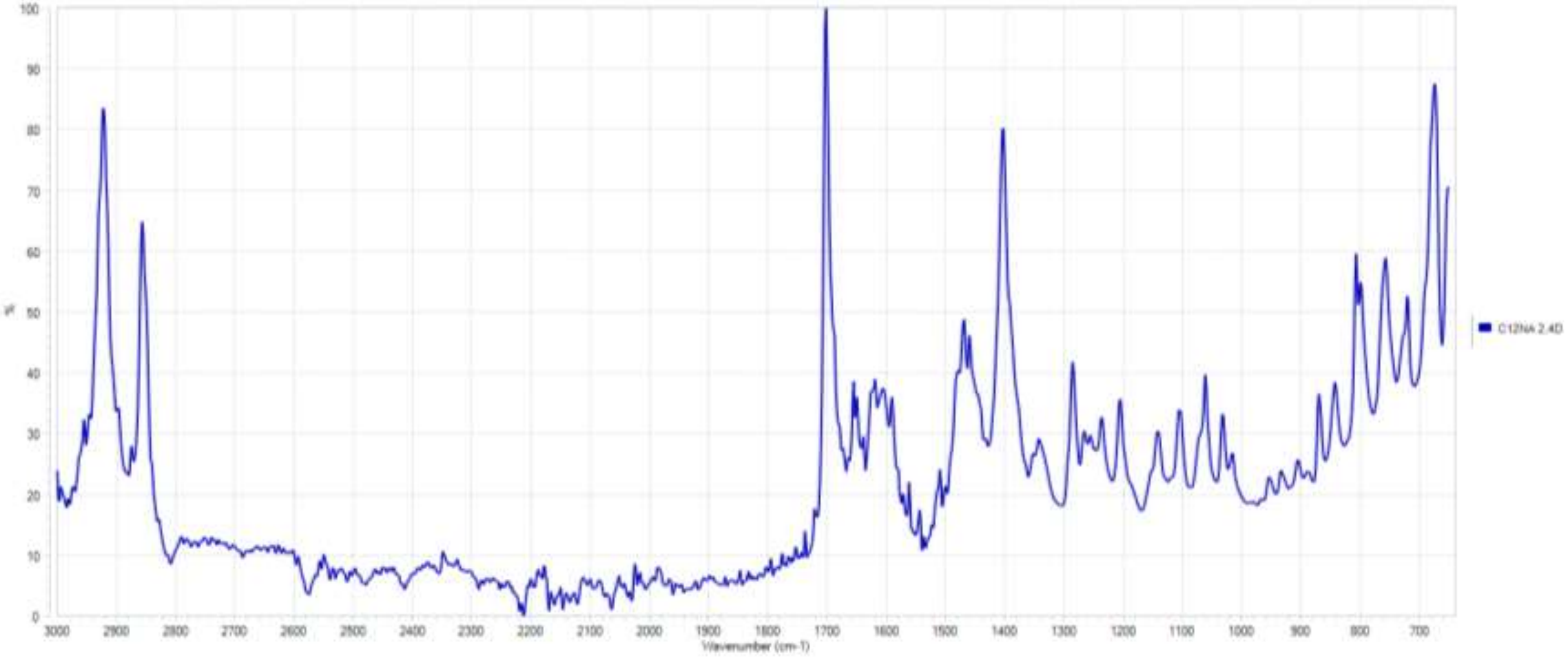

Figure S21. *^{1}H NMR spectrum of N-dodecylonicotinamide 2,4-dichlorophenoxyacetate (**7**).*

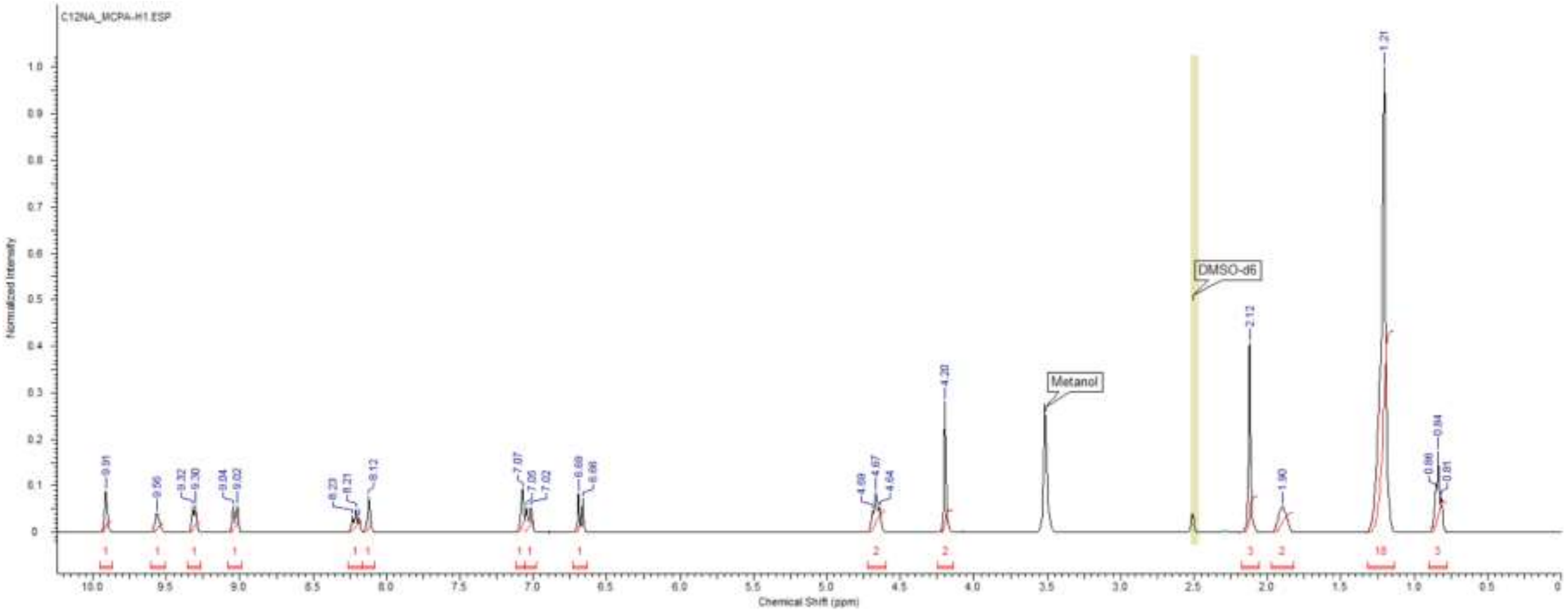

Figure S22. *^{13}C NMR spectrum of N-dodecylonicotinamide 2,4-dichlorophenoxyacetate (**7**).*

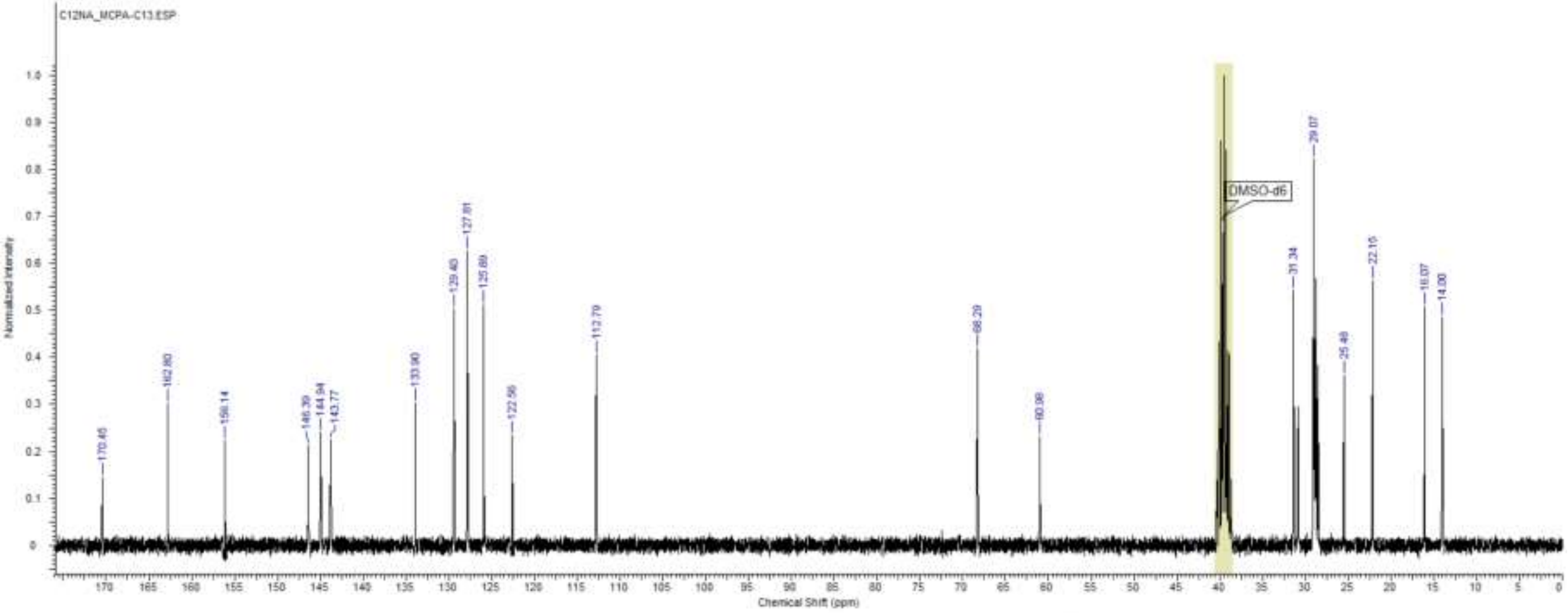

Figure S23. *UV spectrum of N-tetradecylonicotinamide 2,4-dichlorophenoxyacetate (**8**).*

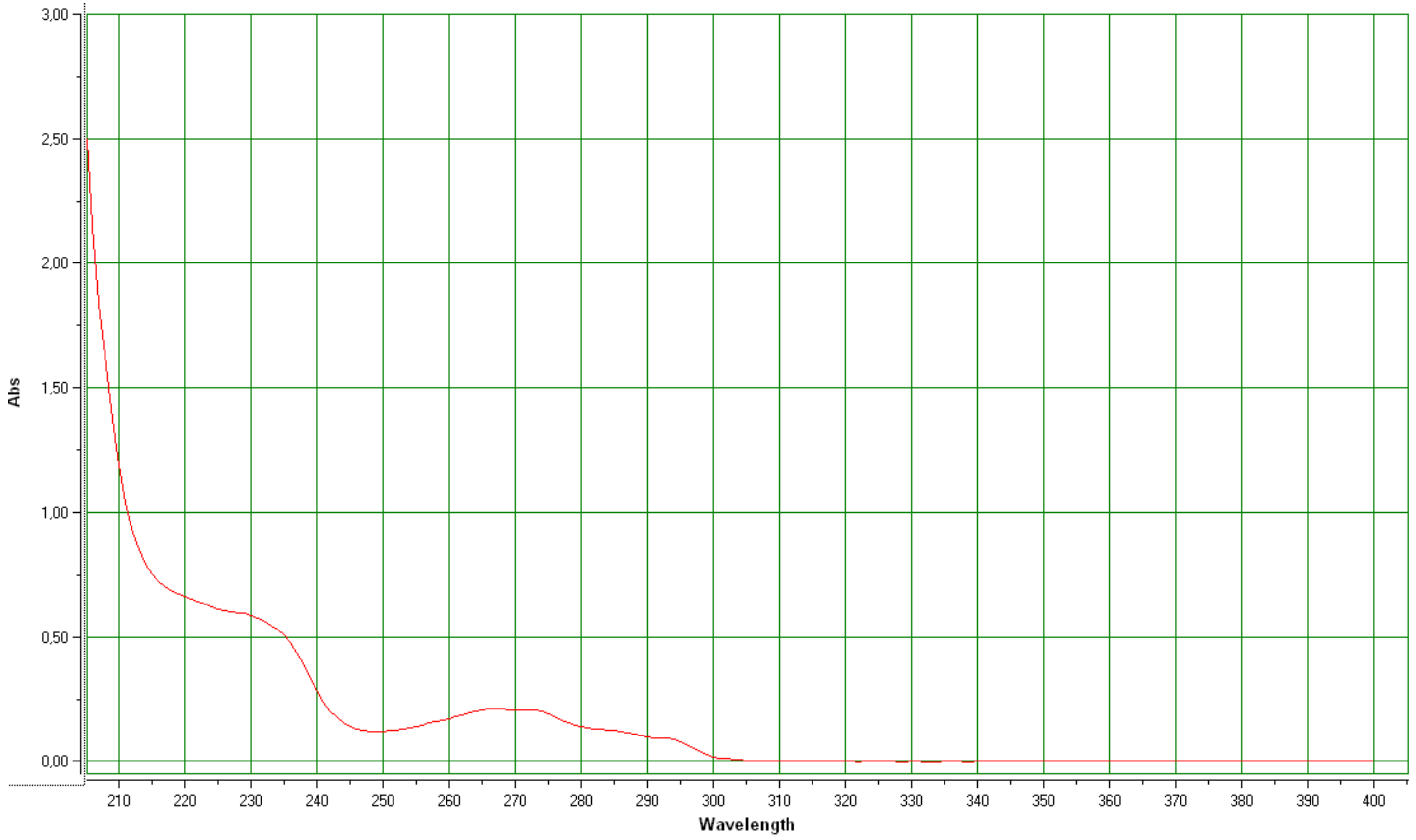

Figure S24. *FT-IR spectrum of N-tetradecylonicotinamide 2,4-dichlorophenoxyacetate (**8**).*

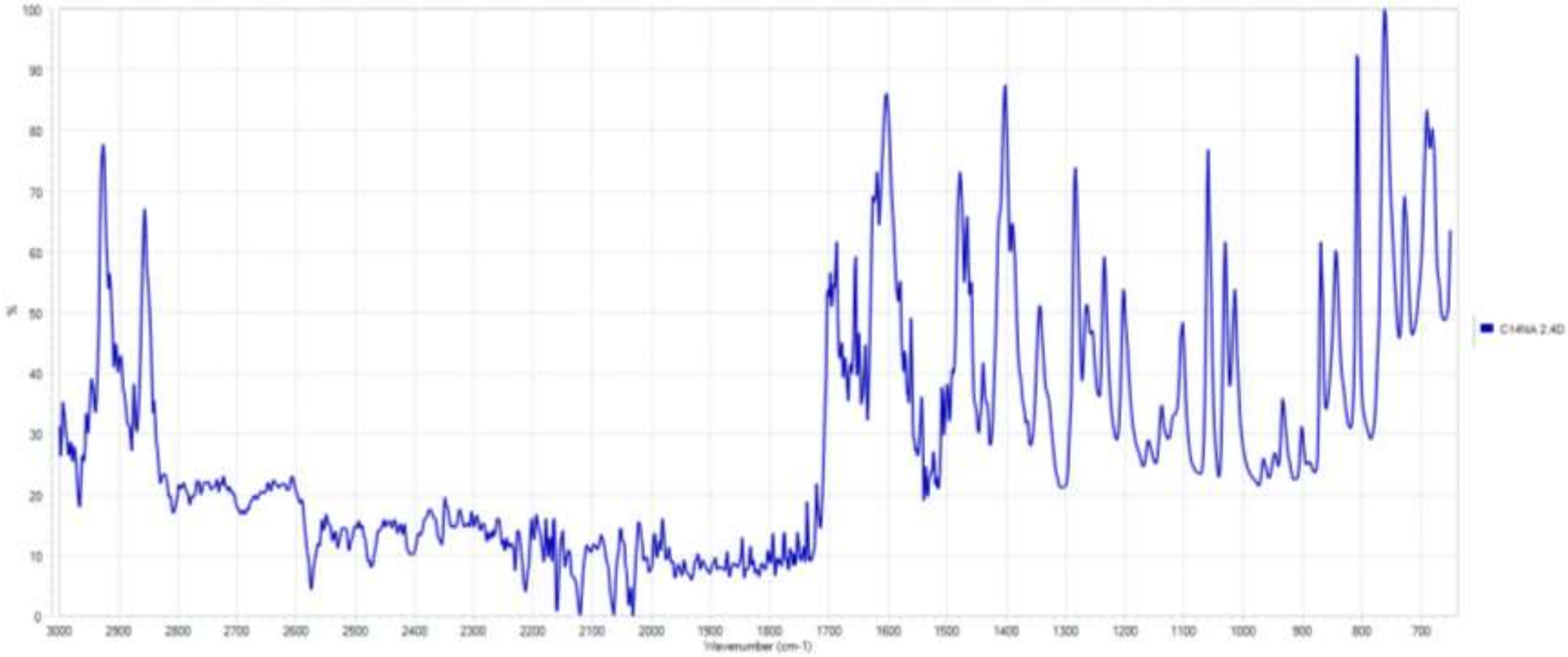

Figure S25. *^{1}H NMR spectrum of N-tetradecylonicotinamide 2,4-dichlorophenoxyacetate (**8**).*

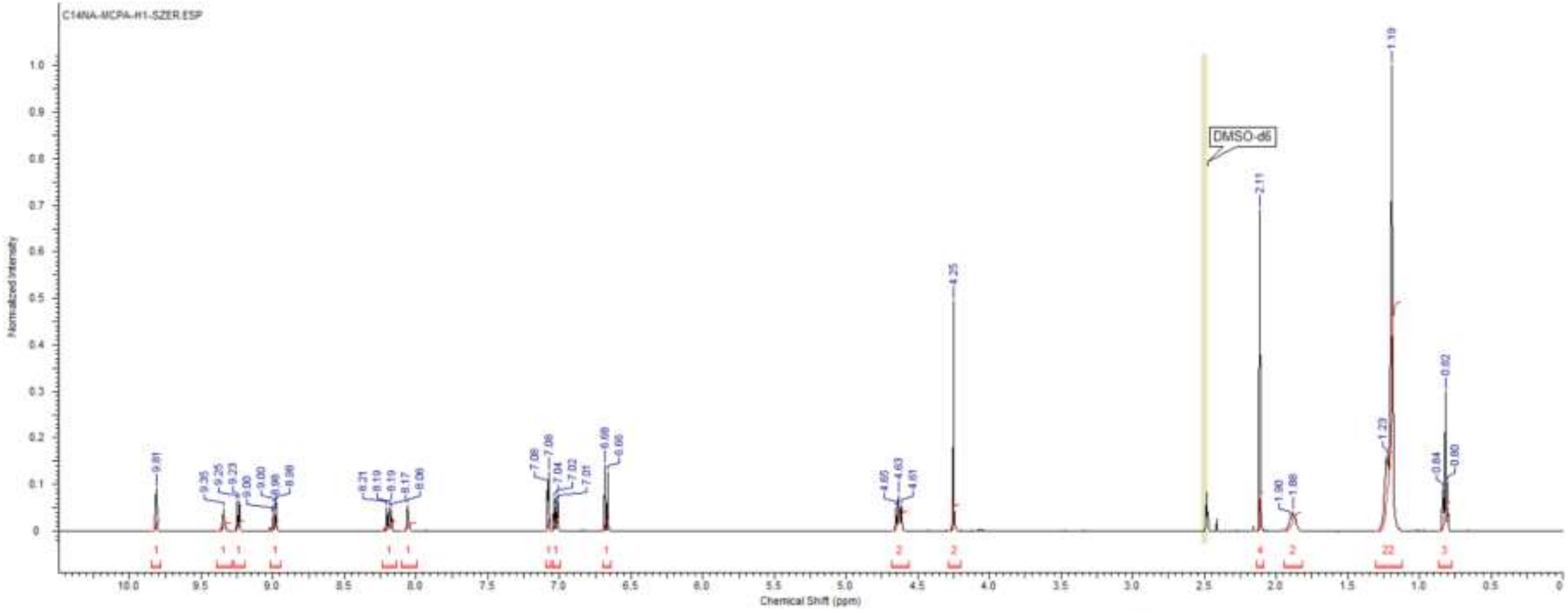

Figure S26. [13]*C NMR spectrum of N-tetradecylonicotinamide 2,4-dichlorophenoxyacetate (**8**).*

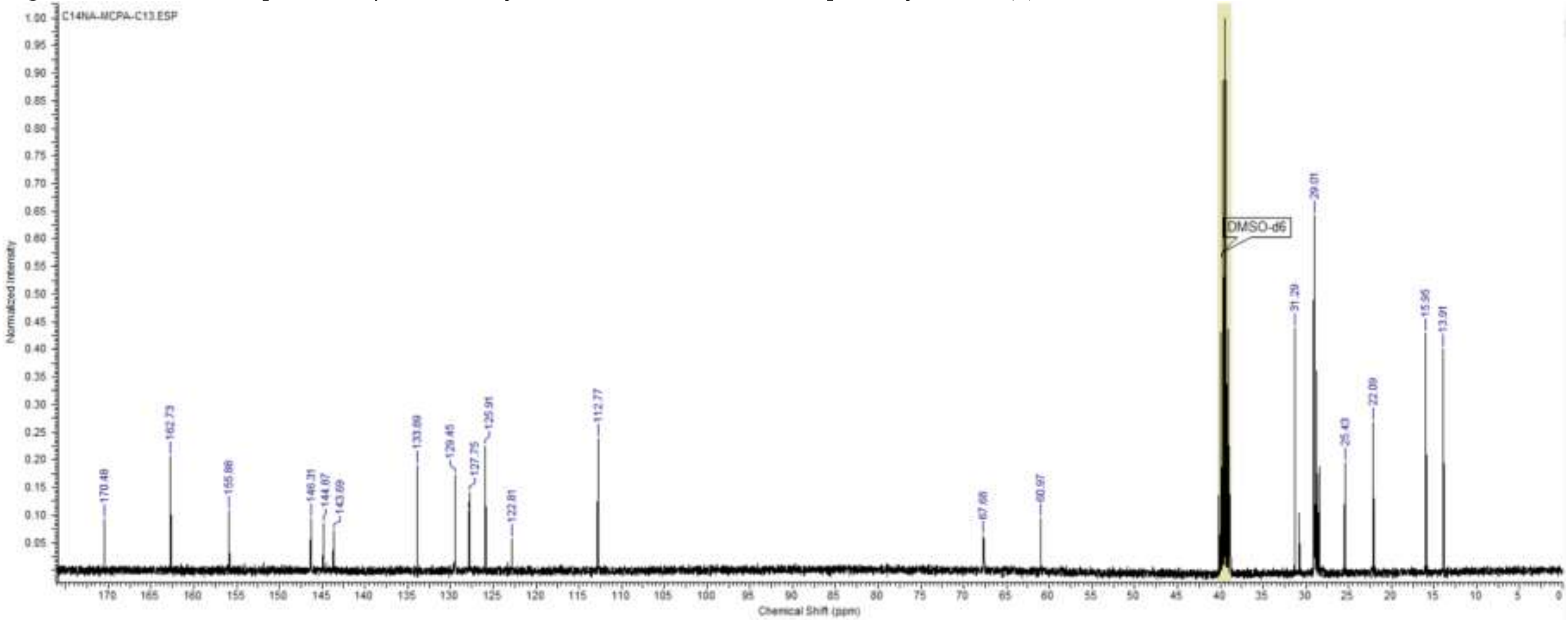

***Figure S27**. UV spectrum of N-hexadecylonicotinamide 2,4-dichlorophenoxyacetate (**9**).*

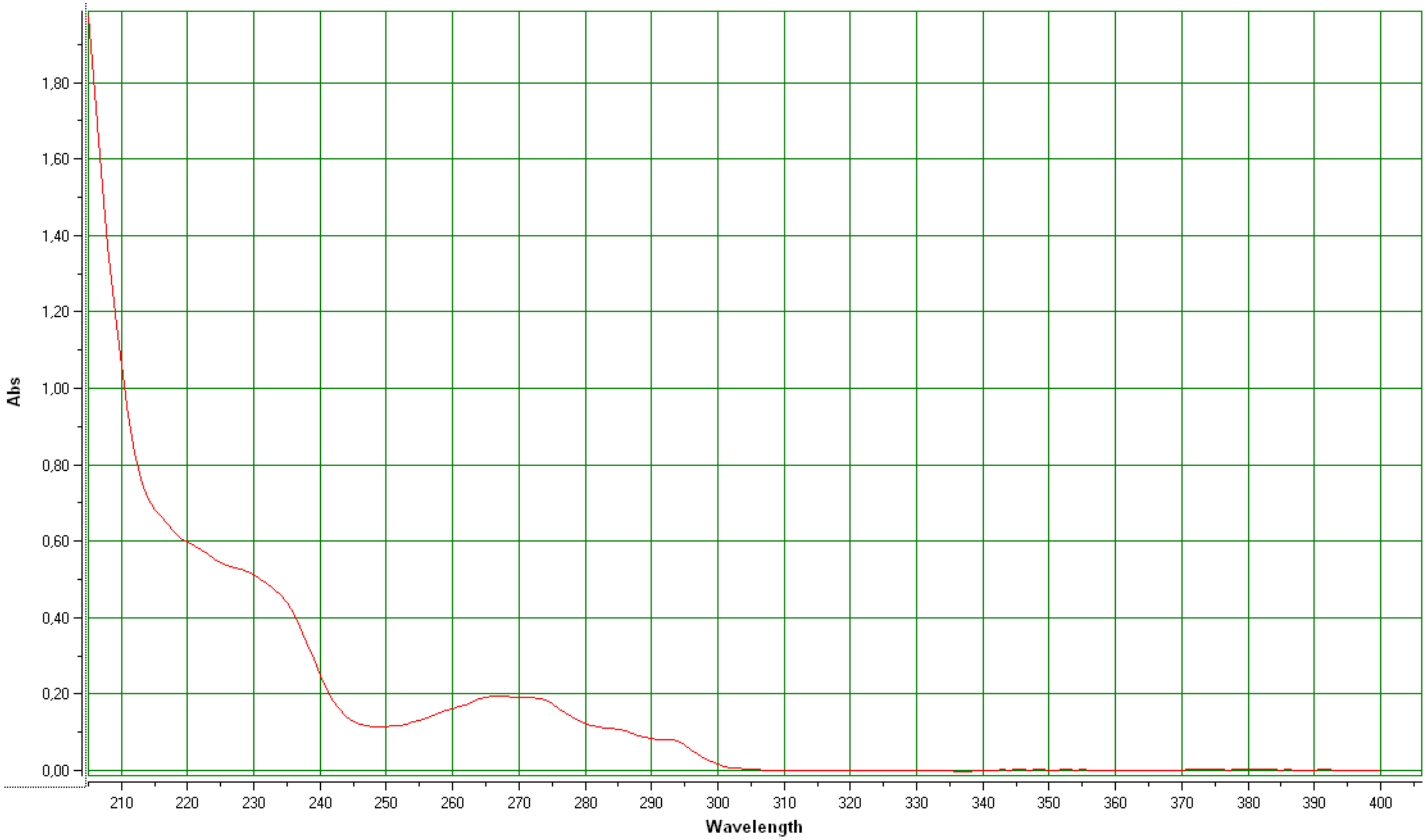

Figure S28. *FT-IR spectrum of N-hexadecylonicotinamide 2,4-dichlorophenoxyacetate (**9**).*

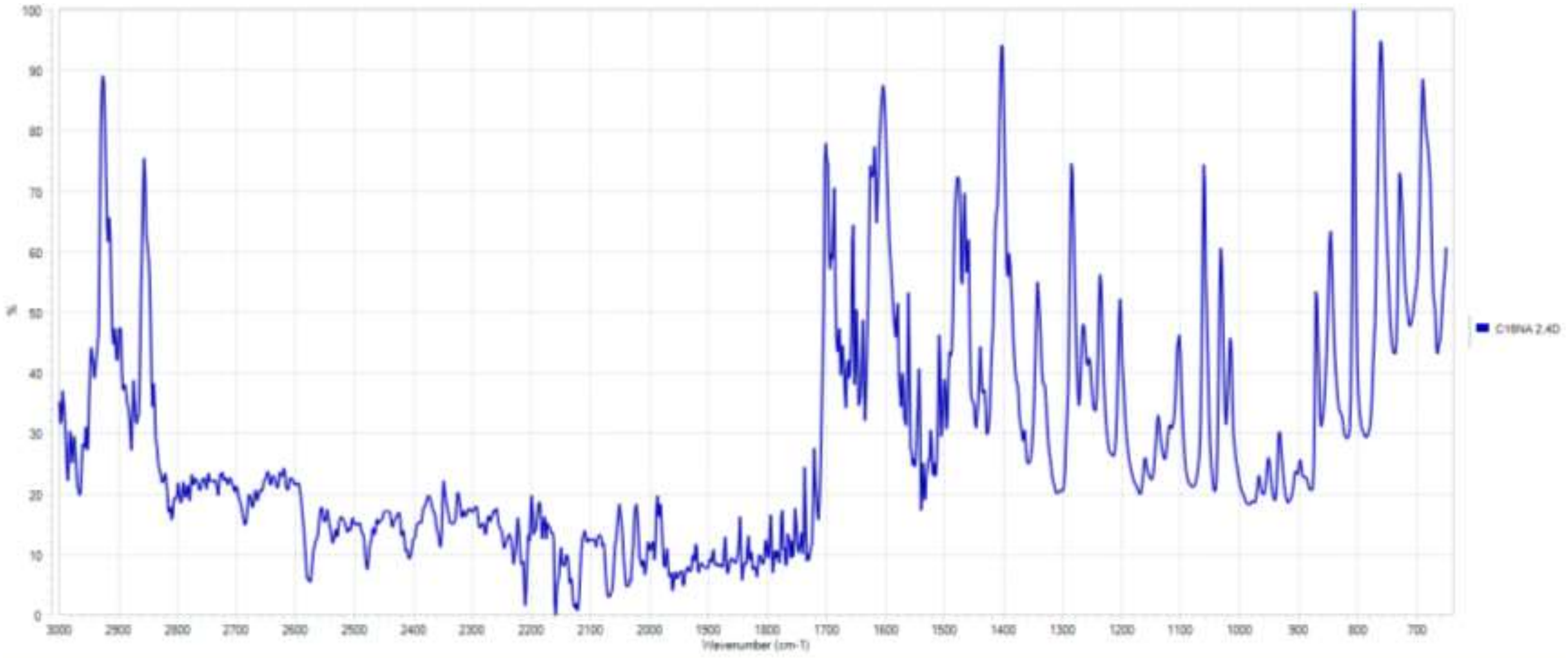

Figure S29. *^{1}H NMR spectrum of N-hexadecylonicotinamide 2,4-dichlorophenoxyacetate (**9**).*

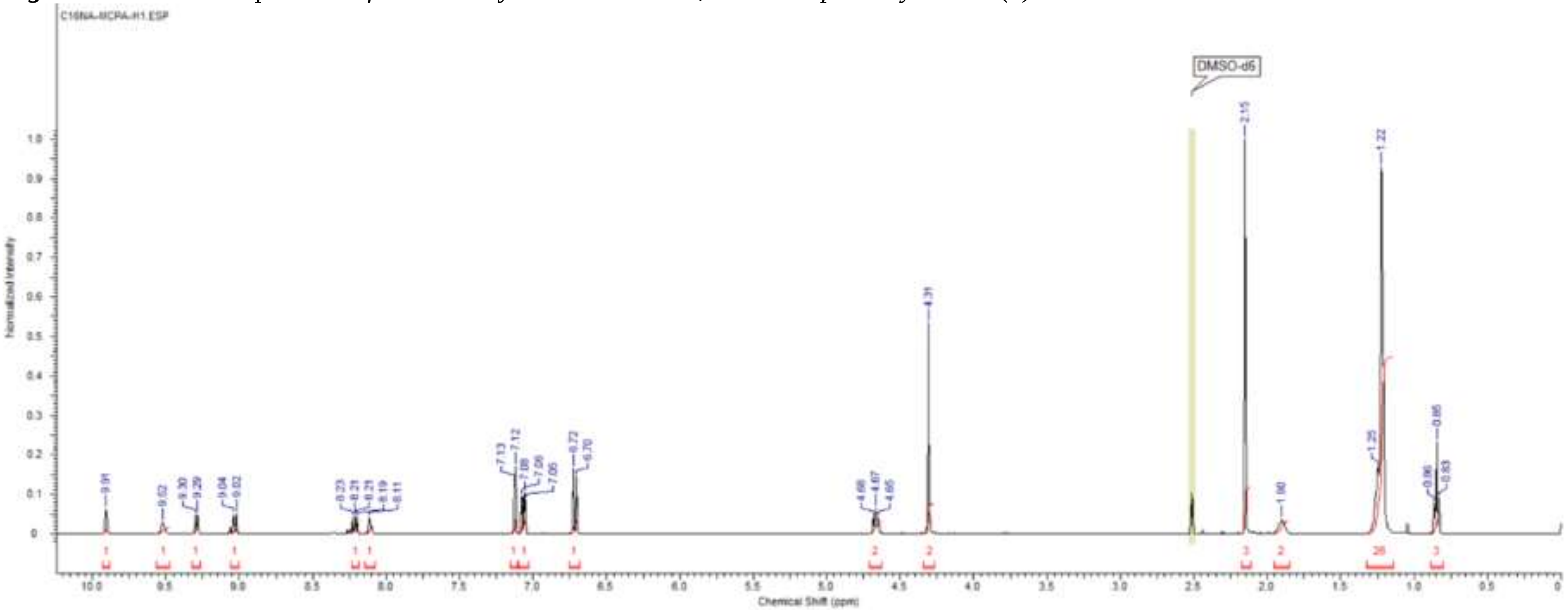

Figure S30. *^{13}C NMR spectrum of N-hexadecylonicotinamide 2,4-dichlorophenoxyacetate (**9**).*

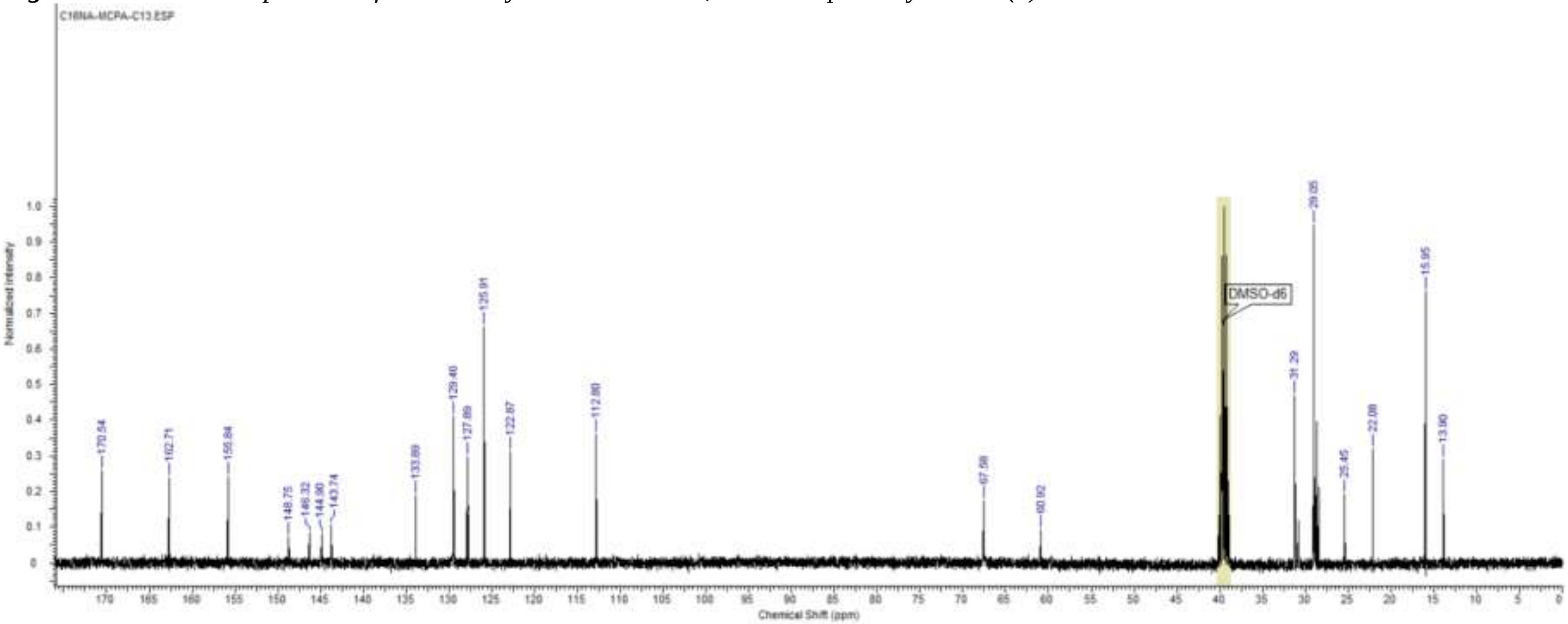

Figure S31. *UV spectrum of N-octadecylonicotinamide 2,4-dichlorophenoxyacetate* (**10**).

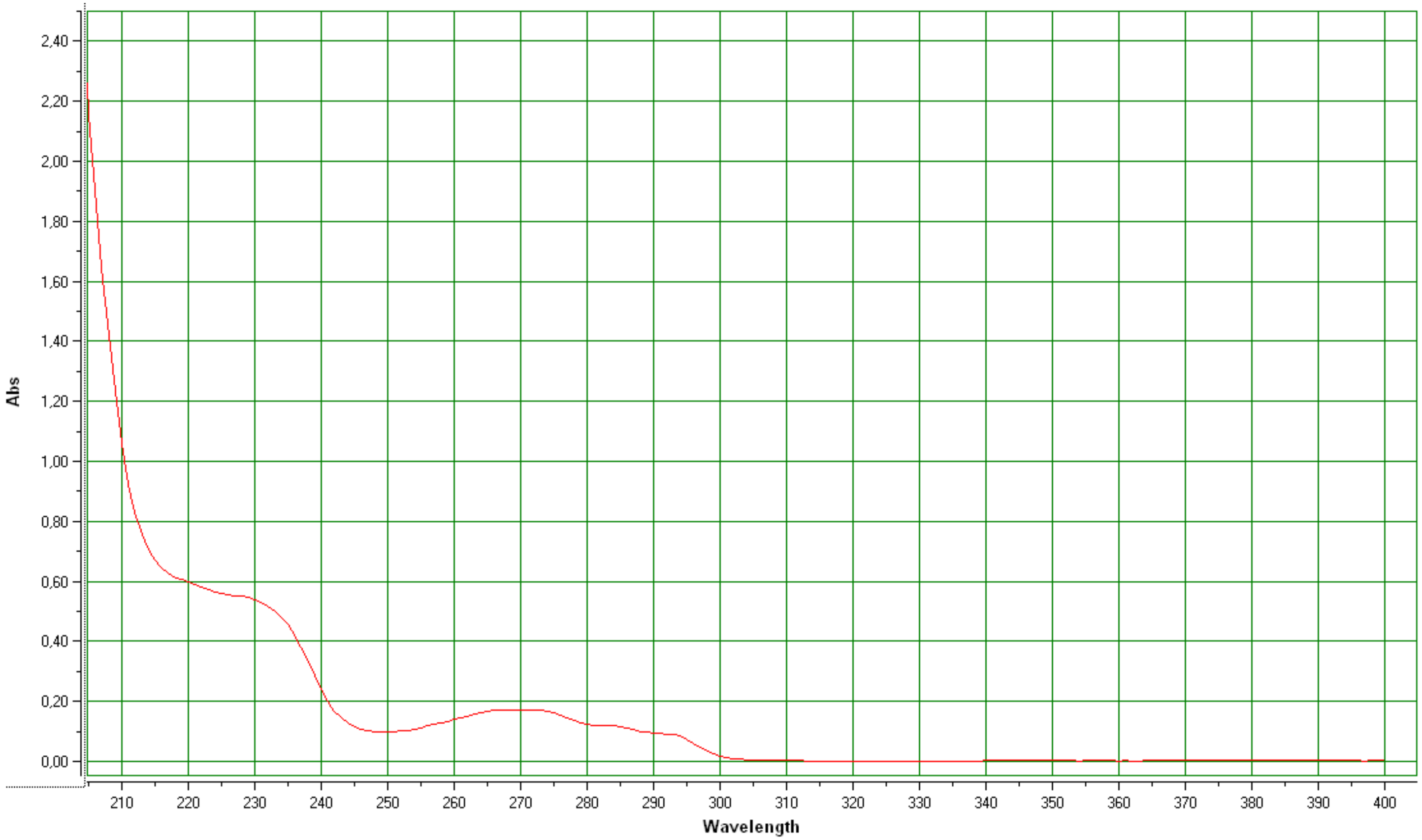

Figure S32. *FT-IR spectrum of N-octadecylonicotinamide 2,4-dichlorophenoxyacetate* (***10***).

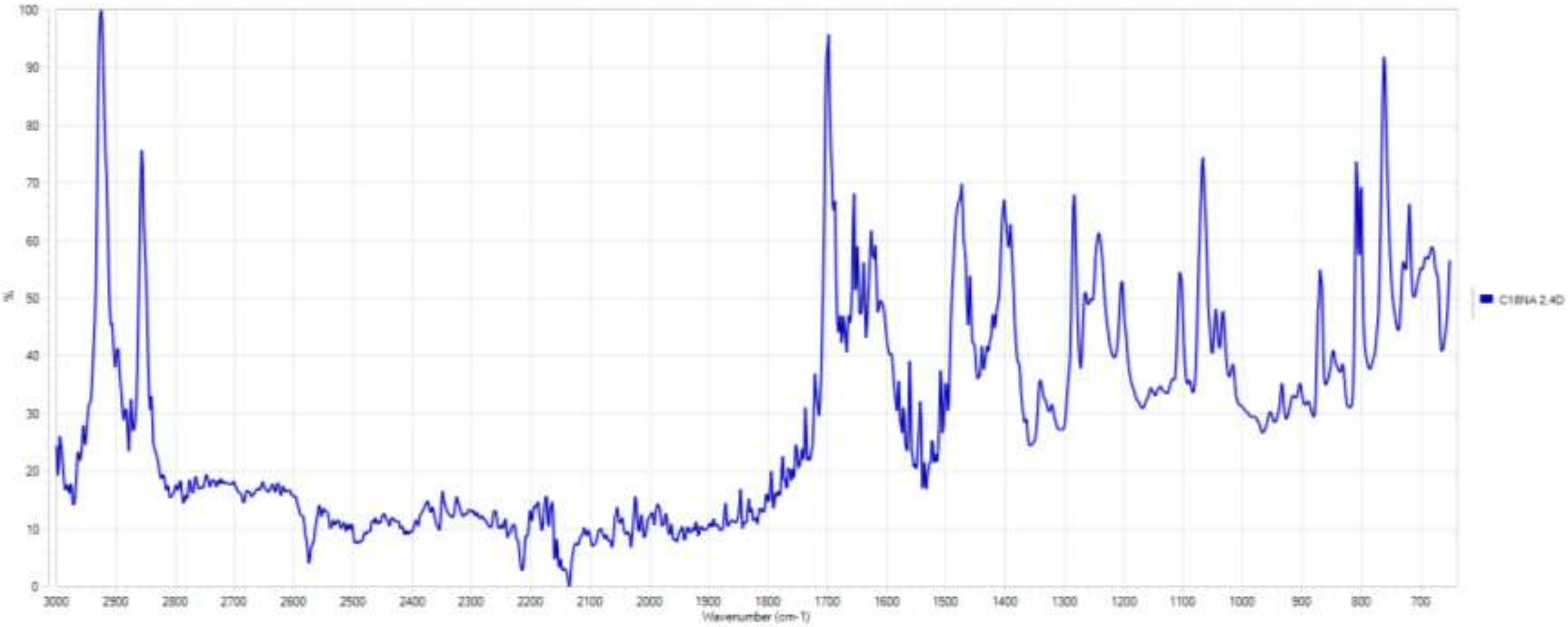

Figure S33. *^{1}H NMR spectrum of N-octadecylonicotinamide 2,4-dichlorophenoxyacetate (**10**).*

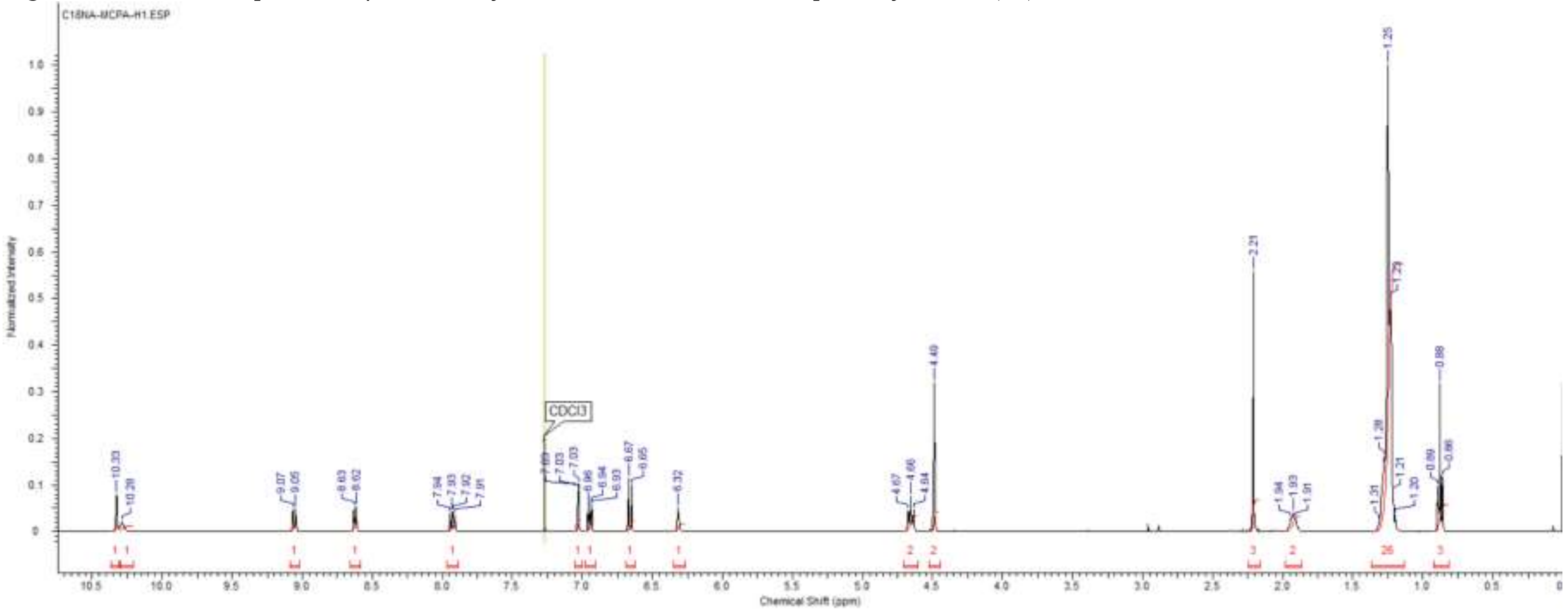

Figure S34. *^{13}C NMR spectrum of N-octadecylonicotinamide 2,4-dichlorophenoxyacetate (**10**).*

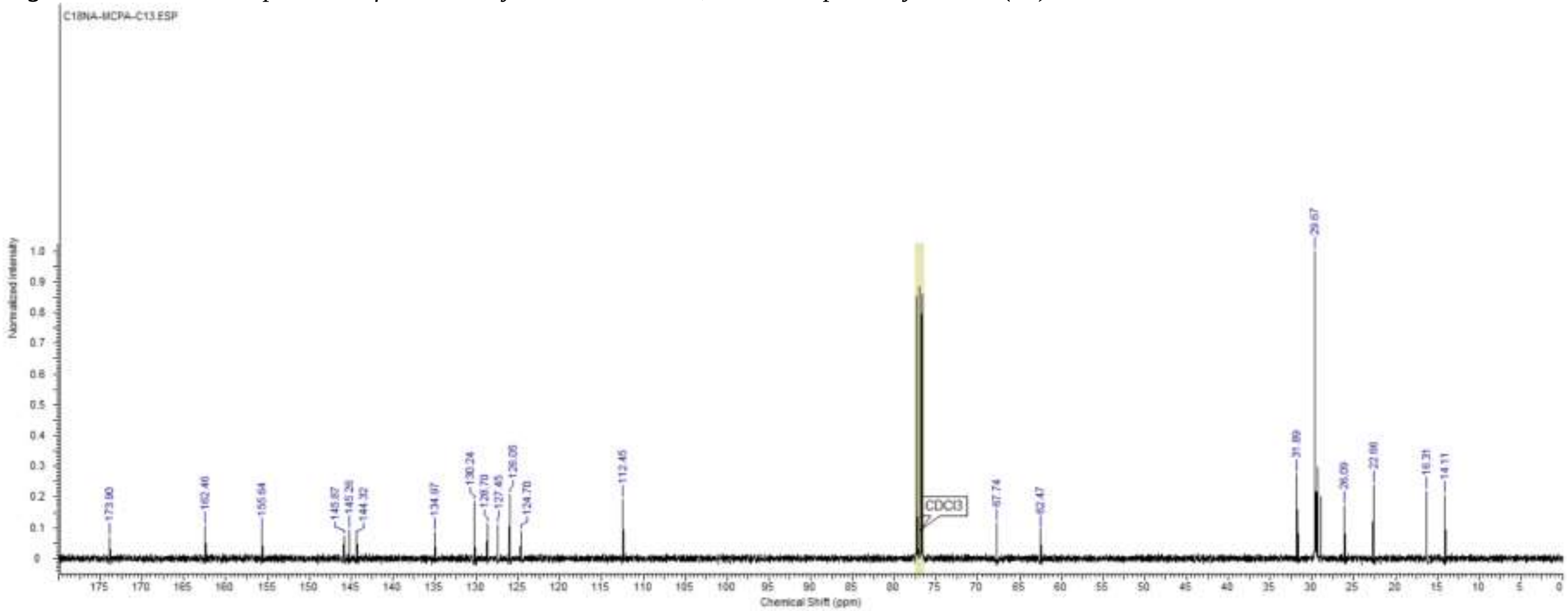

Figure S35. *UV spectrum of N-decylonicotinamide 4-chloro-2-methylphenoxyacetate (**11**).*

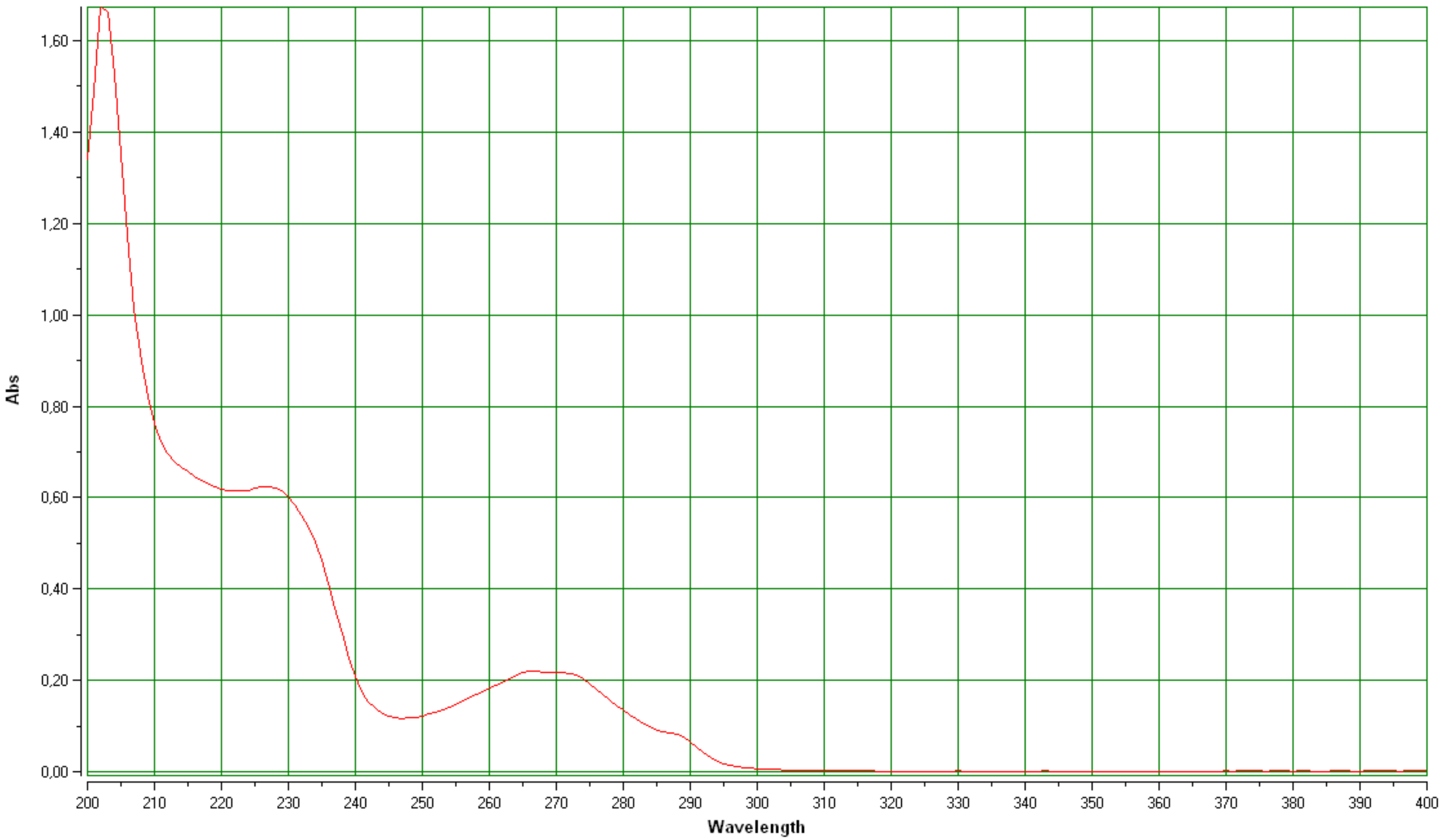

***Figure S36**. FT-IR spectrum of N-decylonicotinamide 4-chloro-2-methylphenoxyacetate (**11**).*

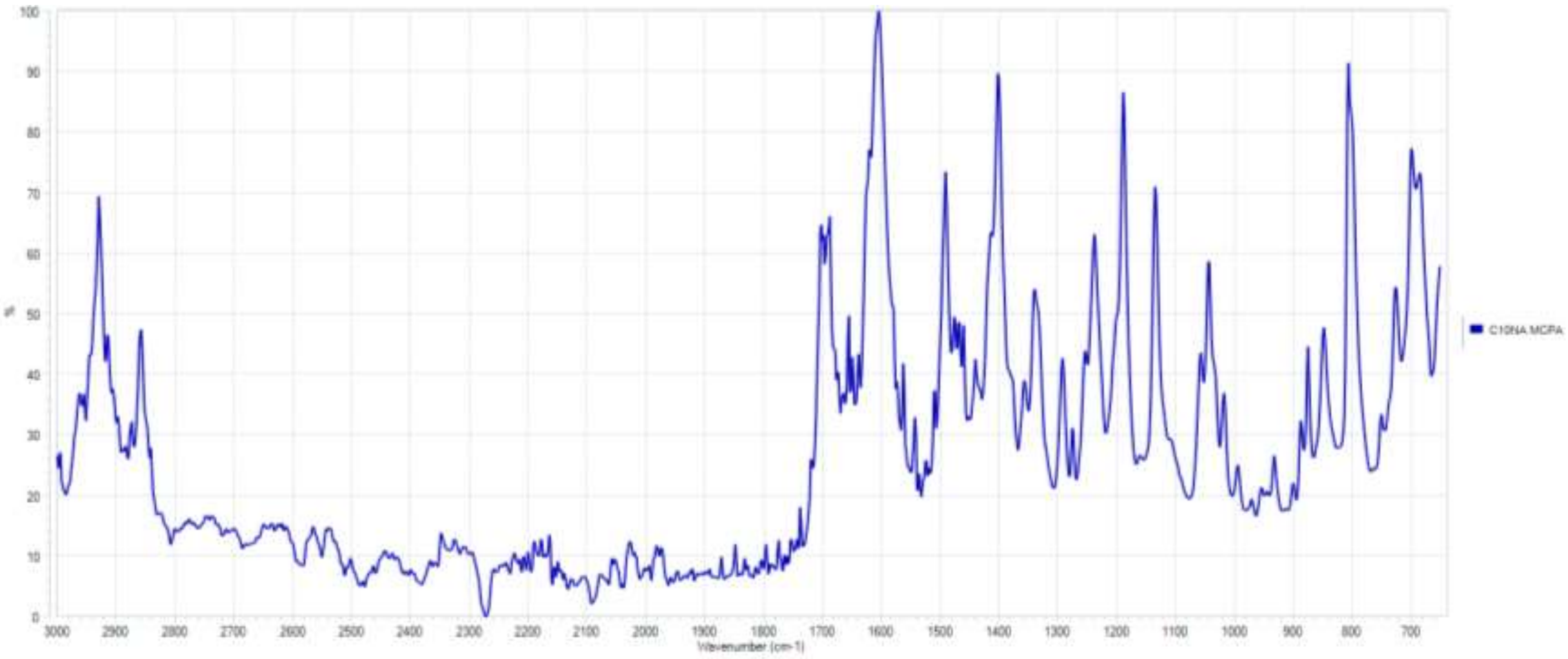

Figure S37. *^{1}H NMR spectrum of N-decylonicotinamide 4-chloro-2-methylphenoxyacetate (**11**).*

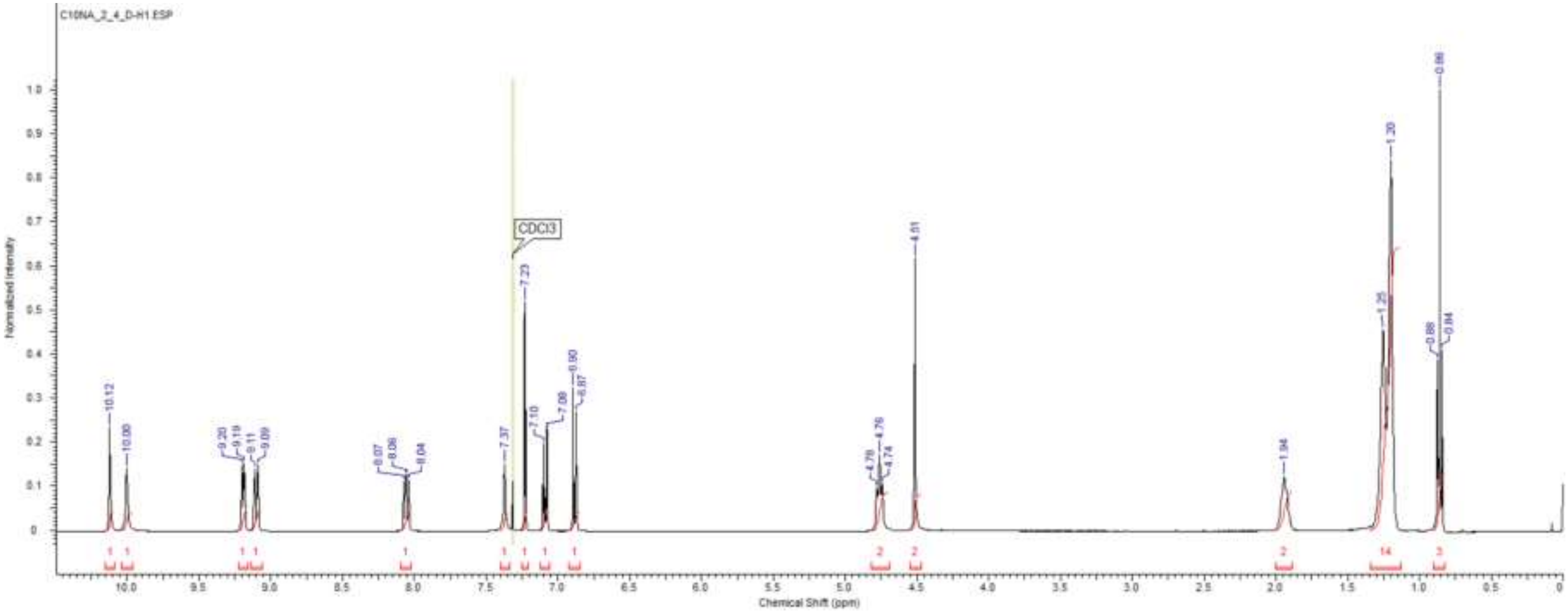

Figure S38. *^{13}C NMR spectrum of N-decylonicotinamide 4-chloro-2-methylphenoxyacetate (**11**).*

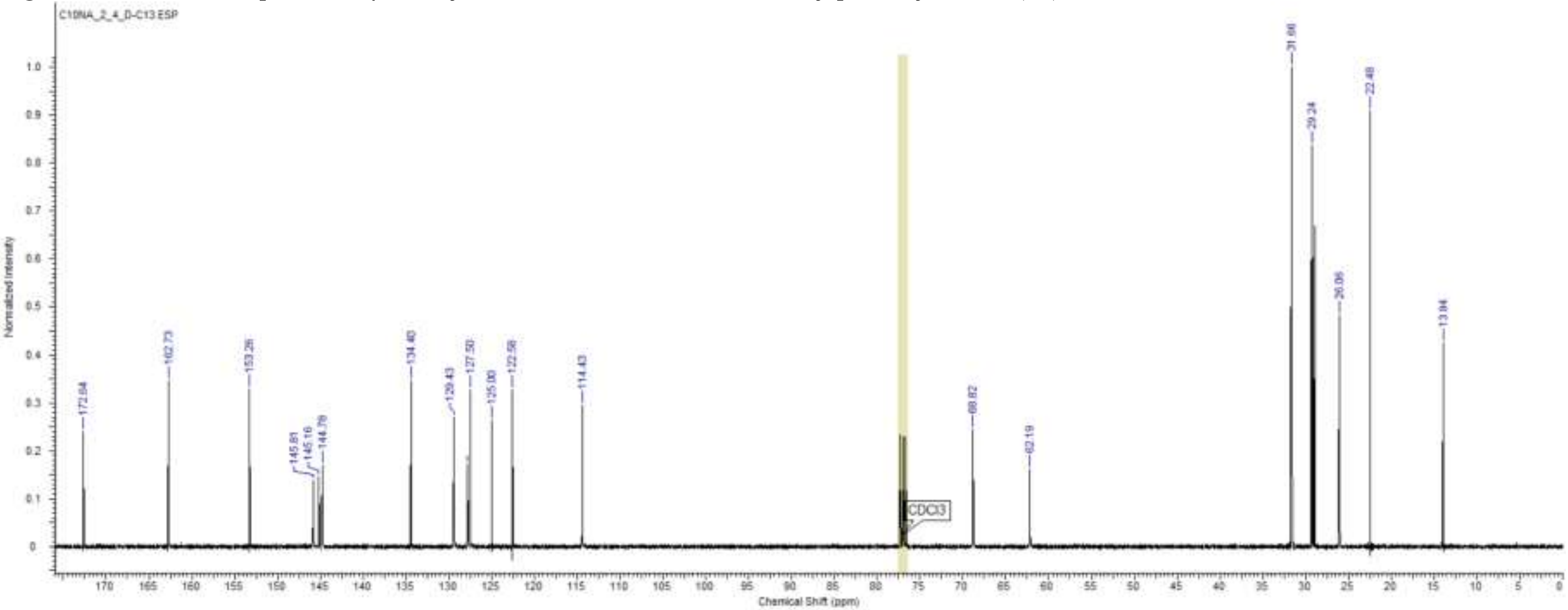

Figure S39. *UV spectrum of N-dodecylonicotinamide 4-chloro-2-methylphenoxyacetate (**12**).*

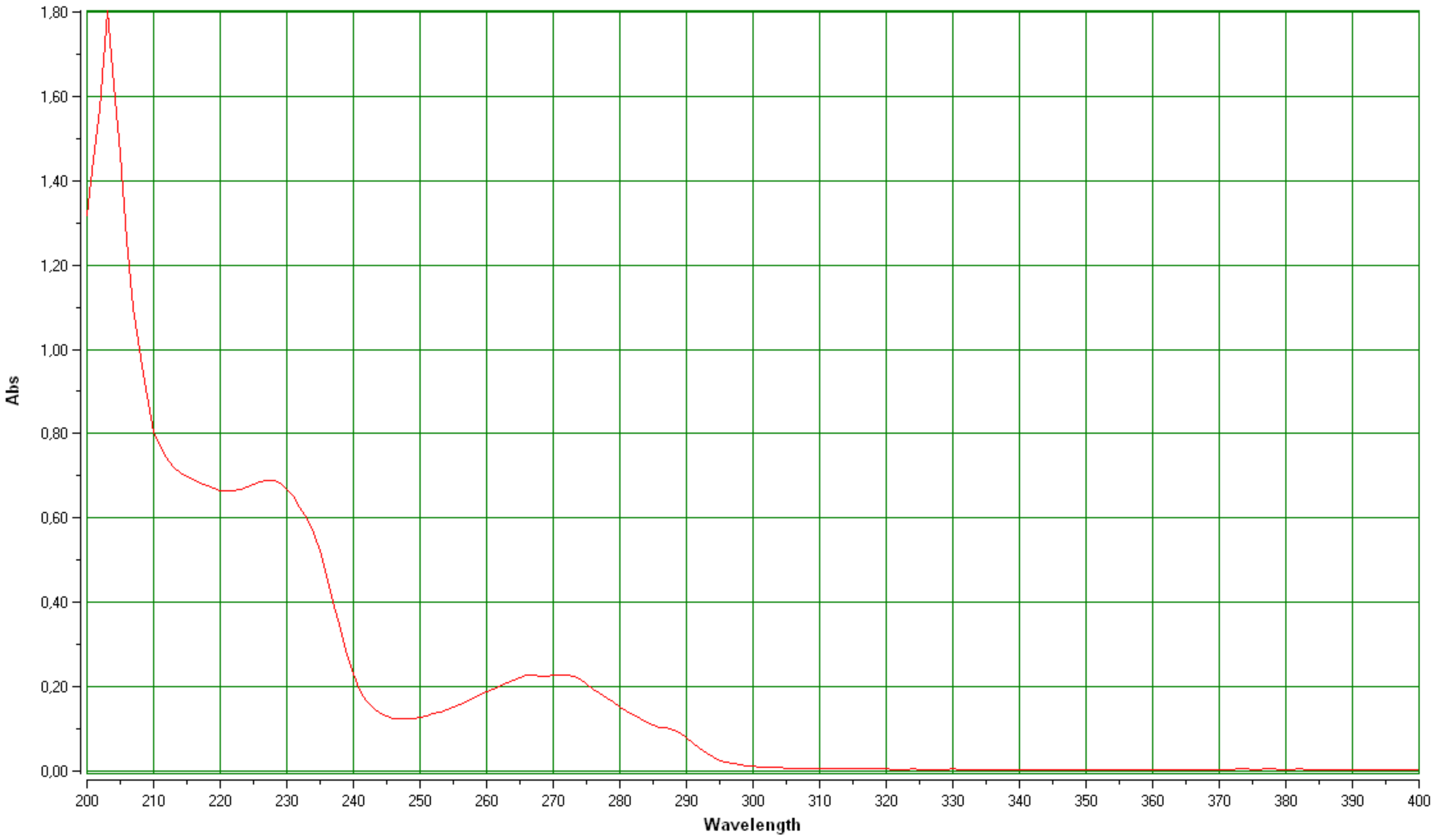

***Figure S40**. FT-IR spectrum of N-dodecylonicotinamide 4-chloro-2-methylphenoxyacetate (**12**).*

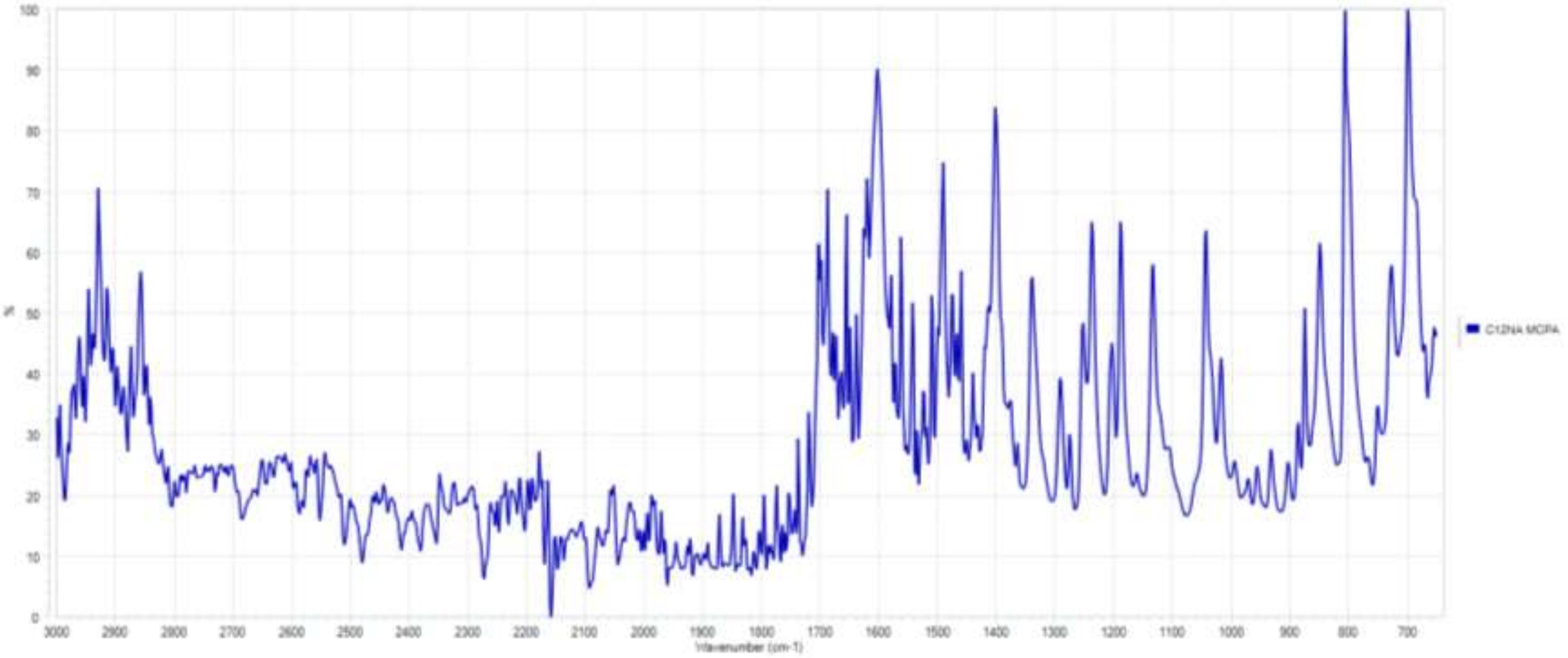

Figure S41. *^{1}H NMR spectrum of N-dodecylonicotinamide 4-chloro-2-methylphenoxyacetate* ***(12)***.

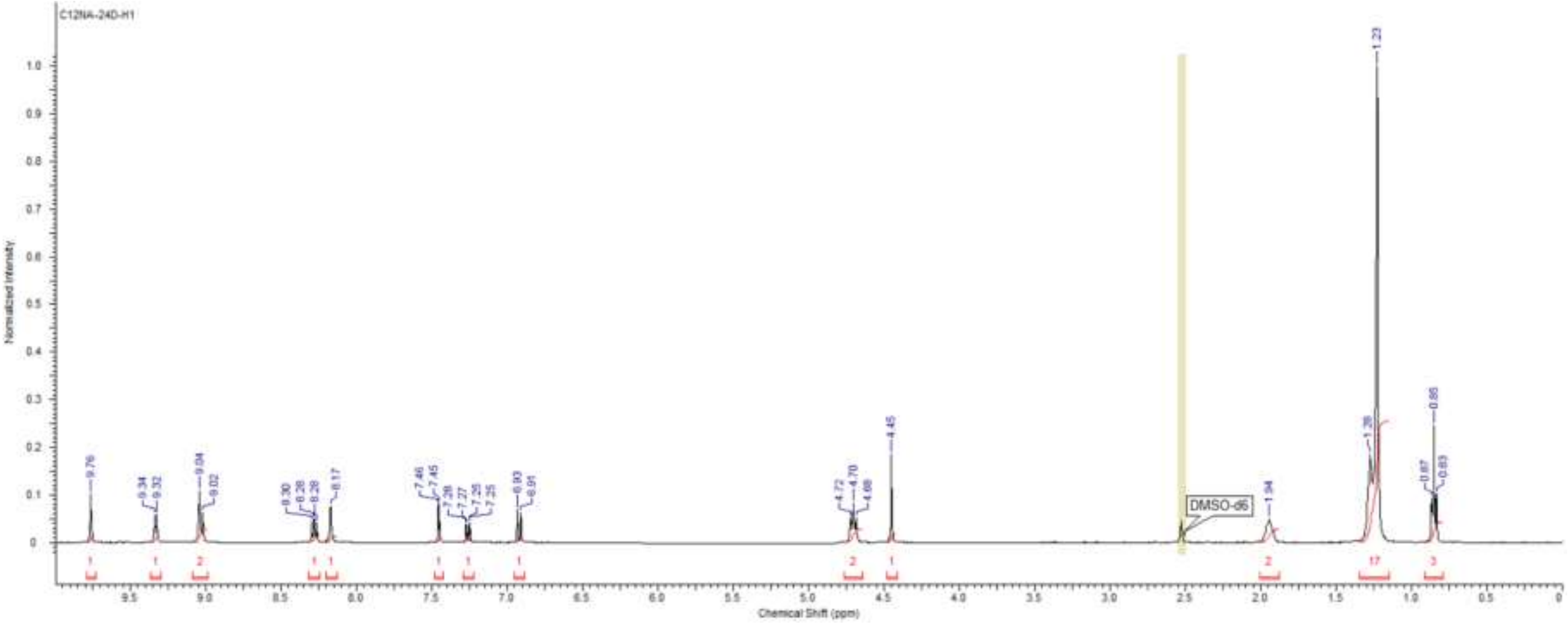

Figure S42. *^{13}C NMR spectrum of N-dodecylonicotinamide 4-chloro-2-methylphenoxyacetate (**12**).*

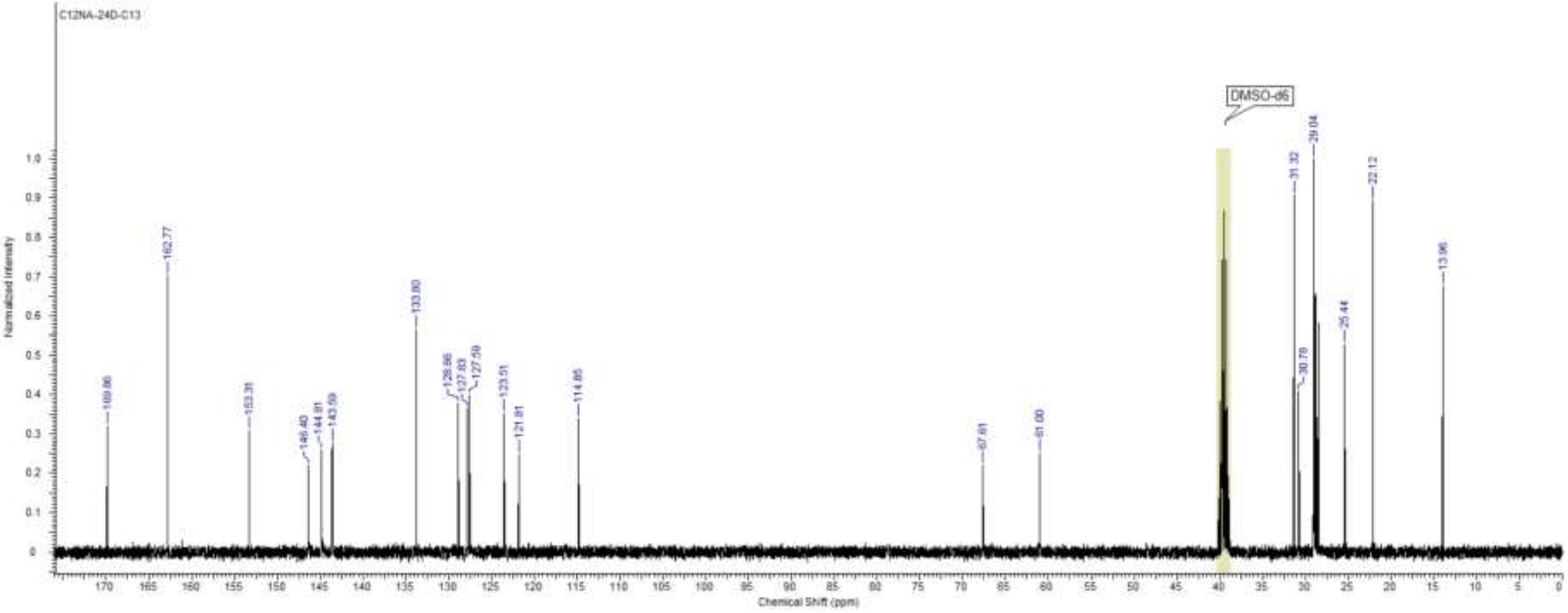

Figure S43. *UV spectrum of N-tetradecylonicotinamide 4-chloro-2-methylphenoxyacetate (**13**).*

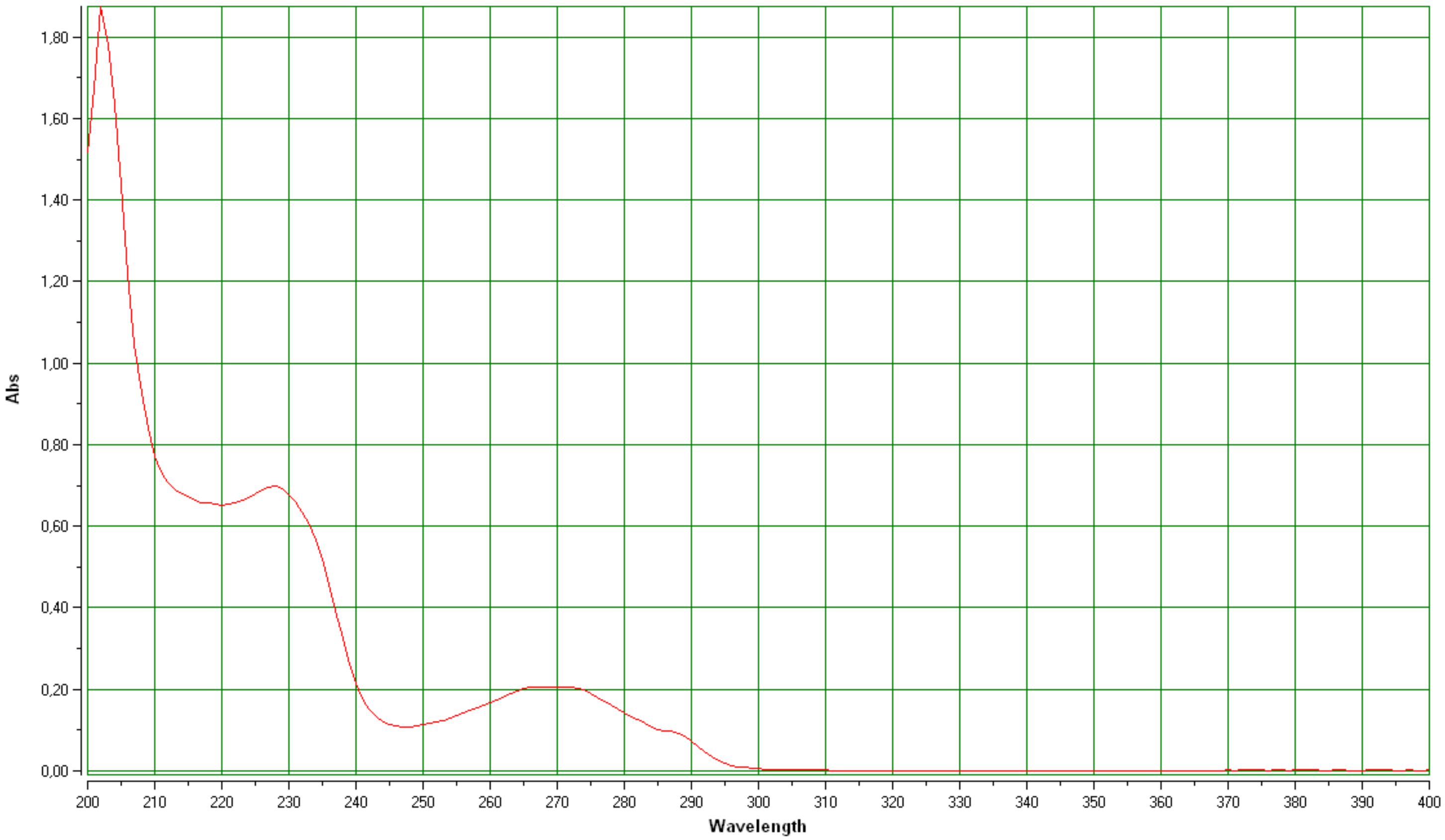

Figure S44*. FT-IR spectrum of N-tetradecylonicotinamide 4-chloro-2-methylphenoxyacetate (****13****).*

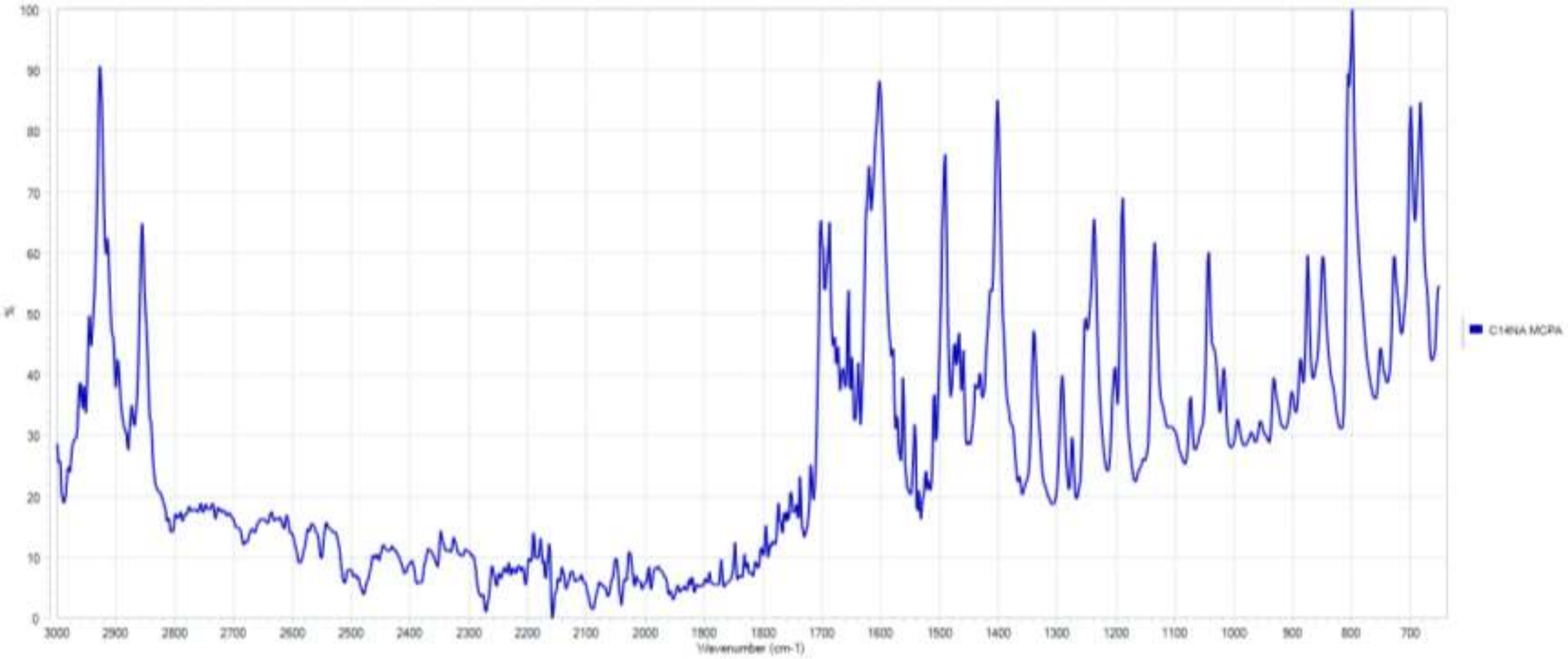

Figure S45. *^{1}H NMR spectrum of N-tetradecylonicotinamide 4-chloro-2-methylphenoxyacetate* (***13***).

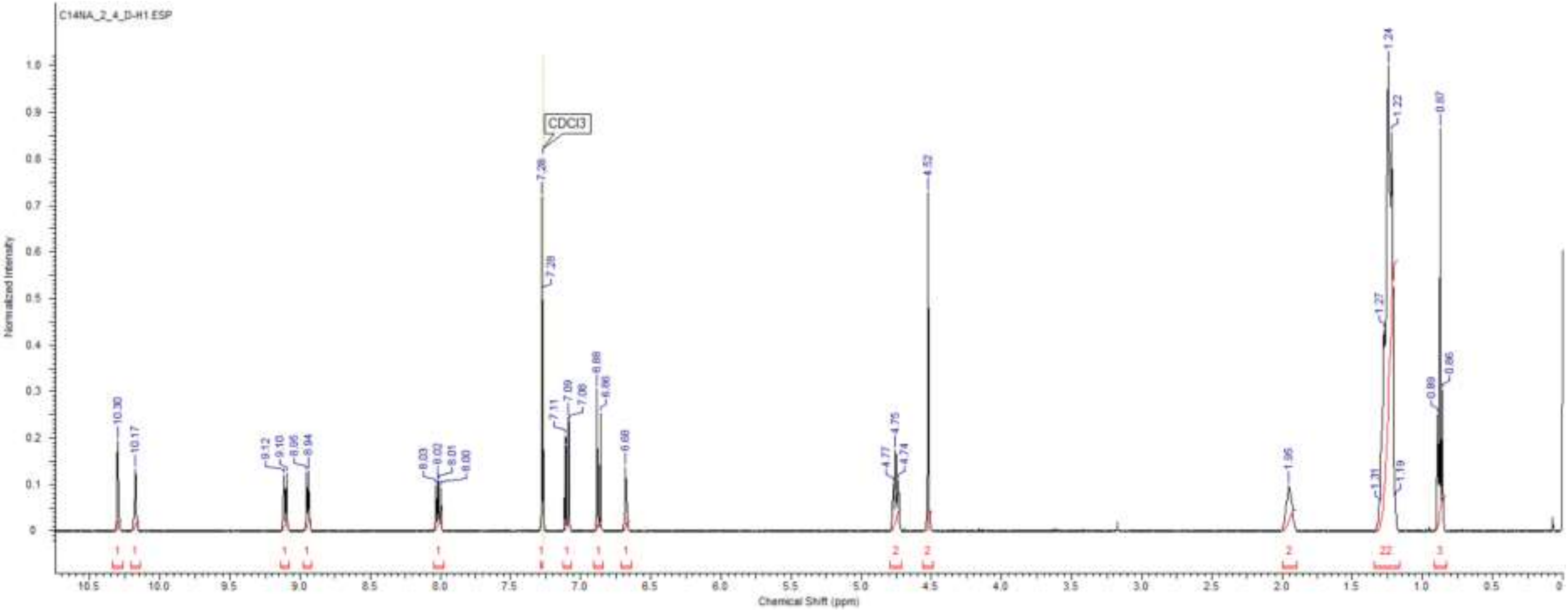

Figure S46. *^{13}C NMR spectrum of N-tetradecylonicotinamide 4-chloro-2-methylphenoxyacetate (**13**).*

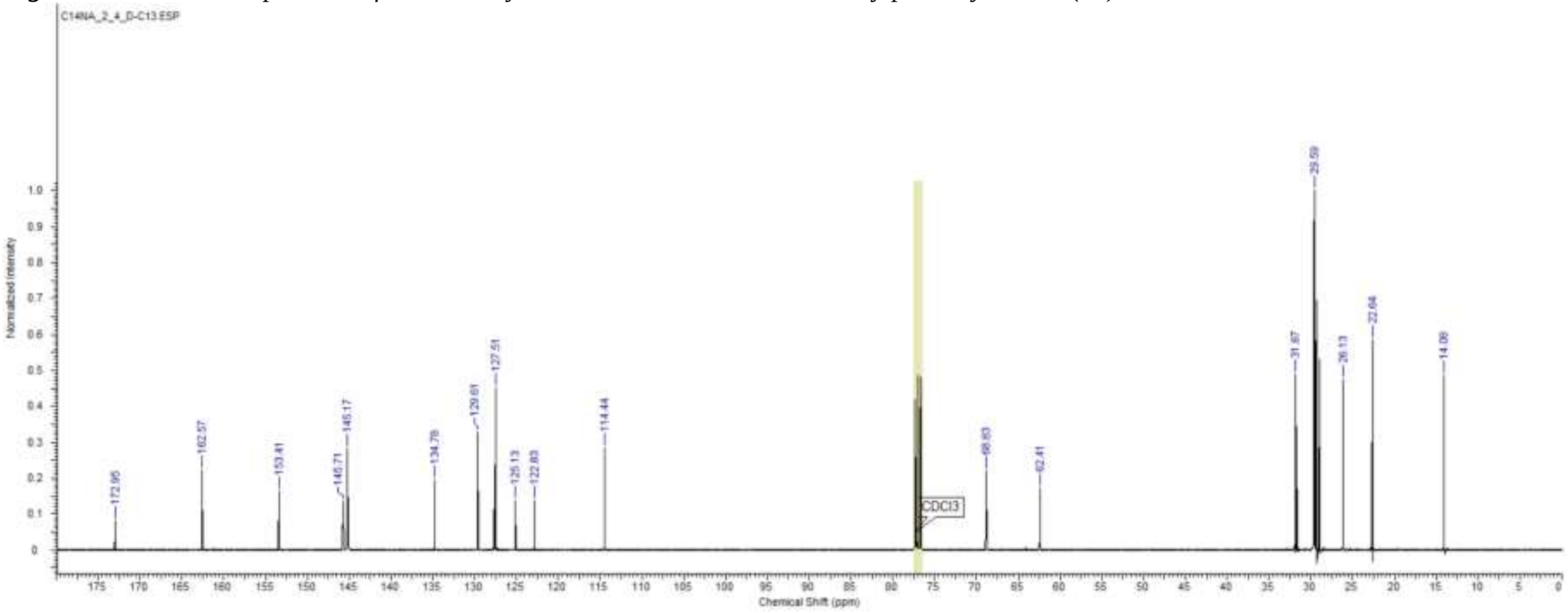

Figure S47. *UV spectrum of N-hexadecylonicotinamide 4-chloro-2-methylphenoxyacetate (**14**).*

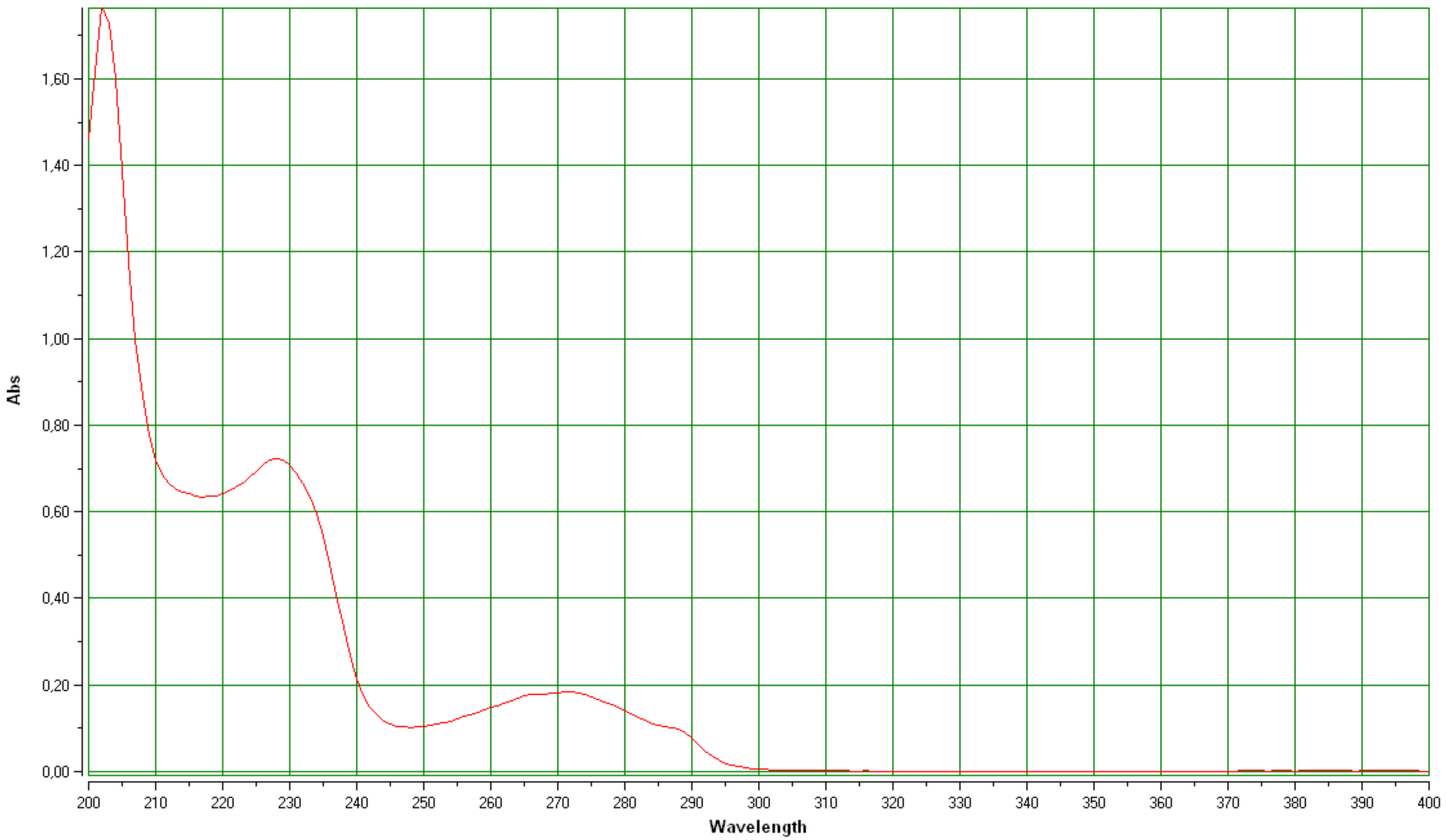

Figure S48. *FT-IR spectrum of N-hexadecylonicotinamide 4-chloro-2-methylphenoxyacetate (**14**).*

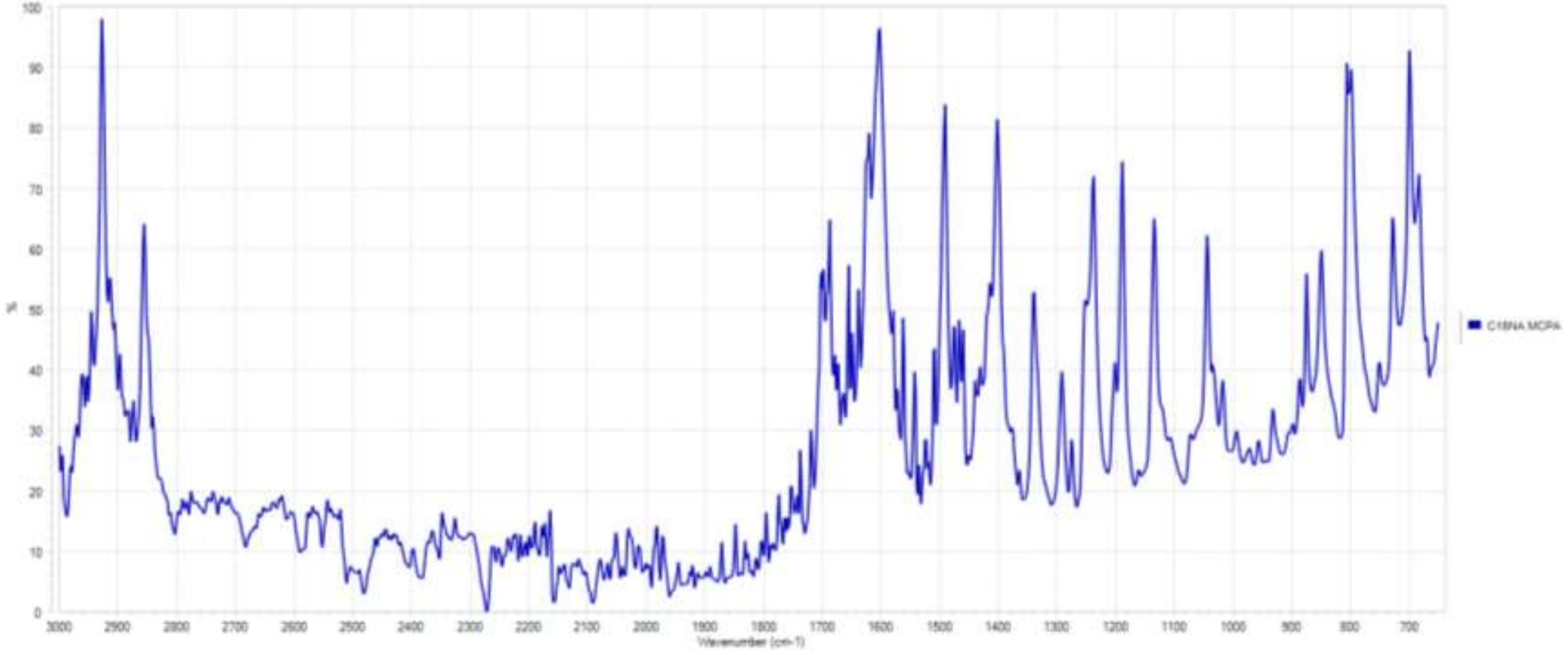

Figure S49. *^{1}H NMR spectrum of N-hexadecylonicotinamide 4-chloro-2-methylphenoxyacetate (**14**).*

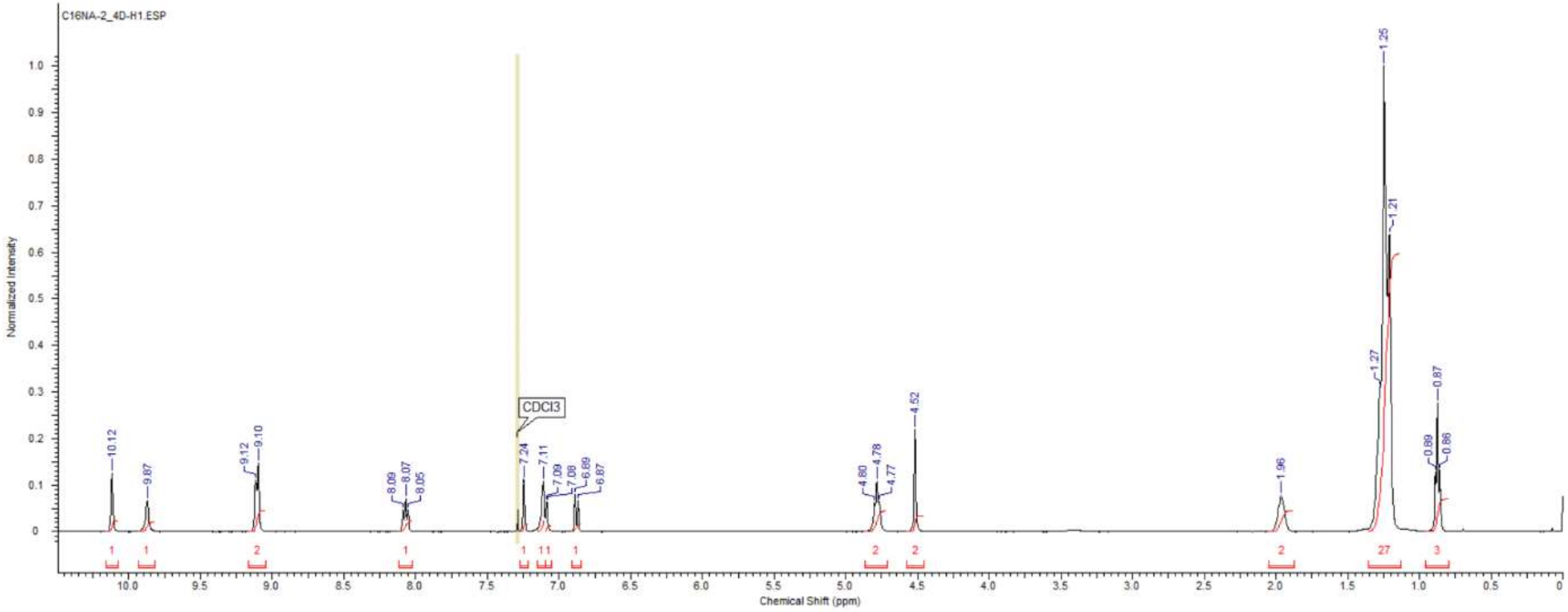

Figure S50. *^{13}C NMR spectrum of N-hexadecylonicotinamide 4-chloro-2-methylphenoxyacetate (**14**).*

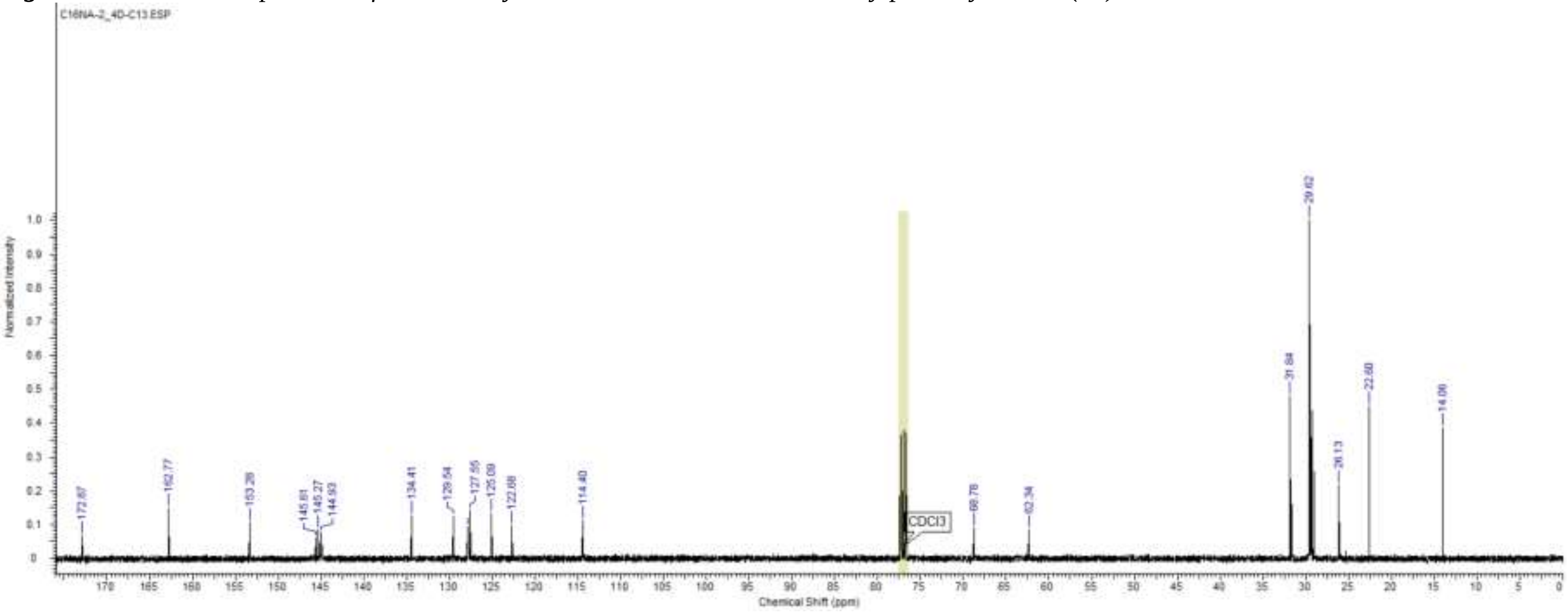

Figure S51. *UV spectrum of N-hexadecylonicotinamide 4-chloro-2-methylphenoxyacetate (**15**).*

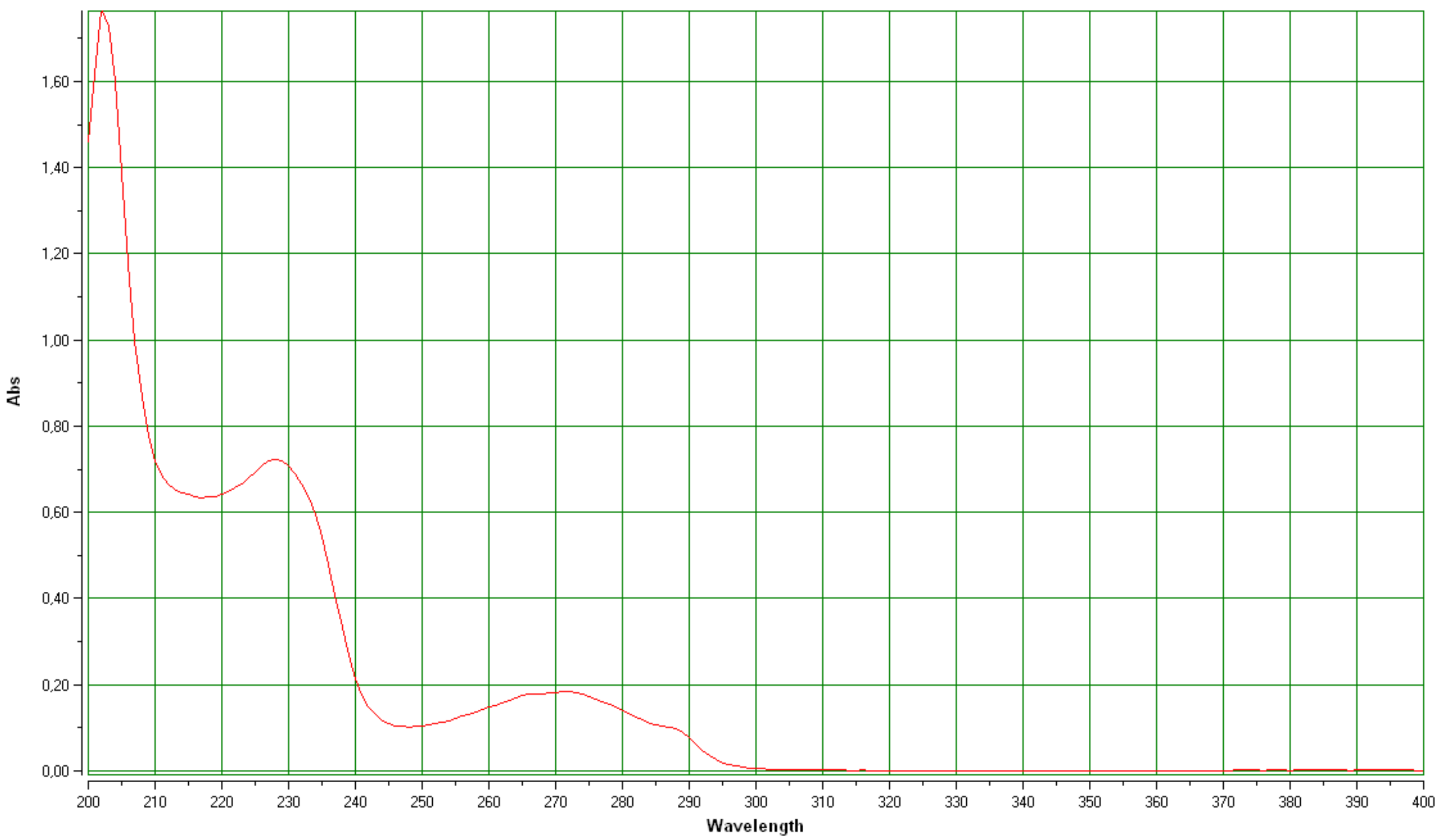

***Figure S52**. FT-IR spectrum of N-hexadecylonicotinamide 4-chloro-2-methylphenoxyacetate (**15**).*

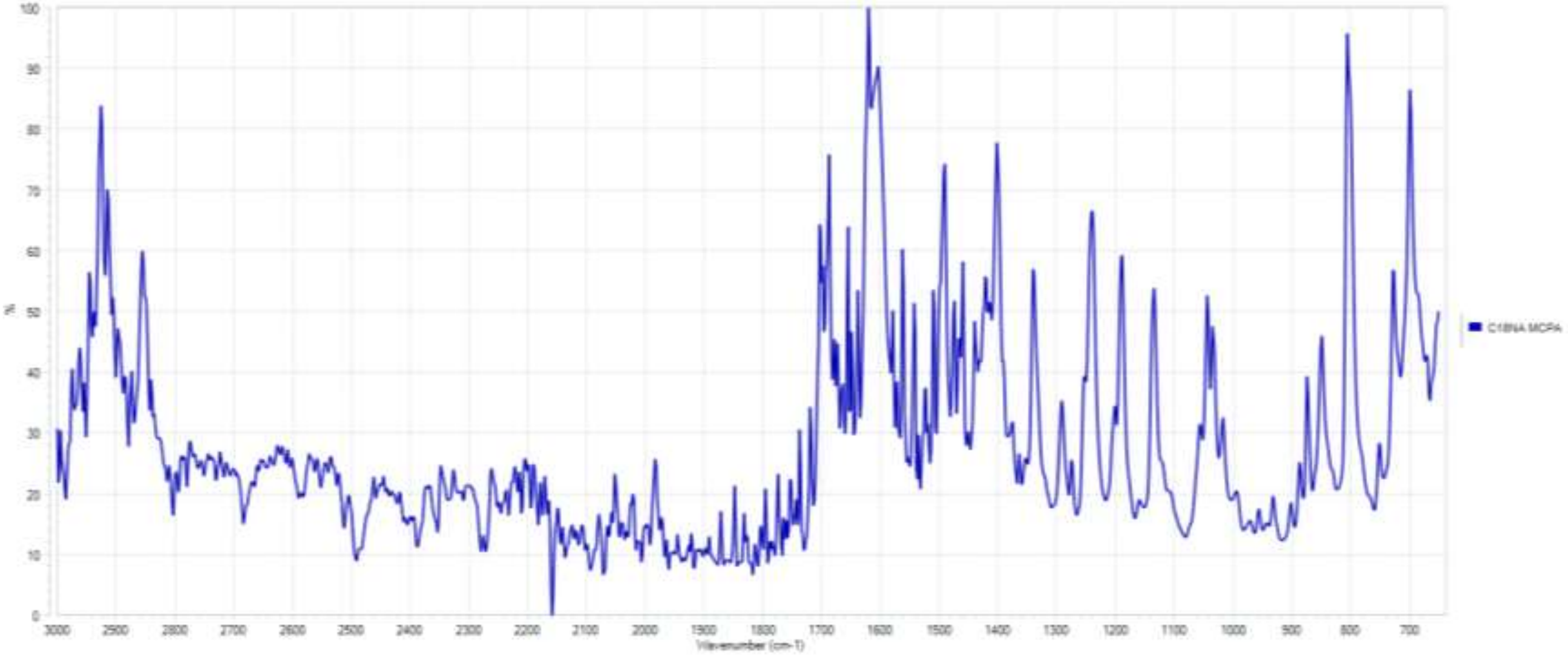

Figure S53. *^{1}H NMR spectrum of N-hexadecylonicotinamide 4-chloro-2-methylphenoxyacetate* ***(15)***.

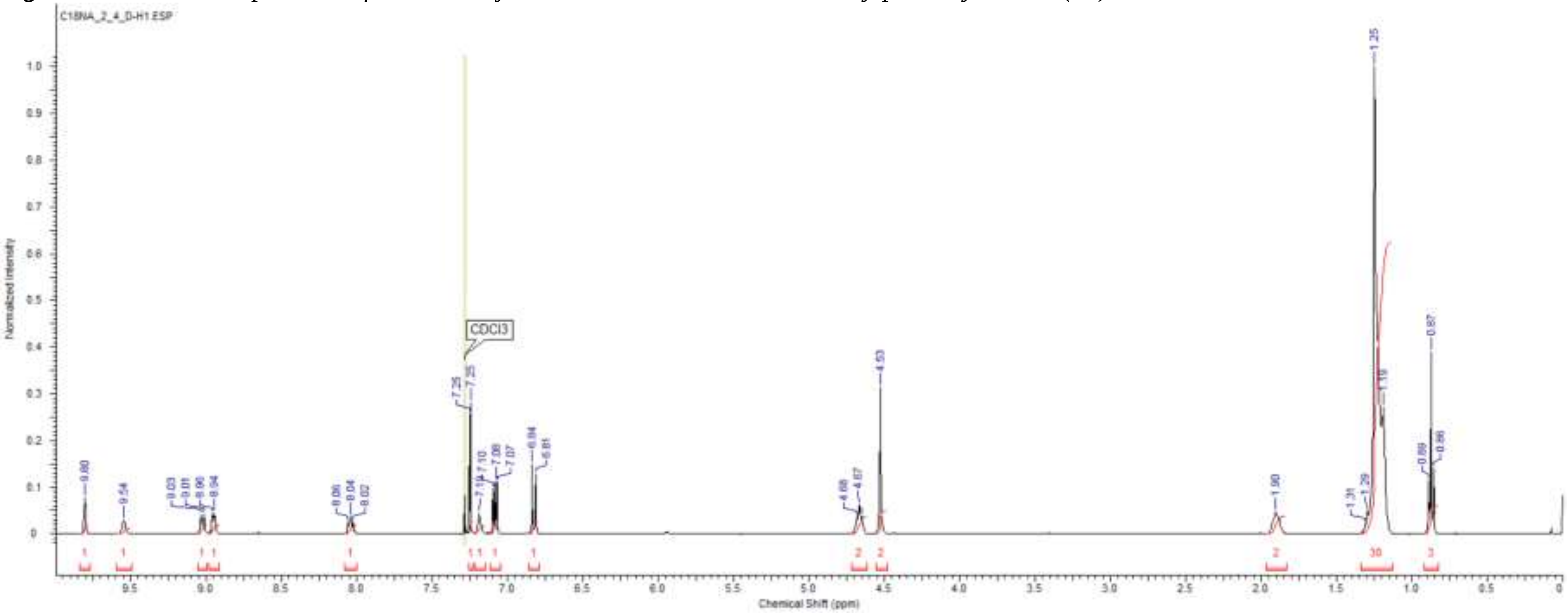

Figure S54. *^{13}C NMR spectrum of N-hexadecylonicotinamide 4-chloro-2-methylphenoxyacetate (**15**).*

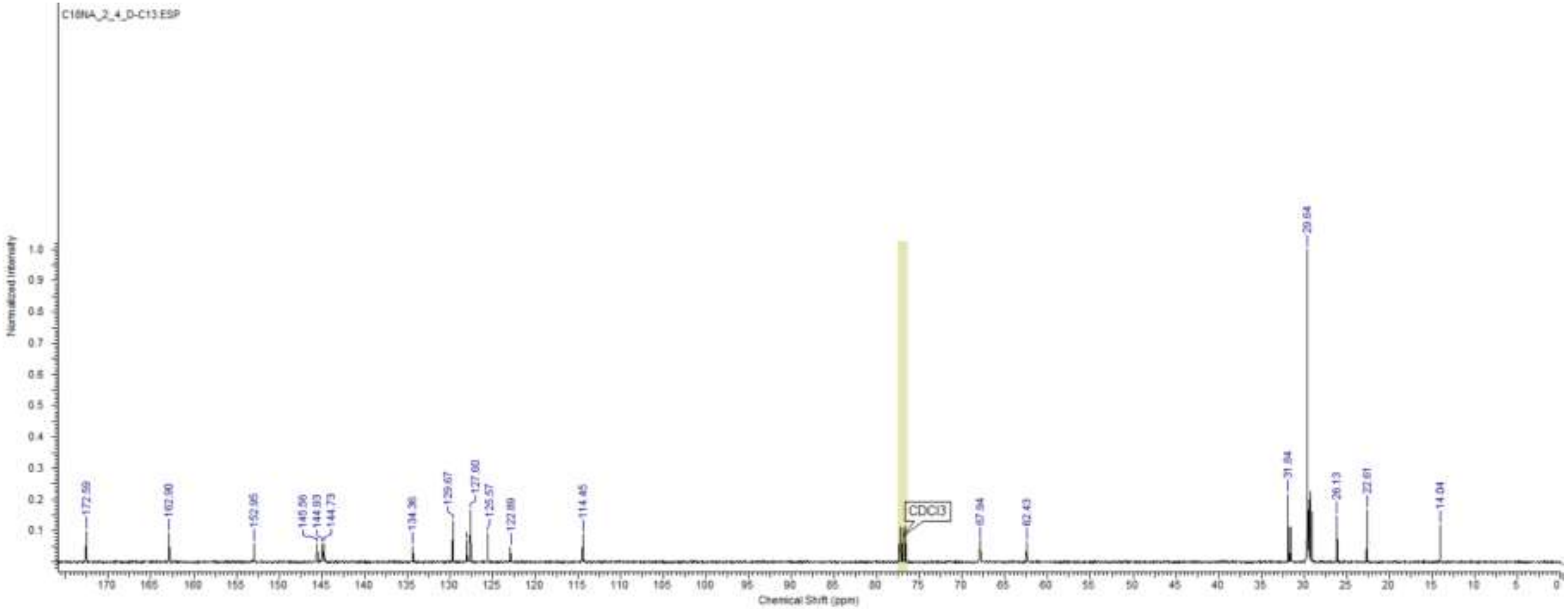

Figure S55. *The comparison between FT-IR spectra of products with bromide as the anion (**1-5**), wherein alkyl refers to decyl (**1**) (**blue line**); dodecyl (**2**) (**green line**); tetradecyl (**3**) (**red line**); hexadecyl (**4**) (**light blue line**) and octadecyl (**5**) (**pink line**)*

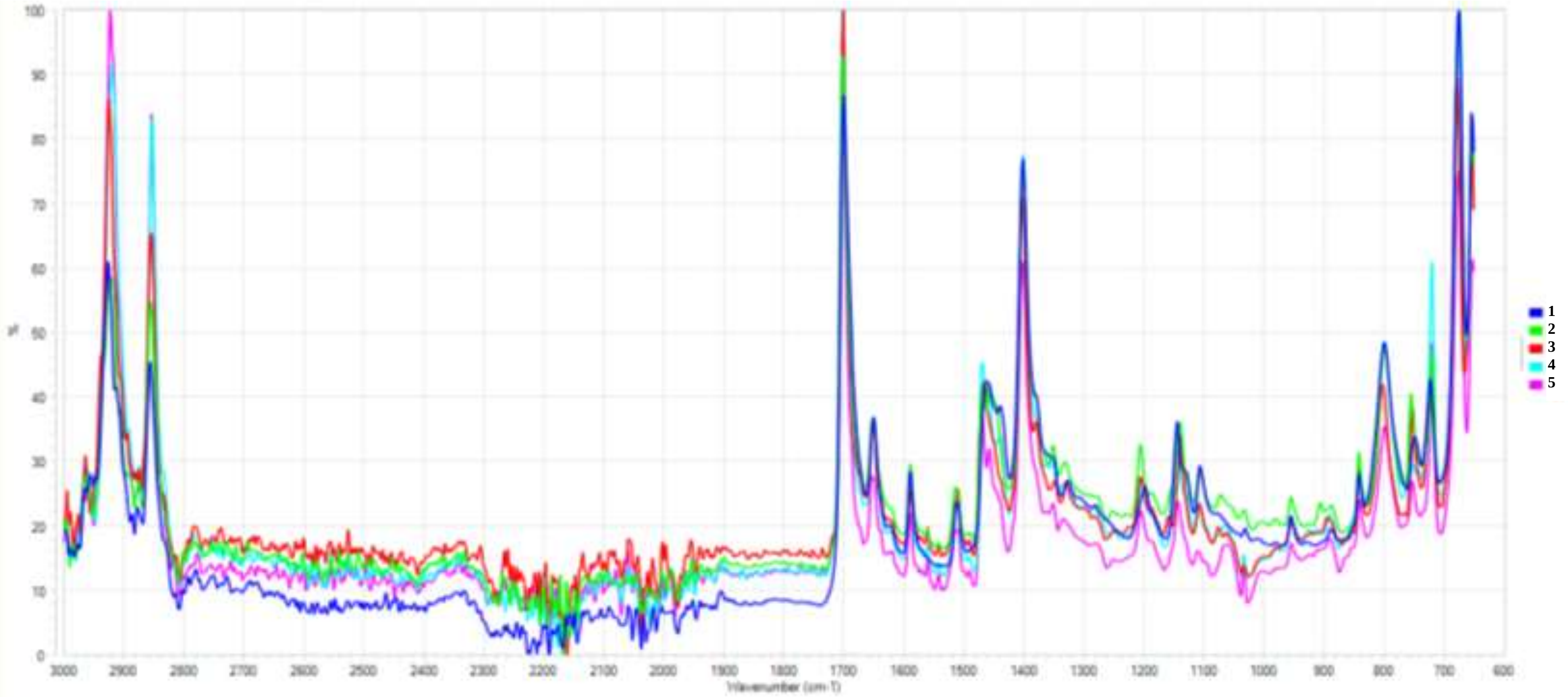

Figure S56. *The comparison between FT-IR spectra of products with 2,4-D as the anion (***6-10***), wherein alkyl refers to decyl (***6***) (***blue line***); dodecyl (***7***) (***green line***); tetradecyl (***8***) (***red line***); heksaadecyl (***9***) (***light blue line***) and octadecyl (***10***) (***pink line***)*

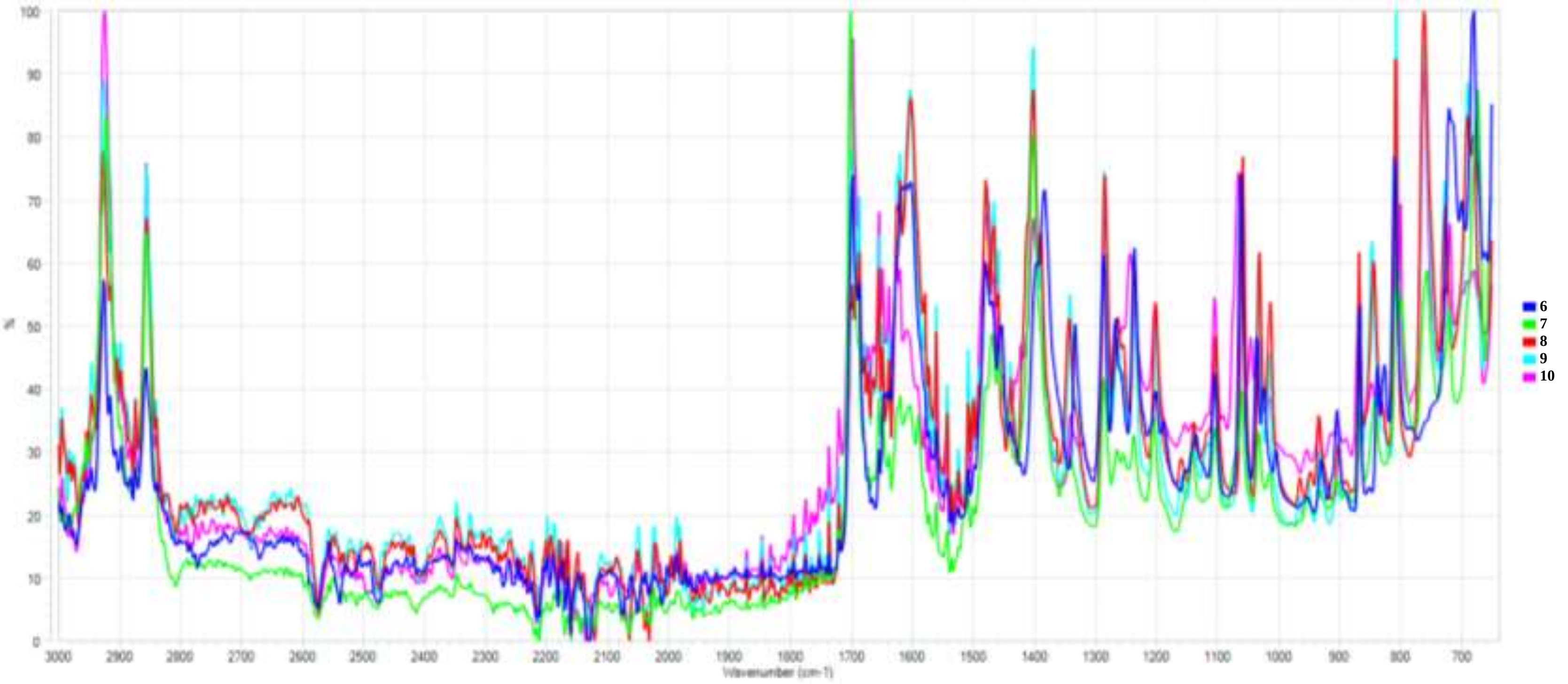

Table S2. Absorption maxima (λ_{max}) and molar absorptivities (ε) in UV spectra of the obtained products (**1**–**15**) and potassium salts of herbicidal phenoxyacids (2,4-D and MCPA) in methanol at 25 °C.

**Salt**	λ_{max1} **[nm]**	λ_{max2} **[nm]**	λ_{max3} **[nm]**	$\varepsilon_1 \cdot 10^{-4}$ $[M^{1}\ cm^{-1}]$	$\varepsilon_2 \cdot 10^{-3}$ $[M^{1}\ cm^{-1}]$	$\varepsilon_3 \cdot 10^{-3}$ $[M^{1}\ cm^{-1}]$
**1**	203	---	266	1.69	---	3.85
**2**	201	---	266	1.76	---	3.78
**3**	202	---	266	1.73	---	3.77
**4**	201	---	266	1.74	---	3.91
**5**	202	---	266	1.79	---	3.76
**6**	---	---	267	---	---	4.51
**7**	---	---	267	---	---	4.54
**8**	---	---	267	---	---	4.47
**9**	---	---	267	---	---	4.46
**10**	---	---	267	---	---	4.09
**11**	202	227	267	3.16	11.79	4.18
**12**	203	228	267	3.61	13.80	4.57
**13**	202	228	267	3.82	14.20	4.19
**14**	202	228	272	3.86	15.84	4.01
**15**	202	228	267	3.87	13.41	4.35
**[K][MCPA]**	200	229	280	3.73	9.60	1.63
**[K][2,4-D]**	201	230	285	4.41	9.30	2.09

Table S3. Absorption maxima (υ_{max}) in FTIR spectra of the obtained products (**1**–**15**) and potassium salts of herbicidal phenoxyacids (2,4-D and MCPA) at 25 °C, neat sample.

Salt	υ_{max} [cm^{-1}]
**1**	2927, 2855, 1699, 1651, 1589, 1510, 1459, 1440, 1402, 1198, 1145, 1108, 954, 842, 800, 749, 723, 675, 655
**2**	2961, 2922, 2855, 1703, 1653, 1590, 1514, 1471, 1458, 1401, 1351, 1206, 1139, 1106, 955, 907, 885, 842, 801, 755, 720, 675, 655
**3**	2965, 2925, 2854, 1701, 1650, 1588, 1509, 1465, 1403, 1204, 1138, 1107, 840, 803, 752, 717, 679, 655
**4**	2963, 2920, 2853, 1701, 651, 1590, 1514, 1471, 1401, 1204, 1143, 1106, 1063, 955, 889, 840, 801, 751, 720, 675, 653
**5**	2961, 2922, 2855, 1703, 1651, 1590, 1560, 1514, 1471, 1458, 1401, 1394, 1204, 1143, 1063, 1035, 955, 894, 842, 798, 753, 718, 675, 653
**6**	2926, 2856, 1698, 1605, 1480, 1455, 1383, 1333, 1286, 1267, 1236, 1203, 1135, 1106, 1063, 1034, 1024, 1005, 929, 904, 867, 838, 828, 811, 722, 681, 650
**7**	2924, 2856, 1702, 1655, 1619, 1606, 1590, 1563, 1509, 1471, 1460, 1401, 1343, 1287, 1266, 1237, 1204, 1143, 1105, 1060, 1033, 1015, 954, 934, 905, 869, 842, 806, 799, 759, 721, 676, 651
**8**	2926, 2856, 1686, 1655, 1603, 1563, 1543, 1509, 1480, 1466, 1439, 1401, 1343, 1284, 1264, 1235, 1201, 1161, 1138, 1103, 1058, 1031, 1015, 965, 948, 932, 903, 869, 844, 808, 761, 728, 689, 651
**9**	2926, 2856, 1702, 1686, 1655, 1606, 1563, 1543, 1525, 1509, 1480, 1466, 1460, 1439, 1403, 1343, 1287, 1264, 1237, 1201, 1159, 1138, 1105, 1060, 1033, 1015, 968, 952, 932, 898, 869, 847, 806, 761, 728, 689, 651
**10**	2926, 2856, 1698, 1655, 1639, 1626, 1563, 1543, 1509, 1475, 1401, 1341, 1284, 1264, 1244, 1204, 1105, 1067, 1033, 1015, 952, 934, 905, 869, 847, 808, 802, 761, 719, 680, 651
**11**	2963, 2930, 2858, 1703, 1687, 1656, 1605, 1561, 1492, 1468, 1459, 1438, 1401, 1357, 1338, 1292, 1274, 1239, 1190, 1134, 1058, 1044, 1018, 995, 932, 886, 874, 849, 805, 726, 700, 650
**12**	2963, 2930, 2914, 2875, 2858, 1703, 1687, 1656, 1603, 1561, 1543, 1524, 1510, 1492, 1475, 1459, 1438, 1401, 1338, 1292, 1276, 1253, 1239, 1204, 1190, 1134, 1041, 1018, 993, 972, 956, 932, 905, 874, 849, 805, 728, 698, 654
**13**	2963, 2928, 2856, 1703, 1687, 1656, 1603, 1561, 1543, 1492, 1468, 1401, 1341, 1290, 1274, 1239, 1190, 1134, 1074, 1041, 1018, 993, 953, 930, 902, 886, 874, 849, 800, 749, 728, 698, 684, 652
**14**	2928, 2856, 1703, 1687, 1656, 1603, 1561, 1543, 1524, 1492, 1466, 1401, 1341, 1292, 1276, 1239, 1190, 1132, 1044, 1018, 933, 972, 956, 932, 886, 874, 851, 805, 749, 728, 698, 684, 652
**15**	2963, 2944, 2923, 2914, 2856, 1703, 1687, 1656, 1619, 1605, 1561, 1543, 1524, 1510, 1492, 1475, 1459, 1422, 1401, 1341, 1292, 1276, 1241, 1190, 1137, 1044, 1034, 932, 886, 874, 851, 805, 751, 728, 698, 652
**[K][2,4-D]**	2945, 2931, 1737, 1721, 1705, 1686, 1656, 1638, 1618, 1563, 1543, 1510, 1482, 1460, 1425, 1348, 1282, 1267, 1243, 1160, 1108, 1067, 1045, 869, 800, 759, 687, 668
**[K][MCPA]**	2946, 2929, 2906, 1738, 1721, 1704, 1688, 1654, 1638, 1600, 1563, 1544, 1511, 1494, 1459, 1427, 1404, 1371, 1342, 1294, 1275, 1240, 1192, 1140, 1063, 1034, 994, 944, 925, 892, 875, 800, 694, 684, 661

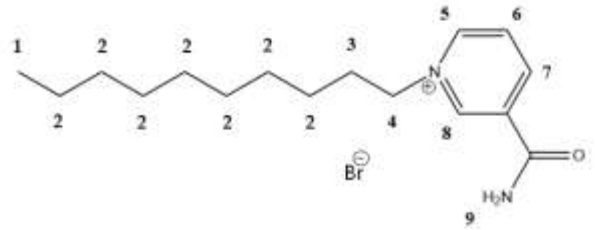

Table S4. Analysis of ^{1}H NMR spectrum of *N*-decylonicotinamide bromide (**1**).

Proton	Chemical shift [ppm]	Integration	Multiplicity	Coupling constant [Hz]
1	0.85	triplet	3H	6.97
2	1.27	multiplet	14H	-
3	1.99	multiplet	2H	-
4	4.74	triplet	2H	7.46
5	9.39	doublet	1H	6.14
6	8.33	triplet	1H	6.30
7	9.03	doublet	1H	8.13
8	9.69	singlet	1H	-
9	8.22 ; 8.70	singlet	1H	-

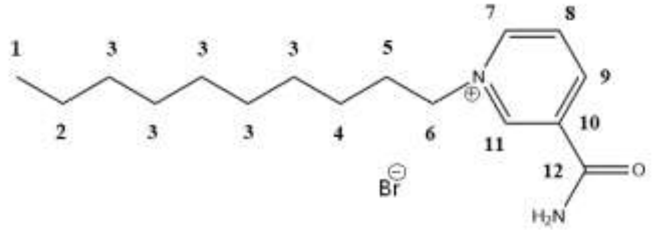

Table S5. Analysis of ^{13}C NMR spectrum of *N*-decylonicotinamide bromide (**1**).

Carbon atom	Chemical shift [ppm]	Carbon atom	Chemical shift [ppm]
1	13.93	7	144.68
2	22.08	8	127.85
3	28.65	9	146.41
4	25.38	10	133.63
5	31.25	11	143.48
6	60.98	12	162.76

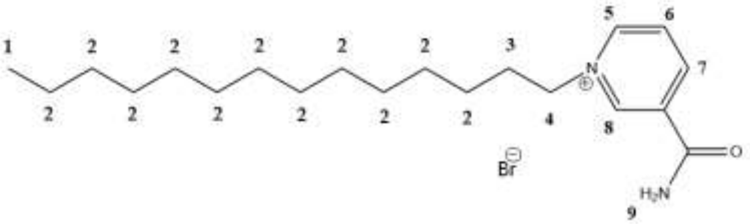

Table S6. Analysis of ^{1}H NMR spectrum of *N*-tetradecylonicotinamide bromide (**3**).

Proton	Chemical shift [ppm]	Integration	Multiplicity	Coupling constant [Hz]
1	0.86	triplet	3H	6.80
2	1.29	multiplet	22H	-
3	1.98	multiplet	2H	-
4	4.72	triplet	2H	7.46
5	9.35	doublet	1H	5.81
6	8.34	triplet	1H	6.47
7	9.02	doublet	1H	7.96
8	9.66	singlet	1H	-
9	8.21 ; 8.69	singlet	1H	-

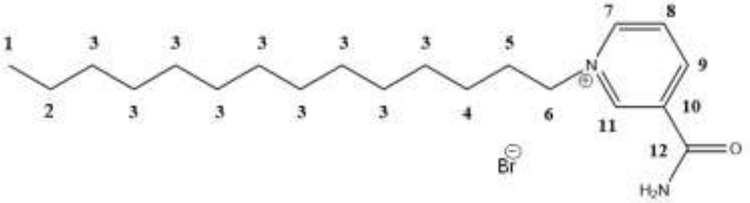

Table S7. Analysis of ^{13}C NMR spectrum of *N*-tetradecylonicotinamide bromide (**3**).

Carbon atom	Chemical shift [ppm]	Carbon atom	Chemical shift [ppm]
1	13.92	7	143.40
2	22.08	8	127.84
3	29.01	9	146.39
4	25.39	10	133.65
5	31.28	11	144.70
6	61.01	12	162.76

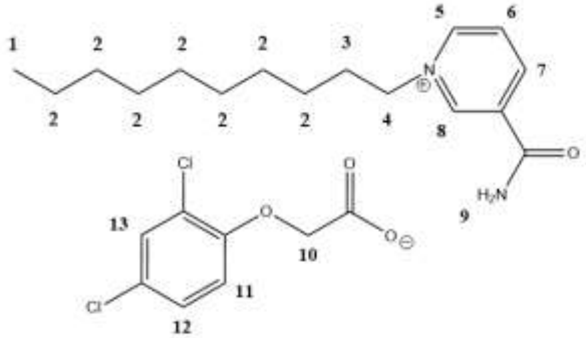

Table S8. Analysis of ^{1}H NMR spectrum of *N*-decylonicotinamide 2,4-dichlorophenoxyacetate (**6**).

Proton	Chemical shift [ppm]	Integration	Multiplicity	Coupling constant [Hz]
1	0.86	triplet	3H	6.83
2	1.22	multiplet	14H	-
3	1.94	multiplet	2H	-
4	4.76	triplet	2H	7.43
5	9.20	doublet	1H	6.02
6	8.06	triplet	1H	6.12
7	9.10	doublet	1H	8.13
8	10.12	singlet	1H	-
9	7.37 ; 10.00	singlet	1H	-
10	4.51	singlet	2H	-
11	6.89	doublet	1H	8.93
12	7.09	doublet	1H	8.83
13	7.23	singlet	1H	-

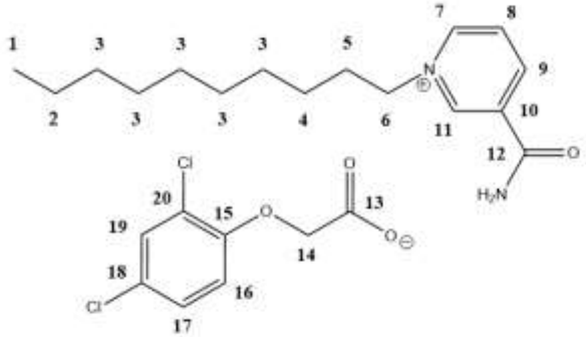

Table S9. Analysis of ^{13}C NMR spectrum of *N*-decylonicotinamide 2,4-dichlorophenoxyacetate (**6**).

Carbon atom	Chemical shift [ppm]	Carbon atom	Chemical shift [ppm]	Carbon atom	Chemical shift [ppm]
1	13.94	8	127.82	15	153.26
2	22.48	9	145.81	16	114.43
3	29.24	10	134.40	17	127.50
4	26.06	11	144.78	18	125.00
5	31.66	12	162.73	19	129.43
6	62.19	13	172.64	20	122.58
7	145.16	14	68.82		

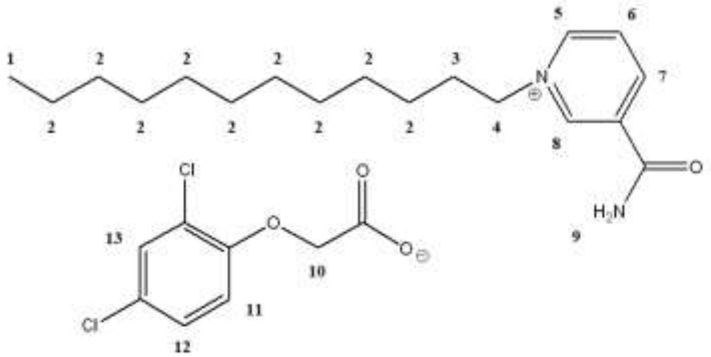

Table S10. Analysis of ^{1}H NMR spectrum of *N*-dodecylonicotinamide 2,4-dichlorophenoxyacetate (**7**).

Proton	Chemical shift [ppm]	Integration	Multiplicity	Coupling constant [Hz]
1	0.85	triplet	3H	6.88
2	1.25	multiplet	18H	-
3	1.94	multiplet	2H	-
4	4.70	triplet	2H	7.30
5	9.33	doublet	1H	6.05
6	8.28	triplet	1H	6.05
7	9.03	doublet	1H	8.96
8	9.76	singlet	1H	-
9	8.17 ; 9.04	singlet	1H	-
10	4.45	singlet	2H	-
11	6.92	doublet	1H	8.69
12	7.26	doublet of doublets	1H	8.96 ; 2.50
13	7.45	doublet	1H	2.50

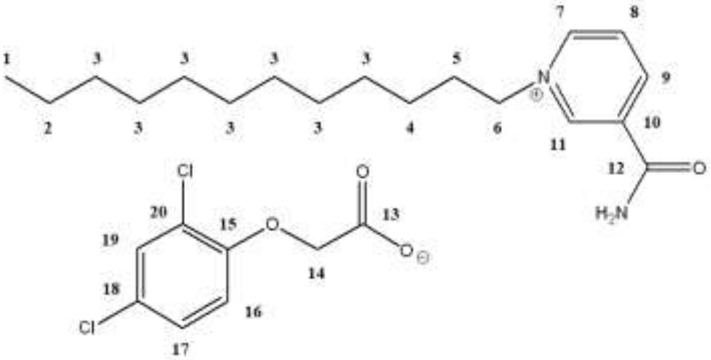

Table S11. Analysis of ^{13}C NMR spectrum of *N*-dodecylonicotinamide 2,4-dichlorophenoxyacetate (**7**).

Carbon atom	Chemical shift [ppm]	Carbon atom	Chemical shift [ppm]	Carbon atom	Chemical shift [ppm]
1	13.96	8	127.83	15	153.31
2	22.12	9	143.59	16	114.85
3	29.04	10	133.80	17	127.59
4	25.44	11	144.81	18	123.51
5	31.32	12	162.77	19	128.86
6	61.00	13	169.86	20	121.81
7	146.40	14	67.61		

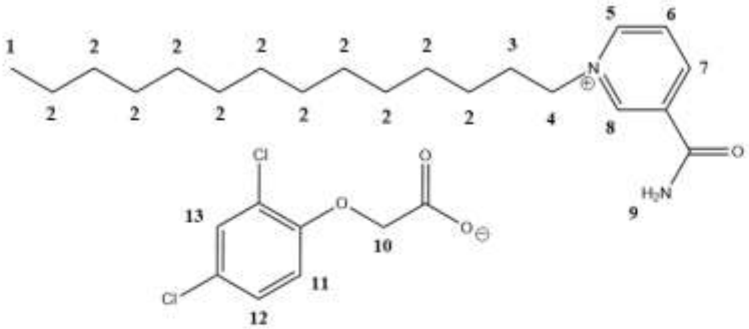

Table S12. Analysis of ^{1}H NMR spectrum of *N*-tetradecylonicotinamide 2,4-dichlorophenoxyacetate (**8**).

Proton	Chemical shift [ppm]	Integration	Multiplicity	Coupling constant [Hz]
1	0.87	triplet	3H	6.73
2	1.25	multiplet	22H	-
3	1.95	multiplet	2H	-
4	4.75	triplet	2H	7.43
5	9.11	doublet	1H	8.03
6	8.01	triplet	1H	6.12
7	8.95	doublet	1H	6.02
8	10.30	singlet	1H	-
9	6.68 ; 10.17	singlet	1H	-
10	4.52	singlet	2H	-
11	6.87	doublet	1H	8.93
12	7.09	doublet of doublets	1H	8.83 ; 2.61
13	7.28	singlet	1H	-

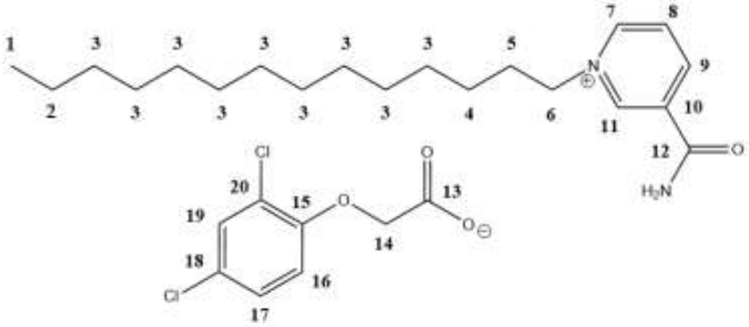

Table S13. Analysis of ^{13}C NMR spectrum of *N*-tetradecylonicotinamide 2,4-dichlorophenoxyacetate (**8**).

Carbon atom	Chemical shift [ppm]	Carbon atom	Chemical shift [ppm]	Carbon atom	Chemical shift [ppm]
1	14.08	8	127.64	15	153.41
2	22.64	9	145.17	16	114.44
3	29.59	10	134.78	17	127.51
4	26.13	11	145.71	18	125.13
5	31.87	12	162.57	19	129.61
6	62.41	13	172.95	20	122.83
7	145.17	14	68.83		

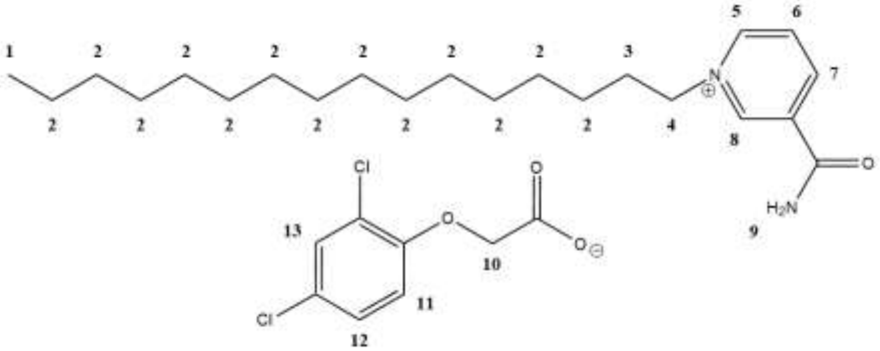

Table S14. Analysis of ^{1}H NMR spectrum of *N*-hexadecylonicotinamide 2,4-dichlorophenoxyacetate (**9**).

Proton	Chemical shift [ppm]	Integration	Multiplicity	Coupling constant [Hz]
1	0.88	triplet	3H	6.77
2	1.24	multiplet	26H	-
3	1.96	multiplet	2H	-
4	4.79	triplet	2H	7.18
5	9.10	doublet	1H	6.97
6	8.07	triplet	1H	6.77
7	9.12	doublet	1H	6.97
8	10.11	singlet	1H	-
9	7.14 ; 9.87	singlet	1H	-
10	4.51	singlet	2H	-
11	6.88	doublet	1H	8.82
12	7.08	doublet	1H	9.02
13	7.24	singlet	1H	-

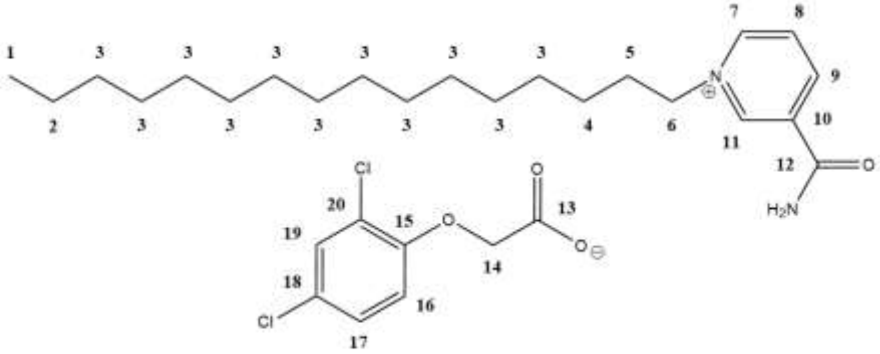

Table S15. Analysis of ^{13}C NMR spectrum of *N*-hexadecylonicotinamide 2,4-dichlorophenoxyacetate (**9**).

Carbon atom	Chemical shift [ppm]	Carbon atom	Chemical shift [ppm]	Carbon atom	Chemical shift [ppm]
1	14.06	8	127.86	15	153.28
2	22.60	9	145.61	16	114.40
3	29.62	10	134.41	17	124.55
4	26.13	11	145.27	18	125.09
5	31.84	12	162.77	19	129.54
6	62.34	13	172.87	20	122.68
7	144.93	14	68.78		

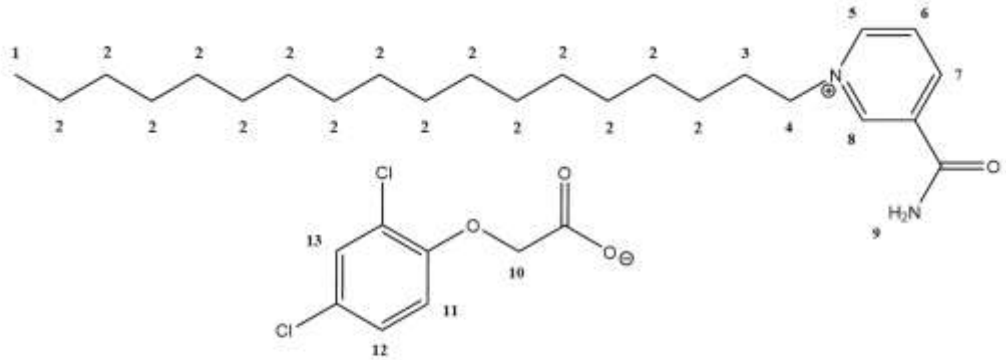

Table S16. Analysis of ^{1}H NMR spectrum of *N*-octadecylonicotinamide 2,4-dichlorophenoxyacetate (**10**).

Proton	Chemical shift [ppm]	Integration	Multiplicity	Coupling constant [Hz]
1	0.87	triplet	3H	6.73
2	1.23	multiplet	30H	-
3	1.90	multiplet	2H	-
4	4.66	triplet	2H	7.23
5	9.03	doublet	1H	7.93
6	8.04	triplet	1H	6.73
7	8.94	doublet	1H	5.82
8	9.81	singlet	1H	-
9	7.18 ; 9.55	singlet	1H	-
10	4.52	singlet	2H.	-
11	6.82	doublet	1H	8.83
12	7.08	doublet of doublets	1H	8.83 ; 2.61
13	7.24	doublet	1H	2.61

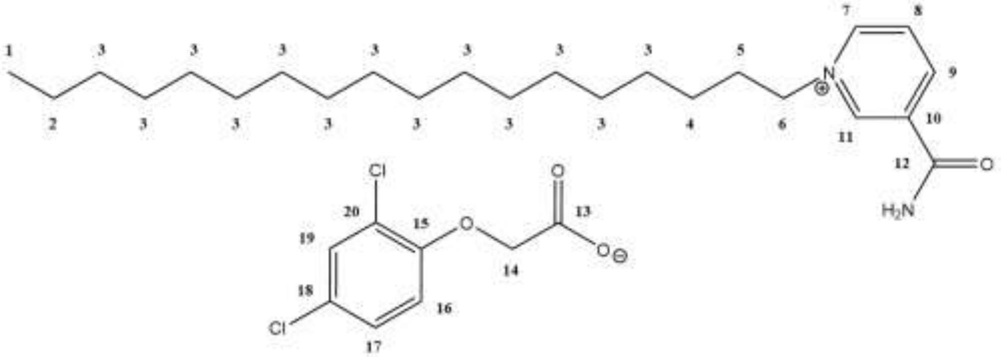

Table S17. Analysis of ^{13}C NMR spectrum of *N*-octadecylonicotinamide 2,4-dichlorophenoxyacetate (**10**).

Carbon atom	Chemical shift [ppm]	Carbon atom	Chemical shift [ppm]	Carbon atom	Chemical shift [ppm]
1	14.04	8	128.04	15	152.95
2	22.61	9	145.56	16	114.45
3	29.64	10	134.36	17	127.60
4	26.13	11	144.93	18	125.57
5	31.84	12	162.90	19	129.67
6	62.43	13	172.59	20	122.89
7	144.73	14	67.94		

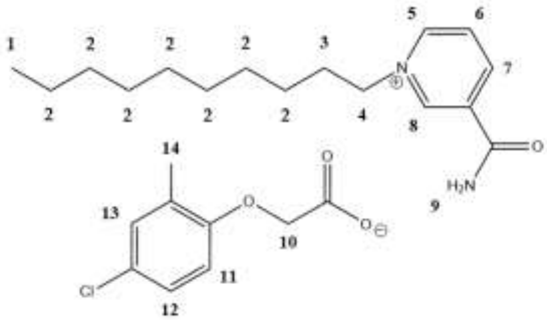

Table S18. Analysis of ^{1}H NMR spectrum of *N*-decylonicotinamide 4-chloro-2-methylphenoxyacetate (**11**).

Proton	Chemical shift [ppm]	Integration	Multiplicity	Coupling constant [Hz]
1	0.86	triplet	3H	6.83
2	1.23	multiplet	14H	-
3	1.92	multiplet	2H	-
4	4.69	triplet	2H	7.43
5	9.07	doublet	1H	8.03
6	7.99	triplet	1H	6.22
7	8.99	doublet	1H	6.02
8	10.06	singlet	1H	-
9	7.48 ; 9.99	singlet	1H	-
10	4.43	singlet	2H	-
11	6.69	doublet	1H	8.63
12	6.90	doublet of doublets	1H	8.63 ; 2.41
13	6.95	doublet	1H	2.61
14	2.14	singlet	3H	-

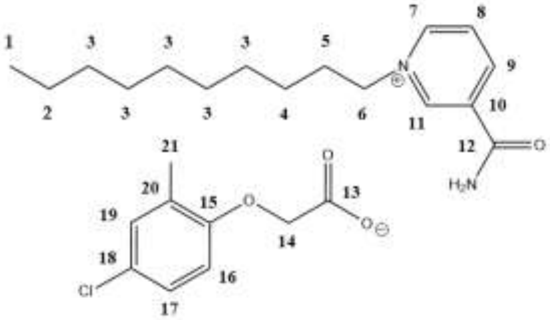

Table S19. Analysis of ^{13}C NMR spectrum of *N*-decylonicotinamide 4-chloro-2-methylphenoxyacetate (**11**).

Carbon atom	Chemical shift [ppm]	Carbon atom	Chemical shift [ppm]	Carbon atom	Chemical shift [ppm]
1	13.93	8	127.81	15	155.75
2	22.47	9	145.36	16	112.65
3	29.30	10	134.33	17	128.35
4	26.03	11	144.74	18	126.02
5	31.64	12	162.81	19	129.96
6	62.20	13	174.03	20	124.29
7	145.08	14	68.44	21	16.20

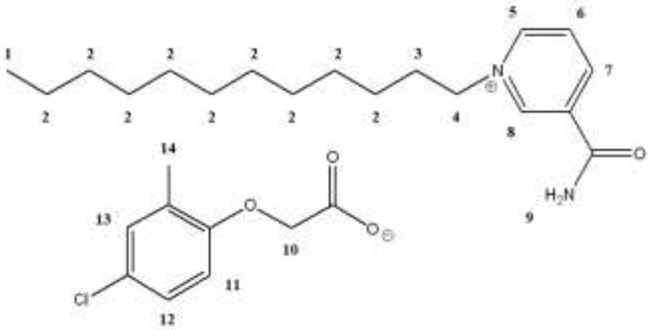

Table S20. Analysis of ^{1}H NMR spectrum of *N*-dodecylonicotinamide 4-chloro-2-methylphenoxyacetate (**12**).

Proton	Chemical shift [ppm]	Integration	Multiplicity	Coupling constant [Hz]
1	0.84	triplet	3H	6.70
2	1.23	multiplet	18H	-
3	1.90	multiplet	2H	-
4	4.66	triplet	2H	7.15
5	9.31	doublet	1H	5.71
6	8.21	triplet	1H	6.77
7	9.03	doublet	1H	7.99
8	9.91	singlet	1H	-
9	8.12 ; 9.56	singlet	1H	-
10	4.20	singlet	2H	-
11	6.68	doublet	1H	8.68
12	7.02	doublet	1H	8.68
13	7.09	singlet	1H	-
14	2.12	singlet	3H	-

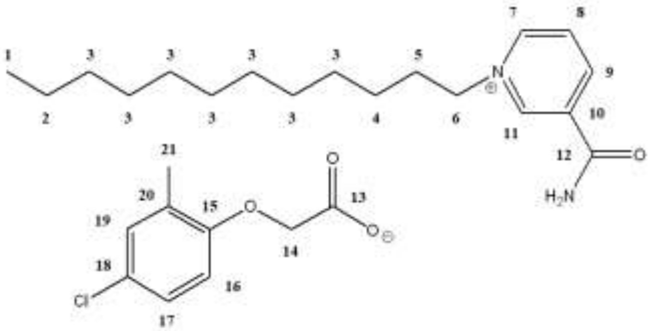

Table S21. Analysis of ^{13}C NMR spectrum of *N*-dodecylonicotinamide 4-chloro-2-methylphenoxyacetate (**12**).

Carbon atom	Chemical shift [ppm]	Carbon atom	Chemical shift [ppm]	Carbon atom	Chemical shift [ppm]
1	14.00	8	127.81	15	156.14
2	22.15	9	146.39	16	112.79
3	29.07	10	133.90	17	127.81
4	25.48	11	144.94	18	125.89
5	31.34	12	162.80	19	129.40
6	60.98	13	170.45	20	122.56
7	143.77	14	68.29	21	16.07

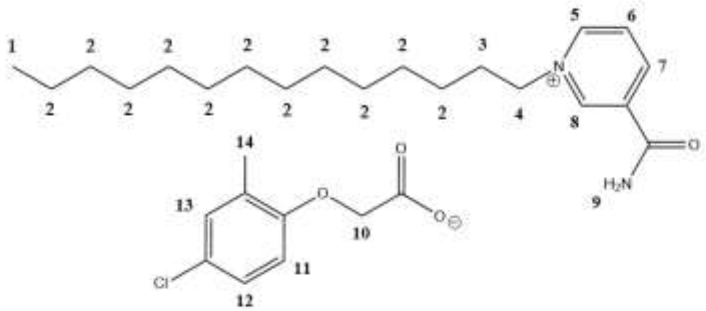

Table S22. Analysis of ^{1}H NMR spectrum of *N*-tetradecylonicotinamide 4-chloro-2-methylphenoxyacetate (**13**).

Proton	Chemical shift [ppm]	Integration	Multiplicity	Coupling constant [Hz]
1	0.82	triplet	3H	7.04
2	1.21	multiplet	22H	-
3	1.88	multiplet	2H	-
4	4.62	triplet	2H	7.46
5	9.24	doublet	1H	6.19
6	8.19	triplet	1H	6.19
7	8.98	doublet	1H	8.31
8	9.81	singlet	1H	-
9	8.05 ; 9.34	singlet	1H	-
10	4.25	singlet	2H	-
11	6.67	doublet	1H	8.73
12	7.02	doublet of doublets	1H	8.73 ; 2.82
13	7.08	doublet	1H	2.67
14	2.11	singlet	3H	-

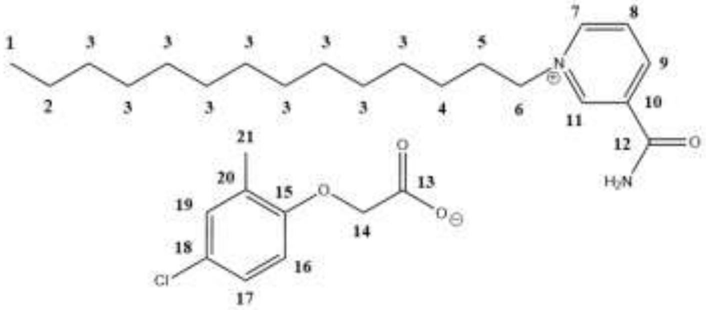

Table S23. Analysis of ^{13}C NMR spectrum of *N*-tetradecylonicotinamide 4-chloro-2-methylphenoxyacetate (**13**).

Carbon atom	Chemical shift [ppm]	Carbon atom	Chemical shift [ppm]	Carbon atom	Chemical shift [ppm]
1	13.91	8	127.89	15	155.88
2	22.09	9	146.31	16	112.77
3	29.01	10	133.89	17	127.75
4	25.43	11	144.87	18	125.91
5	31.29	12	162.73	19	129.45
6	60.97	13	170.48	20	122.81
7	143.69	14	67.68	21	15.95

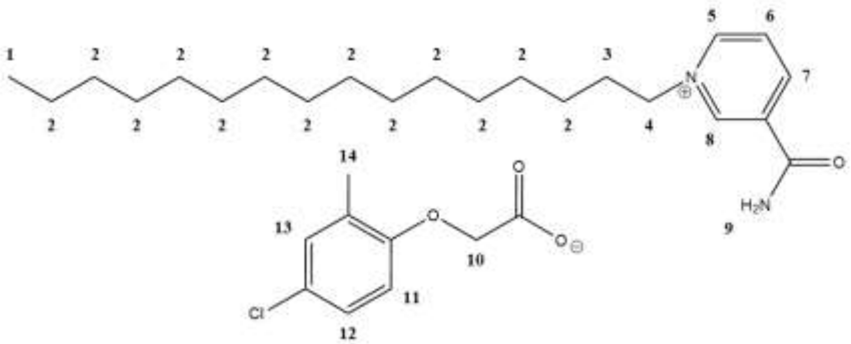

Table S24. Analysis of ^{1}H NMR spectrum of *N*-hexadecylonicotinamide 4-chloro-2-methylphenoxyacetate (**14**).

Proton	Chemical shift [ppm]	Integration	Multiplicity	Coupling constant [Hz]
1	0.85	triplet	3H	7.03
2	1.23	multiplet	26H	-
3	1.90	multiplet	2H	-
4	4.67	triplet	2H	7.54
5	9.29	doublet	1H	6.00
6	8.21	triplet	1H	6.17
7	9.03	doublet	1H	8.05
8	9.91	singlet	1H	-
9	8.11 ; 9.52	singlet	1H	-
10	4.30	singlet	2H	-
11	6.72	doublet	1H	8.74
12	7.07	doublet of doublets	1H	8.74 ; 2.74
13	7.13	doublet	1H	2.40
14	2.14	singlet	3H	-

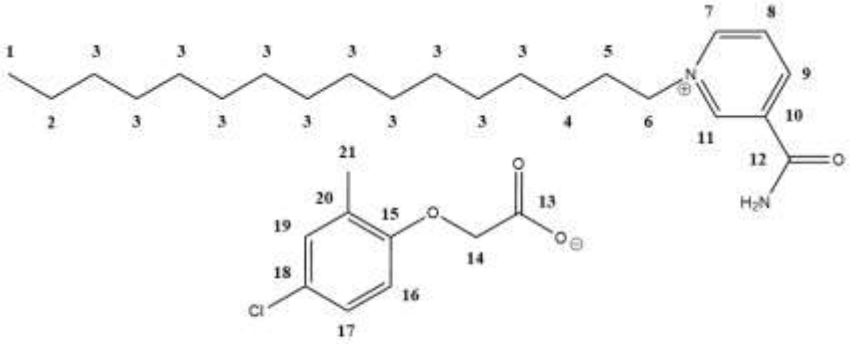

Table S25. Analysis of ^{13}C NMR spectrum of *N*-hexadecylonicotinamide 4-chloro-2-methylphenoxyacetate (**14**).

Carbon atom	Chemical shift [ppm]	Carbon atom	Chemical shift [ppm]	Carbon atom	Chemical shift [ppm]
1	13.90	8	127.72	15	155.84
2	22.08	9	146.32	16	112.80
3	29.05	10	133.89	17	127.89
4	25.45	11	143.74	18	125.91
5	31.29	12	162.71	19	129.46
6	60.92	13	170.54	20	122.87
7	144.90	14	67.58	21	15.95

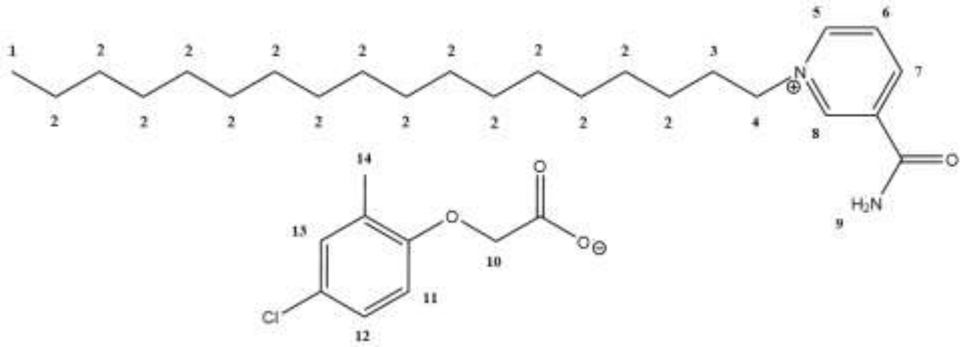

Table S26. Analysis of ^{1}H NMR spectrum of *N*-octadecylonicotinamide 4-chloro-2-methylphenoxyacetate (**15**).

Proton	Chemical shift [ppm]	Integration	Multiplicity	Coupling constant [Hz]
1	0.86	triplet	3H	7.08
2	1.24	multiplet	30H	-
3	1.92	multiplet	2H	-
4	4.65	triplet	2H	7.49
5	9.06	doublet	1H	6.05
6	7.92	triplet	1H	6.15
7	8.62	doublet	1H	8.10
8	10.34	singlet	1H	-
9	6.31 ; 10.25	singlet	1H	-
10	4.49	singlet	2H	-
11	6.66	doublet	1H	8.72
12	6.94	doublet of doublets	1H	8.72 ; 2.67
13	7.03	doublet	1H	2.56
14	2.20	singlet	3H	-

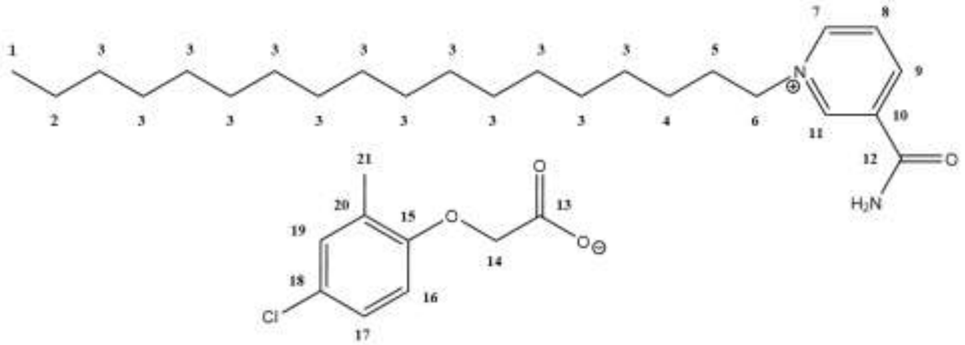

Table S27. Analysis of ^{13}C NMR spectrum of *N*-octadecylonicotinamide 4-chloro-2-methylphenoxyacetate (**15**).

Carbon atom	Chemical shift [ppm]	Carbon atom	Chemical shift [ppm]	Carbon atom	Chemical shift [ppm]
1	14.11	8	127.45	15	155.64
2	22.66	9	145.87	16	112.45
3	29.67	10	134.97	17	128.70
4	26.09	11	145.26	18	126.05
5	31.89	12	162.46	19	130.24
6	62.47	13	173.90	20	124.70
7	144.32	14	67.74	21	16.31

Table S28. Solubility of the salts comprising *N*-alkylnicotinamide as the cation and bromide (**1**–**5**), 2,4-D (**6**–**10**) or MCPA (**11**–**15**) as the anion at 25 °C.

**No**	**Water**	**Methanol**	**DMSO**	**Acetonitrile**	**Acetone**	**Chloroform**	**Isopropanol**	**Ethyl acetate**	**Toluene**	**Hexane**
	9.0[a]	6.6	6.5	6.2	5.4	4.4	4.3	4.3	2.3	0.0
**1**	+	+	+	–	–	–	–	–	–	–
**2**	+	+	+	–	–	–	–	–	–	–
**3**	–	+	+	–	–	–	–	–	–	–
**4**	–	+	+	–	–	–	–	–	–	–
**5**	–	+	+	–	–	–	–	–	–	–
**6**	+	+	+	–	–	+	+	–	–	–
**7**	+	+	+	–	–	+	+	–	–	–
**8**	+	+	+	–	–	+	+	–	–	–
**9**	+	+	+	–	–	+	+	–	–	–
**10**	±	+	–	–	–	+	+	–	–	–
**11**	+	+	+	–	–	+	+	–	–	–
**12**	+	+	+	–	–	+	+	–	–	–
**13**	+	+	+	–	–	+	+	–	–	–
**14**	±	+	+	–	–	+	+		–	–
**15**	±	+	–	–	–	+	+	–	–	–

[a] Snyder polarity index; "+" ready solubility; "±" limited solubility; "-" low solubility

Table S29. Values of logarithm of octanol-water partition coefficient for products containing *N*-alkylnicotinamide as the cation and bromide (**1-5**), 2,4-D (**6-10**) or MCPA (**11-15**) as the anion at 25 °C.

**Salt**	**Log K_{OW}**	**SD**
**1**	0.070	0.044
**2**	1.231	0.083
**3**	1.662	0.054
**4**	2.013	0.080
**5**	2.163	0.045
**6**	1.034	0.087
**7**	1.253	0.089
**8**	1.994	0.090
**9**	1.810	0.077
**10**	2.232	0.082
**11**	1.156	0.076
**12**	1.520	0.082
**13**	1.605	0.068
**14**	0.843	0.055
**15**	0.323	0.081

Figure S57. Scheme of an installation for determining the volatility of solutions of herbicides.

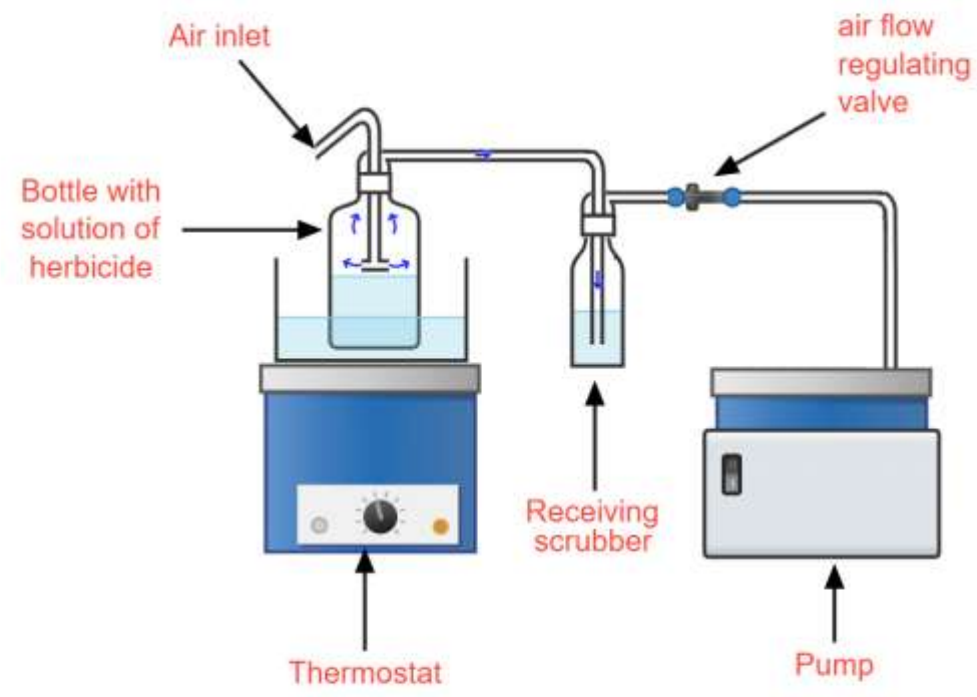

Figure S58. *Curves for determination of LOD (limit of detection) for 2,4-D (A) and MCPA (B) in water.*

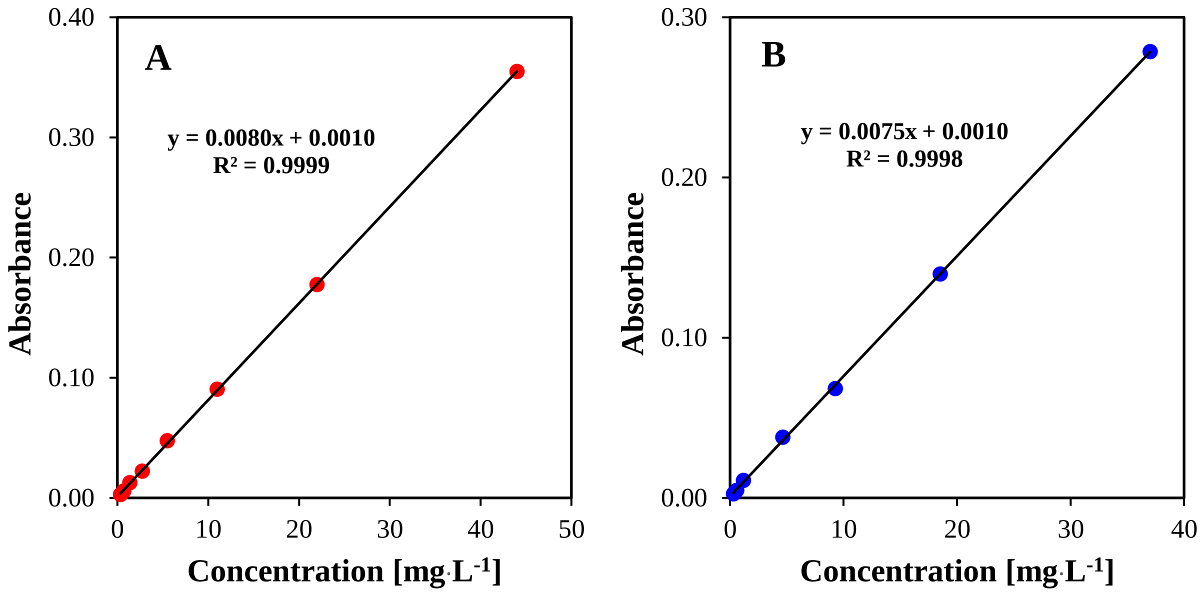

Figure S59. *UV spectra of the solutions in the receiving scrubber after the experiments.*

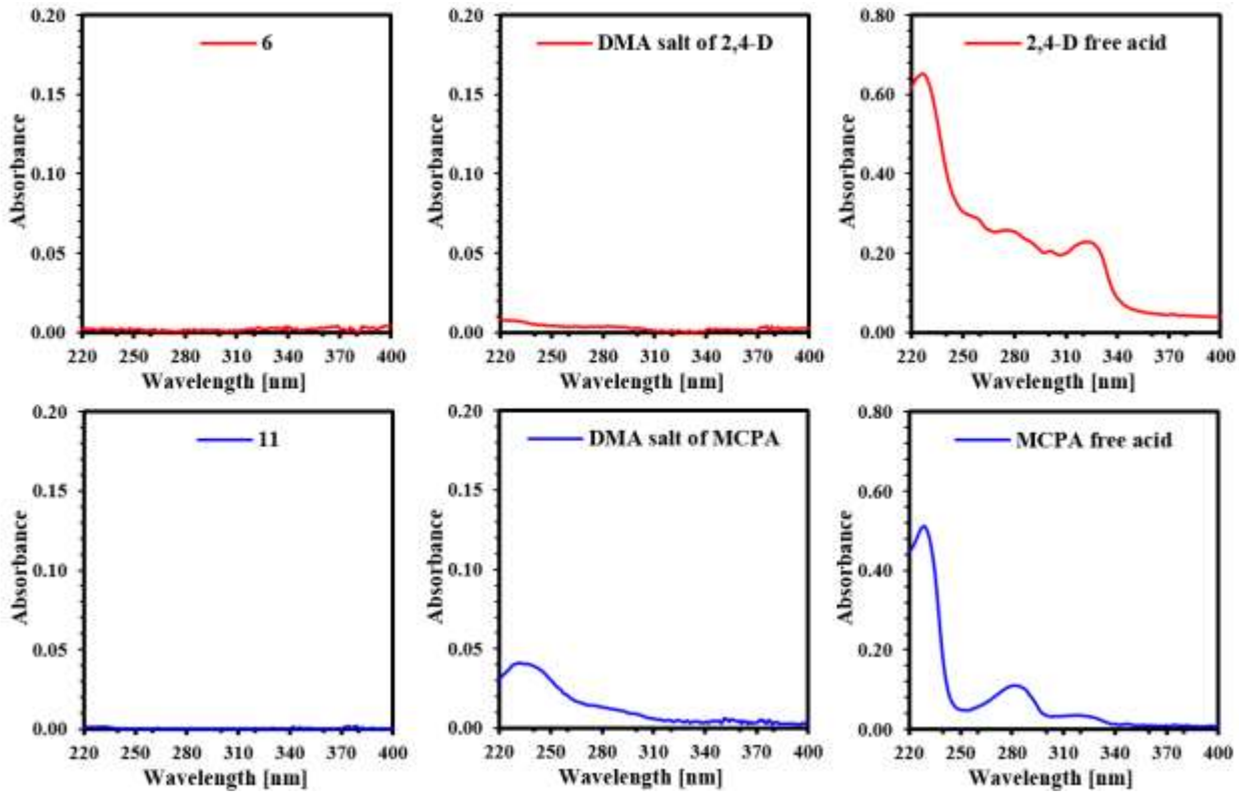

Table S30. Efficacy of the prepared salts with 2,4-D (**6**–**10**) and MCPA anion (**11**–**15**) toward cornflower and oil-seed rape compared to reference herbicides (**REF**).

**Compound**	**R**	**Active ingredient**[a]	**Fresh weight reduction [%]**	
			**cornflower**	**oil-seed rape**
**6**	$C_{10}H_{21}$	2,4-D	92.4 a[b]	86.0 a
**7**	$C_{12}H_{25}$		92.6 a	80.0 ab
**8**	$C_{14}H_{29}$		94.3 a	83.1 a
**9**	$C_{16}H_{33}$		93.2 a	85.6 a
**10**	$C_{18}H_{37}$		18.0 d	44.3 d
Aminopielik Standard (REF)			80.5 ab	53.3 cd
**11**	$C_{10}H_{21}$	MCPA	52.2 c	72.2 abc
**12**	$C_{12}H_{25}$		68.0 b	73.0 abc
**13**	$C_{14}H_{29}$		92.3 a	69.8 abcd
**14**	$C_{16}H_{33}$		92.7 a	86.1 a
**15**	$C_{18}H_{37}$		67.7 b	82.0 a
Chwastox Extra (REF)			93.2 a	56.1 bcd
LSD (0.05)			15.12	25.49

[a] All tested products were used in a dose of 400 g a.i. (active ingredient) per hectare

[b] a–d – different letters indicate statistically different mean, LSD ($p<0.05$); a – the highest efficacy of the plant protection product

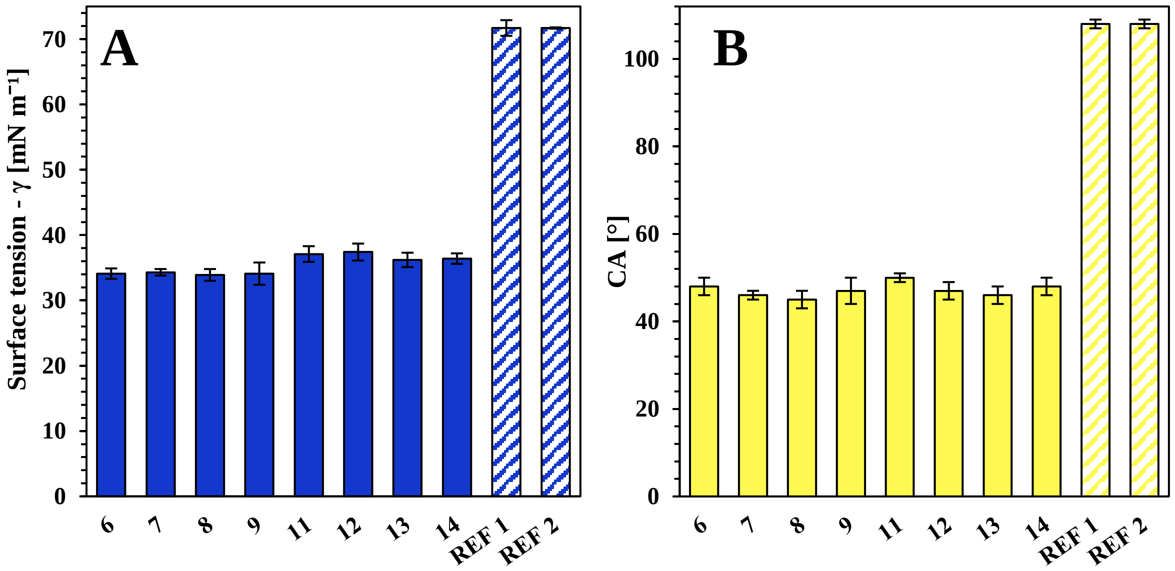

Figure S60. Surface tension (**A**) and contact angles (**B**) of the tank mixes of synthesized salts as well as reference herbicides (**REF 1** – Aminopielik Standard [containing 2,4-D]; **REF 2** – Chwastox Extra [containing MCPA]).

Table S31. Efficacy of different doses of salt comprising *N*-hexadecylnicotinamide cation and MCPA anion (**14**) in comparison to reference herbicide containing MCPA (**REF**) toward cornflower and oil-seed rape.

**Compound**	**Dose [g·ha^{-1}]**	**Fresh weight reduction [%]**	
		**cornflower**	**oil-seed rape**
**14**	200	66.4 abc[a]	33.6 def
**14**	400	67.3 abc	45.9 cdef
**14**	600	72.8 ab	61.5 bc
**14**	800	78.7 ab	71.4 ab
**14**	1000	80.1 a	88.5 a
Chwastox Extra (REF)	200	37.6 d	22.2 f
Chwastox Extra (REF)	400	48.5 cd	28.6 ef
Chwastox Extra (REF)	600	60.6 bc	29.6 ef
Chwastox Extra (REF)	800	61.3 abc	55.1 bcd
Chwastox Extra (REF)	1000	59.9 bc	49.6 bcde
LSD (0.05)		18.91	24.61

[a] a–b – different letters indicate statistically different mean, LSD ($p<0.05$); a – the highest efficacy of the plant protection product
